# A difluoroboryl radical and its boryl anion

**DOI:** 10.1039/d6sc05302e

**Published:** 2026-10-09

**Authors:** Eduardo F. S. Guimarães, Andreas Sachs, Johannes Röder, Sophia M. Schalk, Lea Völker, Lenja Wilkens, Merle Arrowsmith, Ivo Krummenacher, Sourav Kar, Eufrânio N. da Silva Júnior, Holger Braunschweig

**Affiliations:** a Institute for Inorganic Chemistry, Julius-Maximilians-Universität Würzburg Am Hubland 97074 Würzburg Germany h.braunschweig@uni-wuerzburg.de; b Institute for Sustainable Chemistry & Catalysis with Boron, Julius-Maximilians-Universität Würzburg Am Hubland 97074 Würzburg Germany; c Instituto de Ciências Exatas, Departamento de Química, Universidade Federal de Minas Gerais Belo Horizonte MG 31270-901 Brazil eufranio@ufmg.br; d Siebold-Gymnasium Rennweger Ring 11 97070 Würzburg Germany

## Abstract

The one-electron reduction of a cyclic alkyl(amino)carbene (CAAC)-stabilised trifluoroborane with Li yields the stable difluoroboryl radical [(CAAC)BF_2_]˙, which, although essentially CAAC-centred, reacts like a boron-centred one. Further reduction with Li or Na in THF yields the difluoroboryl anion [(CAAC)BF_2_]M, which in non-coordinating solvents undergoes disproportionation to the linear diboracumulene (CAAC)_2_B_2_ and [(CAAC)BF_2_]˙, the reaction being driven by the high lattice energy of the liberated MF equivalents. Reactions with iodoalkanes in Et_2_O show that [(CAAC)BF_2_]K acts as a boron nucleophile despite a positive partial charge at boron.

## Abstract 2

A cyclic alkyl(amino)carbene-stabilised trifluoroborane can be reduced stepwise to the corresponding stable and boron-centred difluoroboryl radical and boryl anion nucleophile.

## Graphical abstract

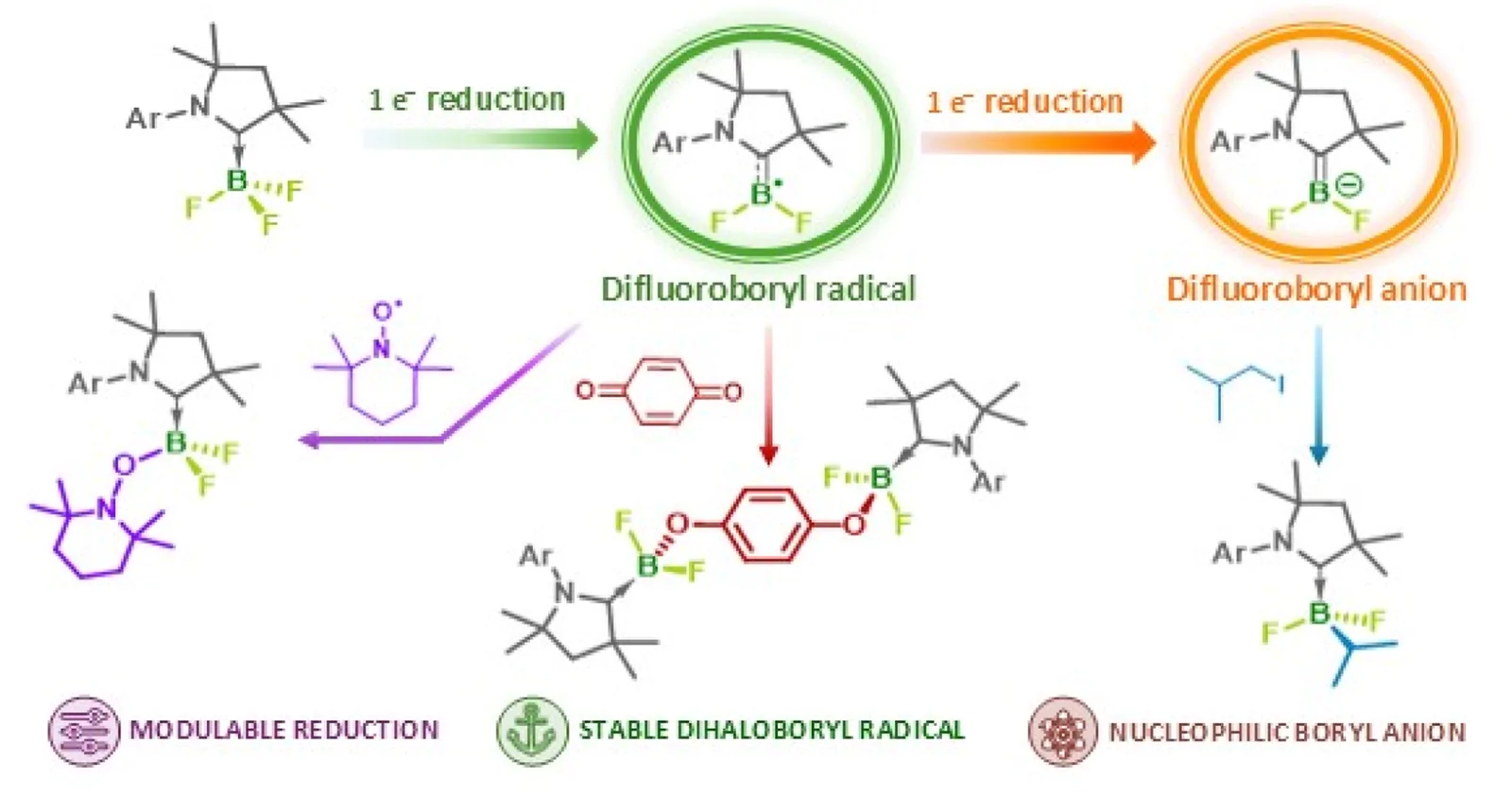

Thanks to advances in air-sensitive techniques and ligand design, the last two decades have witnessed a remarkable increase in the reports of isolable metal-free low-oxidation-state boron compounds, fuelled not only by the search for exotic structural and electronic motifs but, more and more, by the exploration of their fascinating reactivity, now reaching well beyond simple metallomimetism.^1^

Since Bertrand's landmark isolation of the first metal-free borylene in 2010 (I, Fig. 1),^2^ cyclic alkyl(amino)carbene (CAAC) ligands have proven extremely reliable for the stabilisation of unusual low-valent boron compounds. This is owed to their unique combination of strong σ-donor and π-acceptor abilities,^3^ which helps stabilise excess electron density by boron-to-CAAC π backdonation. From the first neutral boryl radical (III),^4^ dicoordinate aminoborylene (II)^5^ and linear boryl radical cation (IV),^6^ to formal boron(0) species, like the diboracumulene V^7^ or the linear anion/radical redox pair [VI]^−^/[VI]˙,^8^*via* dinitrogen activation, coupling, cleavage and functionalisation by CAAC-stabilised arylboron(i) species,^9^ these ligands have been key to advancing the field of subvalent boron chemistry.

**Fig. 1:**
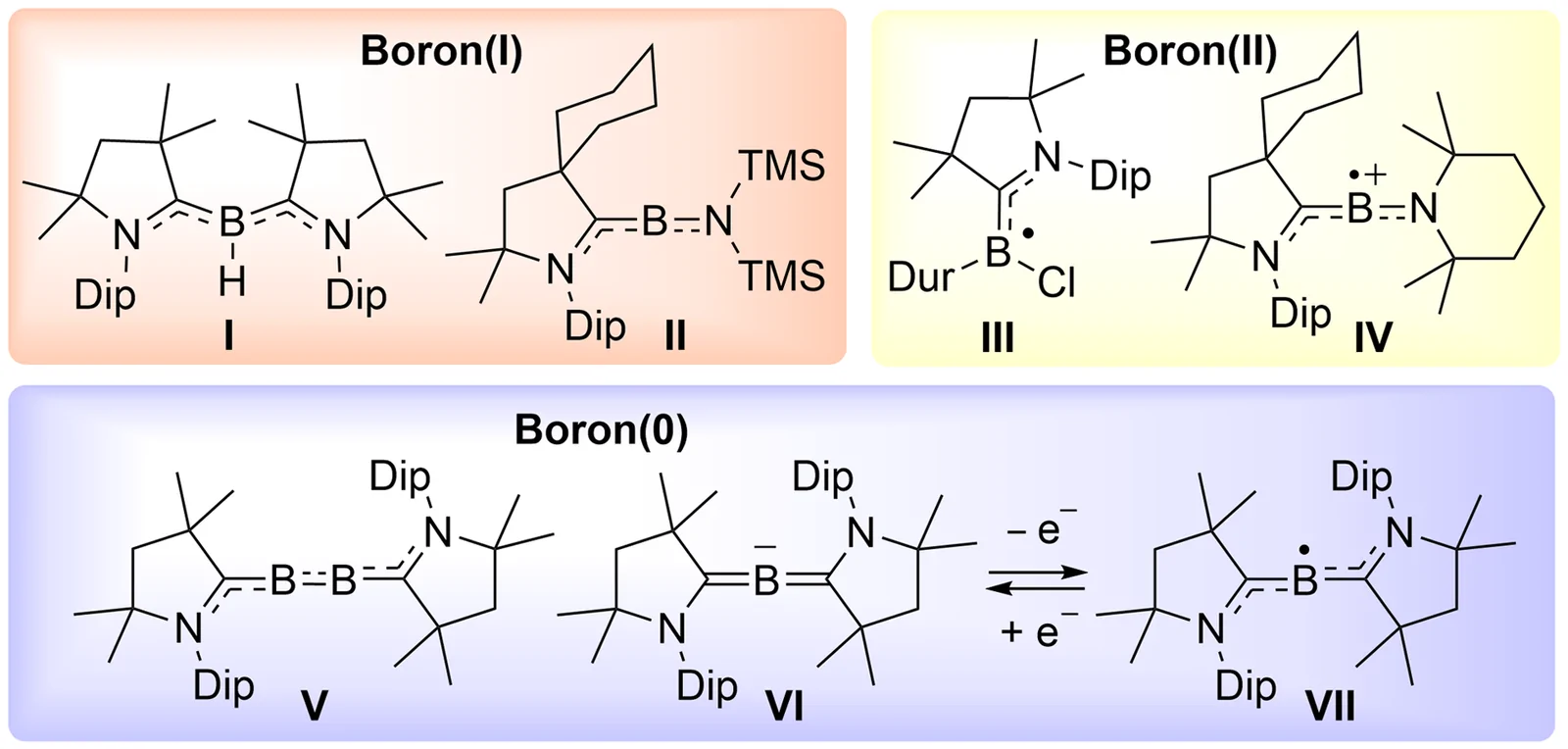
Landmark low-valent CAAC-stabilised boron compounds. Dip = 2,6-diisopropylphenyl; TMS = trimethylsilyl; Dur = 2,3,5,6-tetramethylphenyl (duryl).

Boryl radicals, in particular, require electron-withdrawing ligand systems to delocalise their otherwise highly reactive unpaired electron.^1*i*^ N-heterocyclic carbenes (NHCs), which are less π-acidic than CAACs,^10^ have been employed to generate stable cyclic boryl radicals (*e.g.* borolyl, borafluorenyl), in which the unpaired electron is mainly stabilised by π delocalisation over the boron heterocycle.^11^ Acyclic [(NHC)BYZ]˙ boryl radicals tend to be short-lived,^12^ unless stabilised by highly electron-withdrawing (*e.g.* Y/Z = *para*-trifluoromethylphenyl) or sterically demanding (*e.g.* Y/Z = 2,4,6-trimethylphenyl) substituents.^13^ Their high spin density at boron makes most NHC boryl radicals particularly prone to hydrogen atom abstraction and dimerisation, which is why they are generally only generated and used *in situ* for borylation reactions.^12,14^

In contrast, many neutral (a)cyclic [(CAAC)BYZ]˙ mono,^4,5,9,11*e*,15,16^ di-^11*b*,17,18^ and even tetraradicals,^19^ displaying a wide range of substitution patterns at boron (*e.g.* BYZ = BArX (X = Cl, Br, CN), B(SiR_3_)Cl, B(NR_2_)Cl, BCl_2_, BRH, B(BR_2_)H, B(ChPh)B(ChPh)(CAAC) (Ch = S, Se, Te), borafluorenyl, borepinyl, boraphenanthrenyl, diboraacenediyl, *etc.*), are stable enough to be isolated and fully characterised. Their stability mainly stems from the delocalisation of their unpaired electron(s) over the B–(C–N)_CAAC_ π framework, which leaves only a minor amount of spin density at boron.^20^ Further reversible one-electron reduction may yield [(CAAC)BYZ]^−^ boryl anions, in which the anionic lone pair at boron entirely π-backdonates to the CAAC carbene carbon centre, generating a B=C double bond.^9*c*,15*e*,*f*,16,18*b*–*d*,21–23^ Notably, additional stabilisation by coordination of the Y/Z substituents to the alkali metal countercation(s) has enabled the isolation of derivatives with very little steric encumbrance at boron (*e.g.* BYZ = BCl_2_, BHCl, BH_2_, BH(CN), B(CN)_2_).^21^

Gilliard has reported a number of stable π-boracyclic [(CAAC)BYZ]^−^/[(CAAC)BYZ]˙ redox pairs, such as the borafluorenyl anion/radical pair [VII]^−^/[VII]˙ (Fig. 2).^11*e*,22^ There are only a couple of known acyclic redox pairs of this type, namely the stable alkyl(hydrido)boryl anion/radical pair [VIII]^−^/[VIII]˙,^15*e*^ of which the radical is actually so sterically shielded that it is air-stable, and the dichloroboryl pair [IX]^−^/[IX]˙, of which the anion proved unstable towards chloride abstraction, and yielded only a few crystals for structural characterisation.^15*f*^ We were interested in generating a [(CAAC)BY_2_]^−^/[(CAAC)BY_2_]˙ redox pair sufficiently stable to be isolated and still unencumbered enough to be reactive. Given the stability of the B–F bond (bond dissociation energy (BDE) = 175 kcal mol^−1^),^24^ the small size of fluoride, and the 2019 report of an isolable [(CAAC)_2_BF]/[(CAAC)_2_BF]˙^+^ fluoroborylene/-boryl radical cation redox pair,^25^ our attention turned towards the synthesis of the [(CAAC)BF_2_]^−^/[(CAAC)BF_2_]˙ redox pair.

**Fig. 2:**
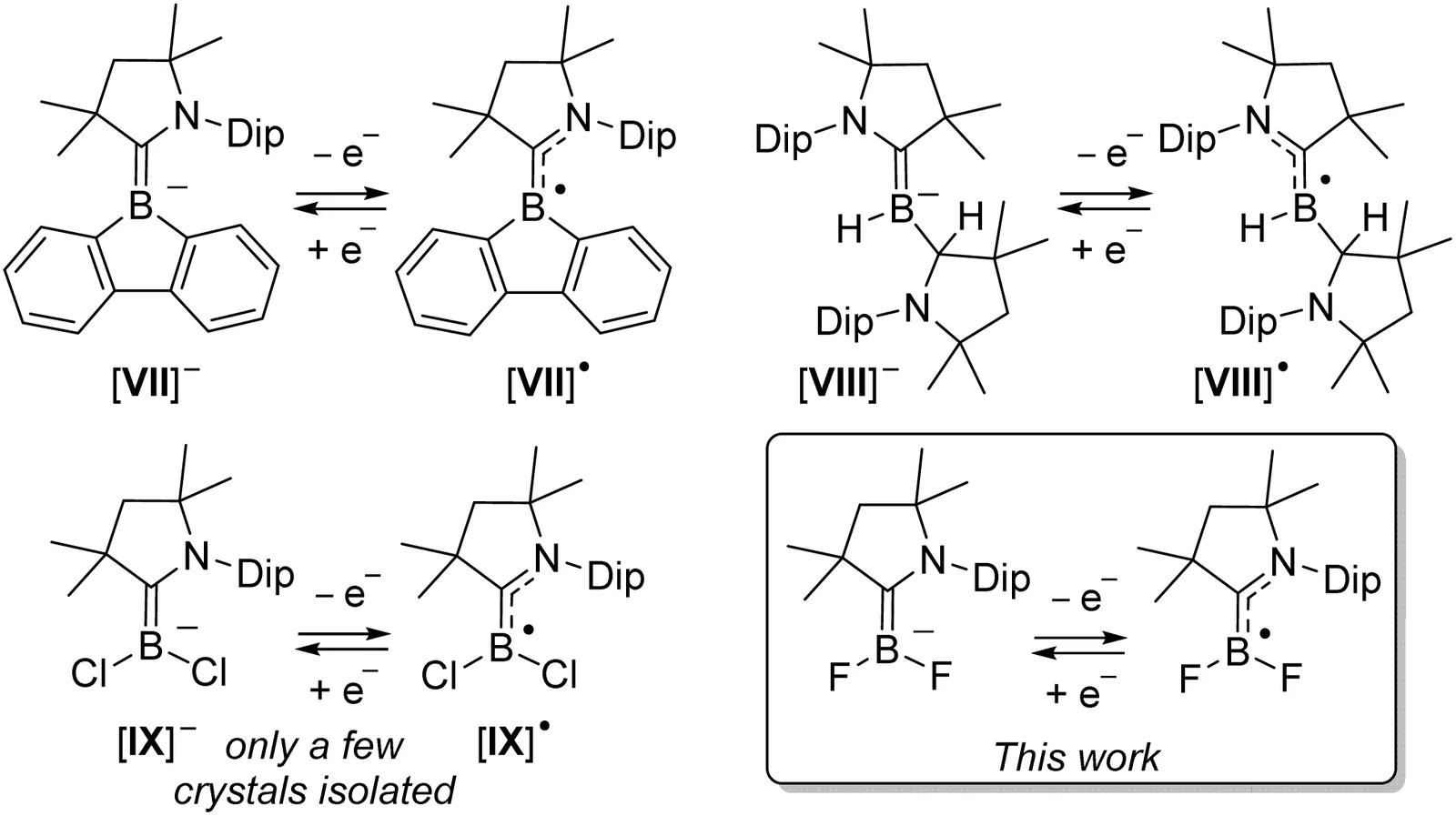
Examples of tricoordinate boryl anion/boryl radical redox pairs.

The one-electron reduction of (CAAC)BF_3_ (1)^25^ with lithium sand in THF resulted in the formation of the difluoroboryl radical [(CAAC)BF_2_]˙ (2) (Scheme 1a). The success of the reaction proved highly sensitive to the reaction conditions. When carried out in 0.5 mL THF in a sealed 5 mL glass vial in the glovebox under vigorous stirring, NMR-spectroscopic monitoring showed *ca.* 95% consumption of 1 over the course of 18 h at rt. In contrast, when carried out in a sealed Young NMR tube, using the exact same quantities of reagents and solvent, barely any reduction was observed, even when agitating on a nutator, sonicating, or heating at 80 °C for 1 day. It is also noteworthy that even when using a slight excess of Li, 1 was never fully consumed. Instead, small amounts of the two-electron reduction product 7^Li^ were observed (*vide infra*), suggesting an equilibrium between 1, 2 and 7^Li^. Unfortunately, attempts to assess the reduction potentials of 1 by cyclic voltammetry failed due to the rapid *in situ* formation of redox-active byproducts.

**Scheme 1:**
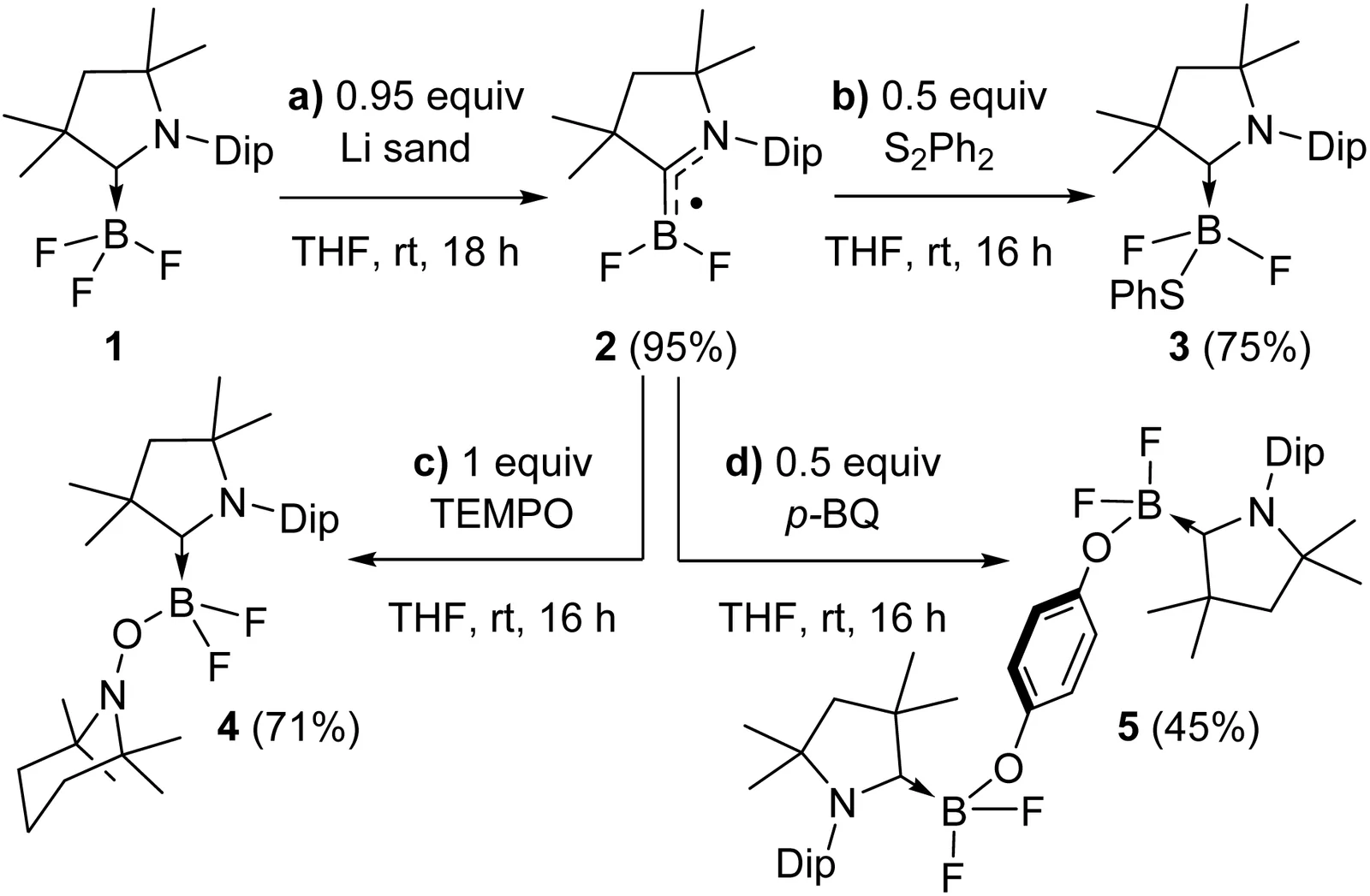
(a) Synthesis of the boryl radical 2 and (b–d) its reactions with various oxidising reagents. *p*-BQ = *para*-benzoquinone.

Radical 2 was characterised by HRMS (*m*/*z* calcd = 334.2518; found = 334.2511) and EPR spectroscopy. The EPR spectrum of 2 shows a ten-line signal with additional hyperfine features, which modelling attempts could not fully reproduce, even when refining each ^19^F hyperfine coupling separately (Fig. 3a). However, the overall line shape was adequately modelled by the following hyperfine couplings: *a*(^10/11^B) = 16.4, *a*(^14^N) = 19.5, and *a*(^19^F, 2F) = 30 MHz. The higher *a*(^10/11^B) compared to the monofluoroboryl radical cation [(CAAC)_2_BF]˙^+^ (*a*(^10/11^B) = 5.9 MHz)^25^ indicates a significantly higher spin density at boron in 2. This is mainly due to the unpaired electron being delocalised over a single CAAC ligand rather than two. In fact, the boron hyperfine coupling in 2 is in the high range for neutral CAAC-stabilised boron radicals (*a*(^10/11^B) = 3–15 MHz).^15*c*,*d*,20^

**Fig. 3:**
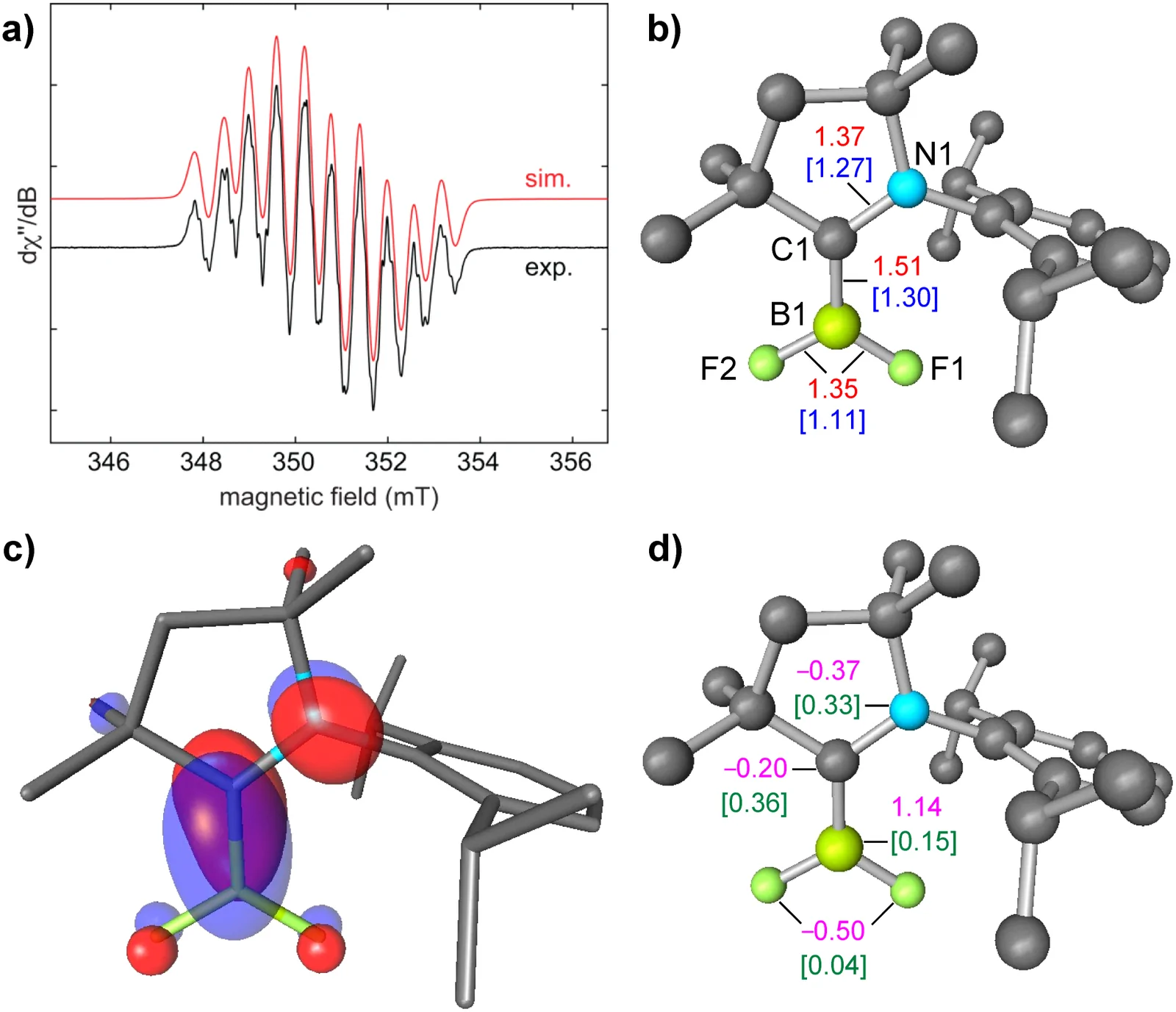
(a) Experimental (black) and simulated (red) continuous-wave (CW) X-band EPR spectra of 2 in benzene at room temperature. Best-fit simulation parameters: *g*_iso_ = 2.0033; *a*(^10/11^B) = 16.4, *a*(^14^N) = 19.5, *a*(^19^F, 2F) = 30 MHz; (b) optimised structure of 2 with calculated bond lengths (Å, red) and Wiberg bond indices (WBIs, blue) in brackets; (c) plot of the SOMO of 2 (isovalue 0.04); (d) optimised structure of 2 with calculated natural bond orbital (NBO) charges (pink) from natural population analysis (NPA) and spin densities (green) in brackets. Calculations carried out at the ωB97X-D4-def2-TZVP level of theory. Atom colours: boron = yellow-green, carbon = grey, nitrogen = sky blue, fluorine = pale green. Hydrogen atoms omitted for clarity.

Unlike the analogous NHC-bound difluoroboryl radical, [(IMe)BF_2_]˙ (IMe = 1,3-dimethylimidazol-2-ylidene), which could only be generated *in situ* and decays on the microsecond timescale,^26^ radical 2 could be isolated as a colourless viscous oil by extraction of the crude product with hexane. While recrystallisation of 2 at −30 °C from pentane yielded colourless crystals, these always melted as soon as transferred to microscopy oil for crystal selection, even under a stream of cold N_2_. Therefore, the structure of 2 could only be assessed by DFT calculations. Overall, the calculated structural parameters of 2 at the ωB97X-D4-def2-TZVP level of theory (Fig. 3b, see Computational details in the SI) resemble those of the analogous dichloroboryl radical [IX]˙.^15*f*^ The N1–C1, C1–B1 and B1–F Wiberg bond indices (WBIs)^27^ of 1.27, 1.30 and 1.11, respectively, suggest π delocalisation over the entire N1–C1–BF_2_ framework, including a small degree of F-to-B π bonding. The SOMO of 2 shows delocalisation of the unpaired electron over the entire N1–C1–BF_2_ π framework, encompassing the B1–C1 π bond, a large contribution of the N1 p_*z*_ orbital and small contributions of the fluorine p_*z*_ orbitals, with nodes at the C1–N1 and B1–F bonds (Fig. 3c). Natural population analysis (NPA)^28^ calculations provide a large positive natural bond orbital (NBO) charge at boron (1.14). More than 2/3 of the NBO spin density resides on the CAAC ligand (N1 0.33, C1 0.36), with only 15% at boron and a mere 4% at each fluorine atom (Fig. 3d). Comparison with the NBO spin densities calculated for the dichloroboryl radical [IX]˙ (N 0.29, C_carbene_ 0.29, B 0.23, Cl 0.05 each)^15*f*^ and [(IMe)BF_2_]˙ (N 0.28 each, C_carbene_ 0.14, B 0.22, F 0.05 each)^26^ at the same level of theory suggests that the higher stability of 2 is owed to its much lower amount of spin density at boron and fluorine.

In order to determine whether 2 should be considered a boron- or a CAAC-centred radical from a reactivity point of view, several one-electron oxidation reactions were carried out. The reaction of radical 2 with diphenyl disulfide (0.5 equiv.), the stable radical TEMPO (2,2,6,6-tetramethylpiperidine *N*-oxide, 1 equiv.), and *p*-BQ (*para*-benzoquinone, 0.5 equiv.) proceeded selectively to afford the corresponding boranes 3, 4 and 5, respectively, all of which were isolated as colourless crystalline solids (Scheme 1b–d). All three compounds present a *B*F_2_ triplet in the ^11^B NMR spectrum, at 5.1 ppm for 3, 1.4 ppm for 4 and 0.5 ppm for 5. It is noteworthy that the ^11^B–^19^F coupling constant in the sulfide 3 (^1^*J*_B–F_ = 70 Hz) is significantly larger than in the oxo compounds 4 and 5 (^1^*J*_B–F_ = 44–49 Hz). The corresponding ^19^F NMR resonances appear at −136.7 (q, 3), −131.6 (q, 4), and −140.8 (br m, 5), the first two being downfield- and the latter upfield-shifted from 1 (*δ*_^19^F_ = −138.7 ppm). The ^11^B and ^19^F NMR shifts of 5 are comparable to those of the related NHC analogue (IDip)BF_2_(OPh) (*δ*_^11^B_ = 0.5 ppm, *δ*_^19^F_ = −142.1 ppm, ^1^*J*_B–F_ = 47 Hz).^29^ These reactions show that, despite the low spin density at boron, 2 reacts exclusively as a boron-centred radical.

The solid-state structures of 3–5 confirm the formation of the new B–S and B–O bonds (Fig. 4). The B1–C1 bond lengths of 3 (1.6422(19) Å) and 5 (1.6589(17) Å) are slightly shorter than in 1 (1.6666(19) Å, see Fig. S36 in the SI) as the SPh and O(C_6_H_4_)O substituents are less π-donating than fluoride, whereas that of 4 with its sterically demanding and donating TEMPO substituent is slightly longer (1.685(3) Å). In all three compounds, the BF_2_Y moiety is rotated so that the B1–F1 bonds approach coplanarity with the C1 plane (|N1–C1–B1–F1| 15.2(2)–24.1(3)°) so as to maximise π overlap with the C–N π bond and minimise steric interactions with the Y substituent. As a result, the B1–F1 bond (3 1.3819(17), 4 1.392(2), 5 1.3965(15) Å) is slightly shorter than the B1–F2 bond (3 1.3893(17), 4 1.414(2), 5 1.4118(15) Å).

**Fig. 4:**
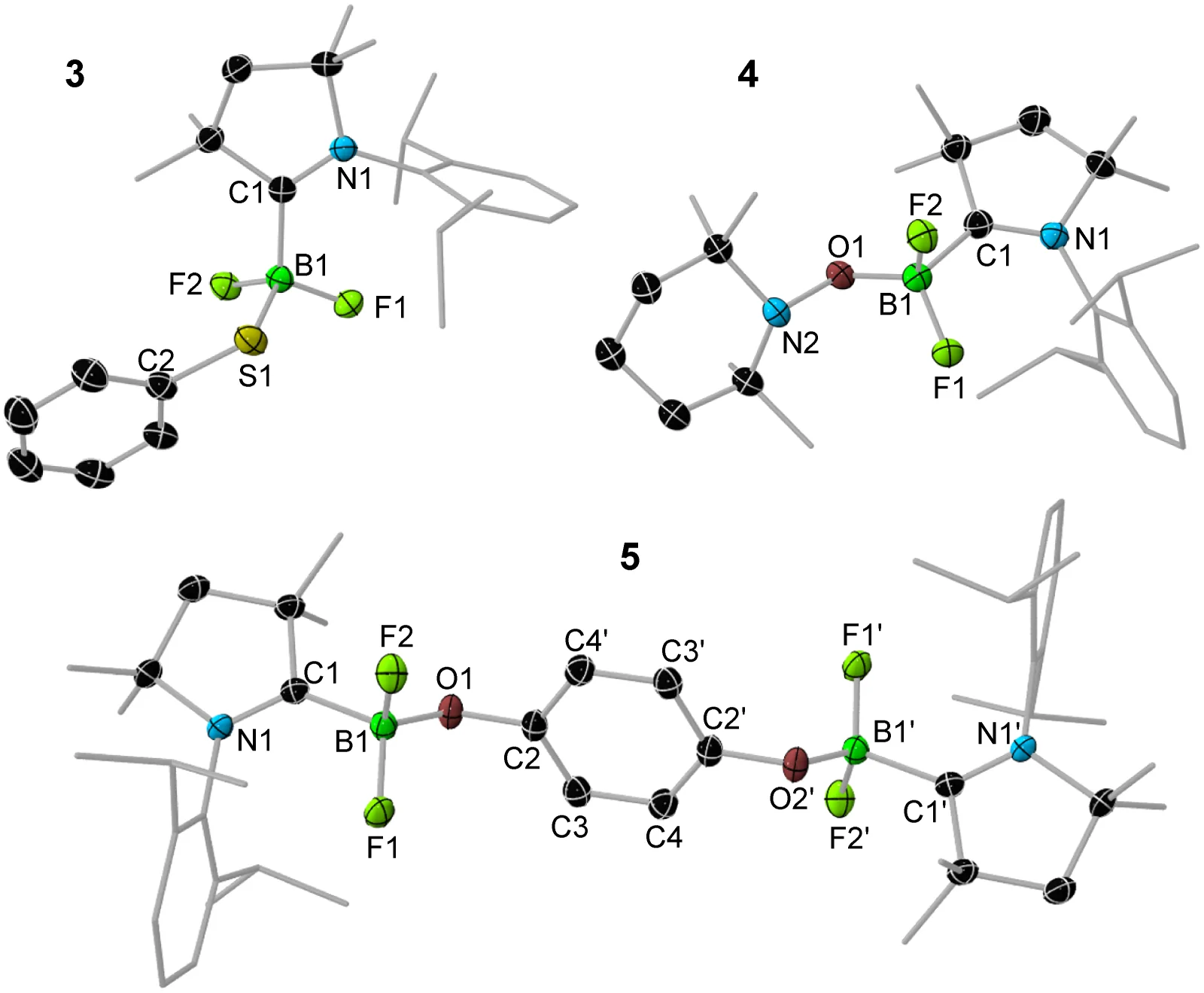
SCXRD-derived solid-state structures of 3–5. Atomic displacement ellipsoids set at 50% probability. Ellipsoids of ligand periphery and hydrogen atoms omitted for clarity.

Attempts to carry out the two-electron reduction of 1 with KC_8_ in benzene systematically yielded complex mixtures of BF-containing compounds as determined by ^11^B and ^19^F NMR spectroscopy, with compositions varying with reaction concentration and time. Recrystallisation from pentane at 30 °C yielded a small crop of red crystals of the tetranuclear complexes 6a ([(CAACH)_4_B_4_F_8_K_2_], red parts in Scheme 2a) and 6b ([(CAACH)(CAAC)_3_B_4_F_9_K_2_], blue parts), overlapping in the asymmetric unit (black parts) in a 4 : 1 ratio.‡‡The tetranuclear complexes 6a and 6b may be partitioned into various (CAAC)BF_*n*_-containing units with boron in different oxidation states: 6a = {2[(CAACH)B^III^F_3_]K + 2[(CAACH)B^II^F]˙} or {2(CAACH)B^III^F_2_ + [(CAACH)B^III^F_3_]K + [(CAACH)B^I^F]K}, average boron oxidation state +2.5; 6b = {(CAAC)B^III^F_3_ + [(CAAC)B^I^F_2_]K + [(CAACH)B^III^F_3_]K + (CAAC)B^I^F} or {(CAAC)B^III^F_3_ + 2[(CAAC)B^I^F_2_]K + (CAACH)B^III^F_2_}, average boron oxidation state +2. The component parts of 6a/6b indicate various stages of reduction from B(iii) to B(i), while the protonated CAACH ligands likely result from H-atom abstraction from the solvent by boryl radical intermediates, as observed in the reduction of (CAAC)BBr_3_ to I,^2^ or of (NHC)BBr_3_ to (NHC)_2_B_2_H_2*n*_ (*n* = 1, 2),^30^ followed by B-to-C_CAAC_ hydride shuttling, commonly observed in CAAC-stabilised hydridoboranes.^31^

**Scheme 2:**
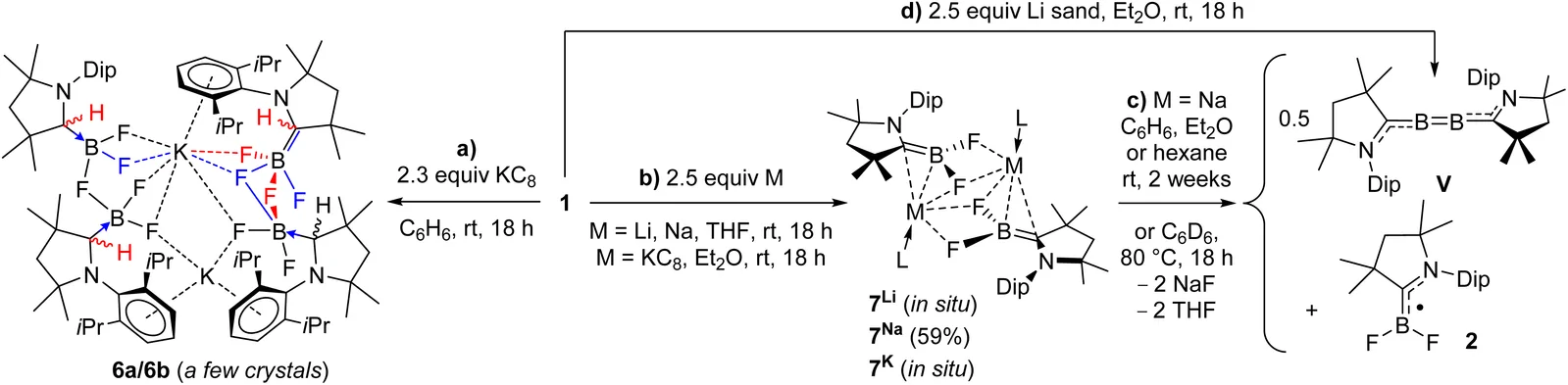
(a,b) Two-electron reductions of 1 and (c,d) disproportionation of the boryl anions 7^Li/Na^. The black parts of 6a/6b are common to both co-crystallising components, while its red and blue parts highlight the specific differences of 6a and 6b, respectively.

When carried out with lithium sand, sodium sand or KC_8_ in THF, the two-electron reduction of 1 yielded the red-orange difluoroboryl anion 7^M^ (M = Li, Na, K; Scheme 2b). The ^11^B NMR spectra of 7^M^ show a broad resonance around 28 ppm, significantly deshielded in comparison to all other acyclic [(CAAC)BYZ]^−^ boryl anions (*δ*_^11^B_ = 17 to −18 ppm),^9*c*,15*e*,21*a*,23^ and in the typical range for electron-rich tricoordinate B(iii) compounds, suggesting a relatively positive charge at boron (*vide infra*). Only a few CAAC-stabilised cyclic boryl anions, such as 1,2-diborete dianions and borepine anions, display similarly downfield-shifted ^11^B NMR resonances.^16,18*b*,*d*^ The ^19^F NMR spectra of 7^Li^ and 7^Na^ show two broad resonances in a 1 : 1 ratio around −157 and −159 ppm, *ca.* 20 ppm upfield of 1 (*δ*_^19^F_ = −138.7 ppm) or the fluoroborylene (CAAC)_2_BF (*δ*_^19^F_ = −139.4 ppm),^25^ indicating an increase in electron density at the fluorine atoms. The two distinct ^19^F NMR shifts reflect a lack of rotation about the B–C bond, making the two fluorine nuclei magnetically inequivalent, and indicate that the structure of 7^Li^ is similar to that of 7^Na^. In contrast, 7^K^ shows a single broad ^19^F NMR resonance around −152 ppm, upfield from 7^Li/Na^, suggesting a different structure.

Compounds 7^Li^ and 7^K^ did not provide single crystals suitable for SCXRD and were therefore only partly characterised and later used *in situ* (*vide infra*). Compound 7^Na^ crystallises as a pseudo-centrosymmetric dimer in a manner similar to [(CAAC)BH_2_Li(thf)]_2_,^21*a*^ with the Na^+^ cations chelated by the BF_2_ moieties (Na⋯F 2.2838(10)–2.5380(10) Å) and bridging between the two dimer halves by additional π interactions with the N–C–B–F π framework of the second [(CAAC)BF_2_]^−^ moiety (Na⋯(NCBF)_plane_*ca.* 1.13 Å). Instead of Na1 and Na2 being positioned directly above B2 and B1, respectively, as is the case for the Li^+^ cations in [(CAAC)BH_2_Li(thf)]_2_,^21*a*^ they sit offset above the F3⋯N2 and F1⋯N1 axes, respectively (Na1⋯F3 2.5719(10), Na1⋯N2 2.7673(12), Na2⋯F1 2.6223(10), Na2⋯N1 2.6275(12) Å). This results in significant distortions of N1 and N2 from planarity (Σ∠N1 346.3(1)°, Σ∠N2 346.8(1)°). The B–C bond lengths (1.430(2), 1.429(2) Å) are at the shorter end of the range observed for the rigid B=C double bonds of CAAC-stabilised boryl anions (1.43–1.48 Å).§§According to a CCDC search of the CAAC=(sp^2^-B)^−^ structural motif.^21*b*,22^ Despite the additional Na⋯F interactions, the B–F bond lengths (1.3937(17)–1.4016(17) Å) are similar to that of the fluoroborylene (CAAC)_2_BF (1.3999(15) Å),^25^ and longer than those calculated for the boryl radical 2 (1.35 Å), suggesting an absence of F-to-B π donation.

DFT calculations reproduce the structure of 7^Na^ with good accuracy. The B–C WBIs (avg. 1.85) indicate a strong double bond, while C–N and B–F WBIs below unity indicate single bonds. The HOMO and HOMO−1 of 7 correspond essentially to the two B=C π bonds, with an important π-antibonding contribution of the N_CAAC_ p_*z*_ orbital and very small ones from the fluorides. The optimised structure of the naked [(CAAC)BF_2_]^−^ anion confirms that the pyramidalisation of the CAAC nitrogen atoms in the solid-state structure of 7^Na^ is entirely owed to their interaction with the Na^+^ cations (Fig. 5c). Comparison of the NBO charges of 2 and the naked [(CAAC)BF_2_]^−^ anion (Fig. 3d and 5c) shows that most of the additional negative charge accumulates at the CAAC carbene carbon (−0.35). The remaining additional charge is spread over the nitrogen (−0.23) and fluoride (−0.12) centres, while the boron centre becomes only minimally more negative (−0.08). Compared to the NBO charges at boron calculated for other known [(CAAC)BY_2_]^−^ boryl anions (Y = Cl ([IX]^−^), +0.43; Y = CN, +0.04; Y = H, −0.12, see Fig. S45 in the SI), the partial charge at boron in [(CAAC)BF_2_]^−^ is significantly more positive (+1.06), as also reflected by its unusual downfield ^11^B NMR shift of 27.7 ppm (*vide supra*).

**Fig. 5:**
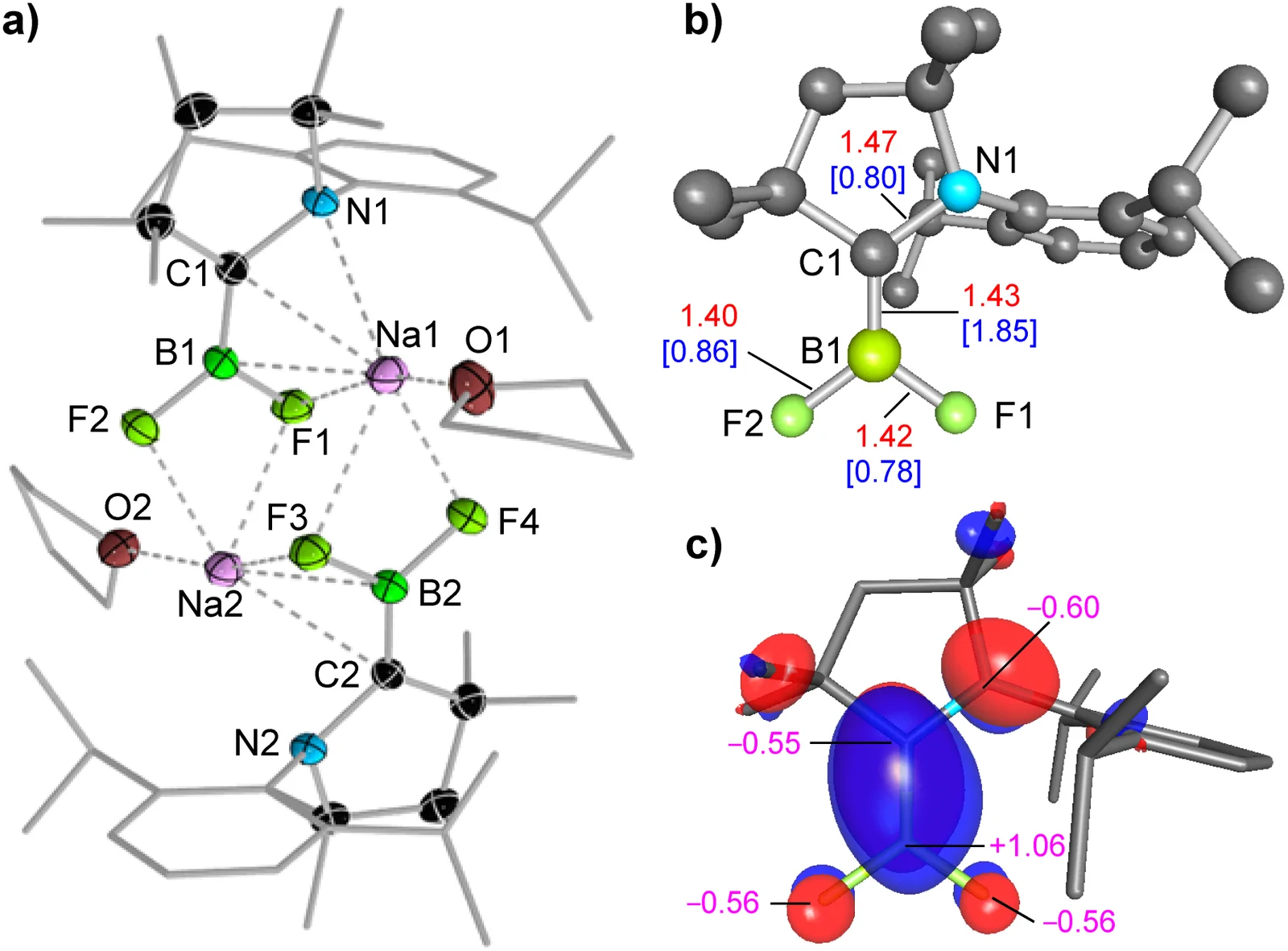
(a) SCXRD-derived solid-state structures of 7^Na^. Atomic displacement ellipsoids set at 50% probability. Ellipsoids of ligand periphery and hydrogen atoms omitted for clarity; (b) optimised structure of 7^Na^ (ball-and-stick model). Only one [(CAAC)BF_2_]^−^ moiety shown for clarity. Average calculated bond lengths (Å, red) and Wiberg bond indices (WBIs, blue) in brackets; (c) plot of the HOMO of the isolated [(CAAC)BF_2_]^−^ anion (stick model, isovalue 0.04) with calculated NBO charges (pink). Calculations carried out at the ωB97X-D4-def2-TZVP level of theory. Atom colours: boron = yellow-green, carbon = grey, nitrogen = sky blue, fluorine = pale green. Hydrogen atoms omitted for clarity.

Attempts to monomerise the difluoroboryl anion by adding 15-crown-5 or TMEDA to 7^Na^ did not result in any observable reaction. While 7^Na^ remained stable in the solid state or in THF at rt over several weeks, recrystallisation attempts from benzene, hexane or Et_2_O over 2 weeks at rt surprisingly yielded deep-purple solutions and large crystals of the same colour, which an SCXRD analysis identified as the diboracumulene V (Scheme 2c). NMR- and EPR-spectroscopic monitoring of a C_6_D_6_ solution of 7^Na^ over two weeks at rt confirmed its slow but relatively selective disproportionation into V and the boryl radical 2, with release of THF.¶¶The ^1^H NMR spectrum showed the formation of *ca.* 80% V, alongside several minor unidentified NMR-active byproducts, while the EPR spectrum showed 2 as the sole paramagnetic product. Full disproportionation, albeit slightly less selective, could also be achieved by heating 7^Na^ in C_6_D_6_ overnight at 80 °C. Interestingly, attempts to synthesise 7^Li^ in Et_2_O led directly to a mixture of V and 2, presumably as Et_2_O is too weak a Lewis base to stabilise the intermediate boryl anion (Scheme 2d). In contrast, the two-electron reduction of 1 with KC_8_ in Et_2_O stopped at the boryl anion 7^K^. Compound V could easily be isolated from the V/2 disproportionation mixture by fractional crystallisation. This is particularly interesting, as the usual route to V^7^ requires the use of extremely temperature-sensitive and highly corrosive B_2_Br_4_,^35^ or more stable but still corrosive and pungent B_2_Br_4_(SMe_2_)_2_,^36^ neither of which is commercially available. The new synthetic pathway to V by direct reduction of 1 with Li in Et_2_O circumvents the onerous synthesis of a diborane(4) reagent since 1 is obtained from commercially available (Et_2_O)BF_3_.^25^ Moreover, the smooth reductive coupling of 1 to V comes as a surprise since the reduction of CAAC-stabilised haloboranes usually leads to the formation of borylenes rather than B–B coupling,^1*j*,2^ except in the cases of (CAAC)BHX_2_ (X = Cl, Br) and the ferrocenediyl-bridged [{(CAAC)BBr_2_Cp}_2_Fe], which can be reduced to the corresponding diborenes (CAAC)_2_B_2_H ^21*b*^ and [{(CAAC)BBCp}_2_Fe],^37^ respectively.

While redox disproportionation is a common feature of aluminium(i) and gallium(i) compounds,^32^ mixtures of low-valent boron compounds of different oxidation states are rather prone to comproportionation. Indeed, a number of redox comproportionation reactions of low-oxidation (di)boron compounds have been reported.^15*e*,33^ The reactions presented in Scheme 2c and d are, to our knowledge, the first examples of spontaneous disproportionation of a boron(i) dimer into a diboron(0) and a boron(ii) radical, and highlight how unusually stable the diboracumulene V and radical 2 are compared to other formal B(0) and B(ii) species. Given the heterogeneous and radical nature of the reaction, it was not possible to obtain meaningful kinetic data for this reaction, but simple thermodynamic calculations at the ωB97X-D4-def2-TZVP-COSMO(C_6_H_6_) level of theory for the disproportionation of 7^Na^ reveal that the driving force of the disproportionation resides in the high lattice energy of NaF (*E*_lat_ = 441 kcal mol^−1^).^34^ Without taking *E*_lat_ into account, the disproportionation would actually be close to thermodynamic equilibrium (Δ*G*_298 K_*ca.* −2 kcal mol^−1^), whereas taking *E*_lat_ into account makes the reaction exothermic by −443 kcal mol^−1^ (see Table S1 in the SI).

Boryl anions are known to be excellent boron nucleophiles, their reactions with electrophiles enabling the formation of new boron–element bonds.^38^ Several CAAC- and NHC-stabilised boryl anions have also demonstrated nucleophilic reactivity at boron.^21*a*,*c*,*d*,22,39^ The highly positive charge at boron in the isolated [(CAAC)BF_2_]^−^ anion (+1.06), however, suggests a boron electrophile rather than a nucleophile. Reactions of *in situ*-generated 7^Li^ in THF with a range of chloro and iodo electrophiles (RX) known to undergo salt metathesis with other boryl anions (CH_2_Cl_2_, Me_3_SiCl, MeI, *n*PrI, iPrI) resulted in highly unselective reactions involving, among others, regeneration of 1, oxidation to (CAAC)BF_2_X, nucleophilic reactivity to yield (CAAC)BF_2_R (8^R^), and various THF ring-opening reactions, as determined by NMR spectroscopy and SCXRD analyses of various crystallisation fractions (Scheme 3a). In contrast, *in situ*-generated 7^K^ in diethyl ether reacted cleanly with *n*PrI and iPrI to yield the corresponding addition products 8*^n^*^Pr^ and 8^iPr^ (*δ*_^11^B_ = 6 ppm, *δ*_^19^F_ = −153.6, −156.5 ppm, respectively), which were also characterised by SCXRD analyses (Scheme 3b). These preliminary results show that, under THF-free reaction conditions, [(CAAC)BF_2_]^−^ acts as a boron nucleophile with electrophiles containing a good leaving group, such as iodide.

**Scheme 3:**
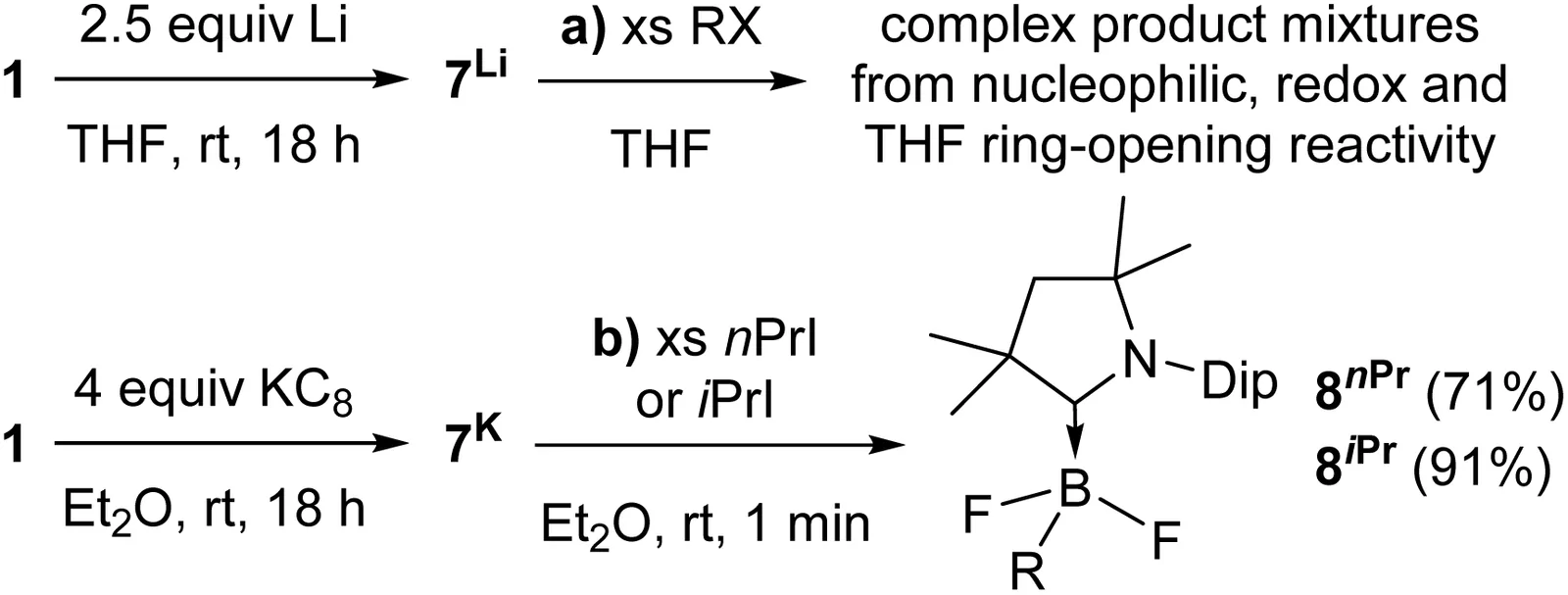
Reactivity of (a) 7^Li^ and (b) 7^K^ with various chloro and iodo nucleophiles. RX = CH_2_Cl_2_, Me_3_SiCl, MeI, *n*PrI, iPrI.

To conclude, we have synthesised the first stable dihaloboryl anion/radical redox couple, [(CAAC)BF_2_]^−^/[(CAAC)BF_2_]˙ (7/2) which owes its stability to the interplay of the π-accepting CAAC ligand and the electron-withdrawing/π-donating fluoride substituents. Although only 15% of the spin density of 2 resides at the boron centre, it reacts exclusively as a boron-centred radical. The boryl anions 7^Li^ and 7^Na^, while stable in THF and in the solid-state as cation-bridged dimers, undergo a unique and selective disproportionation reaction in non- or weakly coordinating solvents to the linear diboracumulene (CAAC)_2_B_2_ (V) and radical 2. Despite the high calculated positive partial charge at boron, *in situ*-generated 7^K^ in diethyl ether reacts as a boron-centred nucleophile towards alkyl iodides. We are continuing to explore the reactivity of the [(CAAC)BF_2_]^−^ anion and will report our results in due course.

## Author contributions

EFSG, AS, JR, SMS, LV and LW performed the experimental work. IK performed EPR spectroscopy. AS, SK and MA performed SCXRD analyses. EFSG, AS, JR and MA designed the study and wrote the SI. MA wrote the manuscript. ENSJ and HB provided funding and supervisions.

## Conflicts of interest

There are no conflicts to declare.

## Supplementary Material

SC-OLF-D6SC05302E-s001

## Data Availability

CCDC 2564348–2564353 (4, 6, 5, 3, 1 and 7^Na^), 2588263 (8^iPr^) and 2588264 (8*^n^*^Pr^) contain the supplementary crystallographic data for this paper.^40*a*–*h*^ The data supporting this article have been included as part of the supplementary information (SI). Supplementary information: synthetic procedures, NMR and EPR spectra, X-ray crystallographic and computational details. See DOI: https://doi.org/10.1039/d6sc05302e.
