# Supplementary material for: A difluoroboryl radical and its boryl anion
Source: Chem Sci. 2026 Oct 9. Online ahead of print. doi: 10.1039/d6sc05302e (PMC13689737; doi:10.1039/d6sc05302e)
Supplement: Supplementary file 1 [file SC-OLF-D6SC05302E-s001.pdf]

Supplementary Information (SI) for Chemical Science.
This journal is © The Royal Society of Chemistry

Supporting Information for:

A difluoroboryl radical and its boryl anion

Eduardo F. S. Guimarães,[‡,a,b,c] Andreas Sachs,[‡,a,b] Johannes Röder,[‡,a,b] Sophia M. Schalk,[d] Lea Völker,[d] Lenja Wilkens,[d] Merle Arrowsmith,[a,b] Ivo Krummenacher,[a,b] Sourav Kar,[a,b] Eufrânio N. da Silva Júnior,*[c] and Holger Braunschweig*[a,b]

[a] *Institute for Inorganic Chemistry, Julius-Maximilians-Universität Würzburg, Am Hubland, 97074 Würzburg, Germany.* [b] *Institute for Sustainable Chemistry & Catalysis with Boron, Julius-Maximilians-Universität Würzburg, Am Hubland, 97074 Würzburg, Germany.* [c] *Instituto de Ciências Exatas, Departamento de Química, Universidade Federal de Minas Gerais, Belo Horizonte, MG, 31270-901, Brazil.* [d] *Siebold-Gymnasium, Rennweger Ring 11, 97070 Würzburg.*

Contents

Methods and materials 2
Synthetic procedures 3
NMR spectra 10
  Isolated compounds 10
  In-situ generation of **6a/b**, $\mathbf{7^{Li}}$ and $\mathbf{7^{K}}$ 36
  Disproportionation of $\mathbf{7^{Na}}$ into cumulene **V** and radical **2** 42
EPR spectra 44
SCXRD details 45
Computational details 56
Cartesian coordinates of optimised compounds 58
References 75

Methods and materials

Synthetic and analytical methods. All manipulations were performed under an atmosphere of dry argon or in vacuo using standard Schlenk line or glovebox techniques. NMR spectra were acquired at 25 °C on a Bruker Avance 500 NMR spectrometer (^{1}H: 500.1 MHz, $^{13}C\{^{1}H\}$: 125.8 MHz, ^{11}B: 160.5 MHz, ^{19}F: 470.6 MHz, ^{23}Na: 132.3 MHz). Chemical shifts (δ) are given in ppm and internally referenced to the carbon nuclei ($^{13}C\{^{1}H\}$) or residual protons (^{1}H) of the solvent, and/or external reference ($SiMe_4$: ^{1}H, $^{13}C\{^{1}H\}$; $BF_3 \cdot OEt_2$: ^{11}B, $CFCl_3$: ^{19}F, NaCl: ^{23}Na). NMR multiplicities are given as s (singlet), d (doublets), sept (septets), m (multiplets) or br (broad). ^{n}J NMR coupling constants indicate ^{1}H-^{1}H coupling unless specified otherwise. Elemental analyses (C, H, N, S) were performed on an Elementar vario MICRO cube elemental analyzer. High-resolution mass spectrometry (HRMS) data were obtained from a Thermo Scientific Exactive Plus spectrometer. EPR measurements were recorded on a Bruker ELEXSYS E580 CW EPR X-band spectrometer. The spectral simulations were performed using MATLAB R2020a and the EasySpin 5.2.30 toolbox.[1]

Solvents and reagents. Solvents and reagents were purchased from Sigma Aldrich, Alfa Aesar, Fluorochem or TCI and transferred to the glovebox for use. Deuterated solvents were dried over molecular sieves and degassed by three freeze-pump-thaw cycles before use. All other solvents were distilled and degassed from appropriate drying agents. Solvents (both deuterated and non-deuterated) were stored under argon over activated 4 Å molecular sieves. Compound **1**,[2] and KC_8[3] were synthesised following literature procedures.

Synthetic procedures

Synthesis of 2

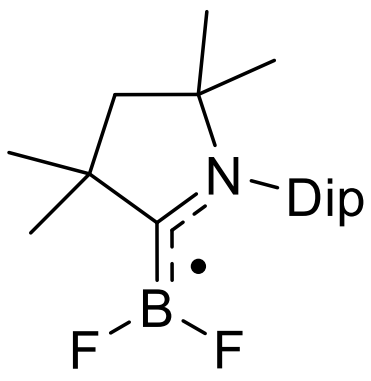

Compound **1** (0.20 g, 0.57 mmol, 1.0 equiv.) and lithium sand (3.8 mg, 0.53 mmol, 0.9 equiv.) were combined in THF (0.5 mL) at room temperature (rt), and the mixture was vigorously stirred until complete consumption of the metallic lithium, during which the solution turned dark blue. After removal of the solvent under reduced pressure, the resulting residue was extracted with pentane (0.5 mL) to remove unreacted **1** and LiF, and dried in vacuo, yielding **2** as a colourless oil (0.18 g, 0.57 mmol, 95%). Storage at –30 °C of the resulting dark grey solution overnight repeatedly yielded colourless crystals of **2**, which however, melted systematically when attempting isolation or transfer to a microscopy slide for crystal selection. Thus radical **2** was only characterised by EPR spectroscopy in solution, cyclic voltammetry, and HRMS. EPR (pentane, 298 K): g_{iso} = 2.0033; $a(^{10/11}B)$ = 16.4, $a(^{14}N)$ = 19.5, $a(^{19}F$, 2F) = 30 MHz. HRMS (LIFDI-pos., hexane) for $[C_{20}H_{31}BF_2N]^+$ = $[M]^+$: *m*/*z* calcd. = 334. 2518; found = 334.2511.

<u>General procedure for reactions of 2 with oxidising reagents</u>

Compound **1** (75.0 mg, 212 µmol, 1.00 equiv.) was dissolved in THF (1 mL). Lithium sand (10 wt% in NaF, 14.0 mg, 202 µmol, 0.950 equiv.) was added and the reaction mixture stirred for 16 h at rt. The now dark-blue solution was filtered, treated with the oxidising reagent, and stirred for 4 h at rt, during which the colour changed to pale yellow. See syntheses of **3**-**5** below for quantities of the oxidising reagents and details of product workup.

Synthesis of 3

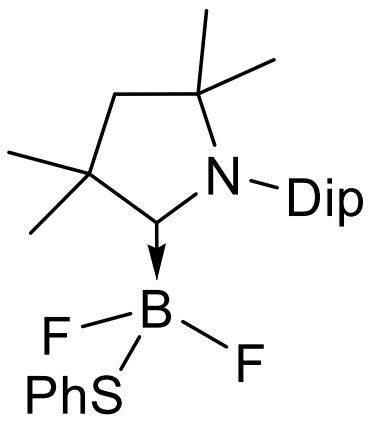

Oxidising reagent: diphenyl disulfide (23.2 mg, 106 µmol, 0.500 equiv.). After removal of volatiles *in vacuo* the yellow residue was washed with *n*-hexane (2×1 mL) and the product extracted with toluene (2×1 mL). Recrystallisation from toluene at –30 °C yielded **3** as a colourless crystalline solid (70.5 mg, 159 µmol, 75%), with crystals suitable for SCXRD analysis. ^{1}H NMR (500.1 MHz, C_6D_6): δ = 7.95-7.91 (m, 2H, *o*-Ph-*H*), 7.08-7.04 (m, 3H, *m*-Ph-*H* + *p*-Dip-*H*), 6.96 (d, 2H, ^3J = 7.5 Hz, *m*-Dip-*H*), 6.92 (tt, 1H, ^3J = 7.4 Hz, ^4J = 1.4 Hz, *p*-Ph-*H*), 2.73 (sept, 2H, ^3J = 6.7 Hz, *i*Pr-C*H*), 1.60 (s, 6H, C(CH_3)$_2$), 1.42 (d, 6H, ^3J = 6.7 Hz, *i*Pr-CH_3), 1.39 (s, 2H, CH_2), 1.11 (d, 6H, ^3J = 6.7 Hz, *i*Pr-CH_3), 0.78 (s, 6H, NC(CH_3)$_2$) ppm. ^{13}C{^{1}H} NMR (125.8 MHz, C_6D_6): δ =

218.6 (B*C*, detected by HMBC), 145.0 (*o*-Dip-*C*), 138.7 (*o*-Ph-*C*, detected by HMBC), 133.7 (*o*-Ph-*C*), 132.9 (*i*-Dip-*C*, detected by HMBC), 129.7 (*p*-Dip-*C*), 128.4 (*m*-Ph-*C*, detected by HSQC), 124.8 (*m*-Dip-*C*), 124.6 (*p*-Ph-*C*), 79.5 (N*C*Me$_2$), 52.4 (C*C*Me$_2$, detected by HMBC), 51.0 (*C*H$_2$), 29.7 (*i*Pr-*C*H), 29.5 (CC(*C*H$_3$)$_2$), 28.2 (NC(*C*H$_3$)$_2$), 26.8 (*i*Pr-*C*H$_3$), 23.7 (*i*Pr-*C*H$_3$) ppm. ^{11}B{^{1}H} NMR (160.5 MHz, C_6D_6): δ = 5.1 (t, $^1J_{B\text{-}F}$ = 70 Hz, *B*F$_2$) ppm. ^{19}F NMR (470.6 MHz, C_6D_6): δ = −136.7 (q, $^1J_{B\text{-}F}$ = 70 Hz, B*F$_2$*) ppm. Elemental (C, H, N, S) analysis (%) for [$C_{26}H_{36}BF_2NS$] (M_w = 443.4 g mol^{-1}): calcd. C 70.42, H 8.18, N 3.16, S 7.23; found: C 70.38, H 8.17, N 3.23, S 6.34. *Note: LIFDI-HRMS only yielded the mass peak for [(CAAC)BF$_2$]$^+$.*

Synthesis of 4

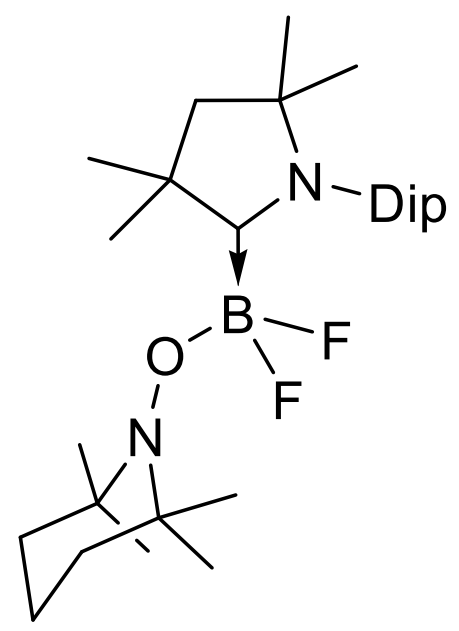

Oxidising reagent: TEMPO (33.2 mg, 212 µmol, 1.00 equiv.). After removal of volatiles *in vacuo* the yellow residue was washed with *n*-hexane (2×1 mL) and the product extracted with toluene (2×1 mL). Recrystallisation at –30 °C from toluene yielded **4** as a colourless crystalline solid (74.0 mg, 151 µmol, 71%), with crystals suitable for SCXRD analysis. ^{1}H NMR (500.1 MHz, C_6D_6): δ = 7.07 (dd, ^3J = 7.0, 8.3 Hz, 1H, *p*-Dip-C*H*), 7.00 (two overlapping d, ^3J = 7.0, 8.3 Hz, 2H, *m*-Dip-C*H*), 2.74 (sept, ^3J = 6.6 Hz, 2H, *i*Pr-C*H*), 1.73 (s, 6H, C(C*H$_3$*)$_2$), 1.62 (v. br, 6H, Tmp-CH$_2$), 1.48 (br s, 12H, Tmp-C*H$_3$*), 1.44 (d, 6H, ^3J = 6.6 Hz, *i*Pr-C*H$_3$*), 1.43 (s, 2H, C*H$_2$*), 1.13 (d, 6H, ^3J = 6.6 Hz, *i*Pr-C*H$_3$*), 0.82 (s, 6H, NC(C*H$_3$*)$_2$) ppm. ^{13}C{^{1}H} NMR (125.8 MHz, C_6D_6): δ = 225.5 (br, B*C*), 144.8 (*o*-Dip-*C*), 132.9 (*i*-Dip-*C*), 129.1 (*p*-Dip-*C*), 124.5 (*m*-Dip-*C*), 79.8 (CAAC-N*C*Me$_2$), 58.8 (Tmp-*C*$_q$), 52.2 (CAAC-C*C*Me$_2$), 50.9 (CAAC-*C*H$_2$), 41.1 (Tmp-*C*H$_2$), 29.2 (*i*Pr-*C*H), 28.5 (CC(*C*H$_3$)$_2$), 28.4 (NC(*C*H$_3$)$_2$), 26.7 (t, $^6J_{C\text{-}F}$ = 3.4 Hz, *i*Pr-*C*H$_3$), 23.8 (*i*Pr-*C*H$_3$), 17.7 (*p*-Tmp-*C*H$_2$) ppm. ^{11}B{^{1}H} NMR (160.5 MHz, C_6D_6): δ = 1.4 (t, $^1J_{B\text{-}F}$ = 49 Hz, *B*F$_2$) ppm. ^{19}F NMR (470.6 MHz, C_6D_6): δ = –131.6 (q, $^1J_{B\text{-}F}$ = 49 Hz, B*F$_2$*) ppm. HRMS (LIFDI-pos., toluene, *m*/*z*) for [$C_{29}H_{49}BF_2N_2O$]$^+$ = [M]$^+$: *m*/*z* calcd. = 490.3901; found = 490.3874.

Synthesis of 5

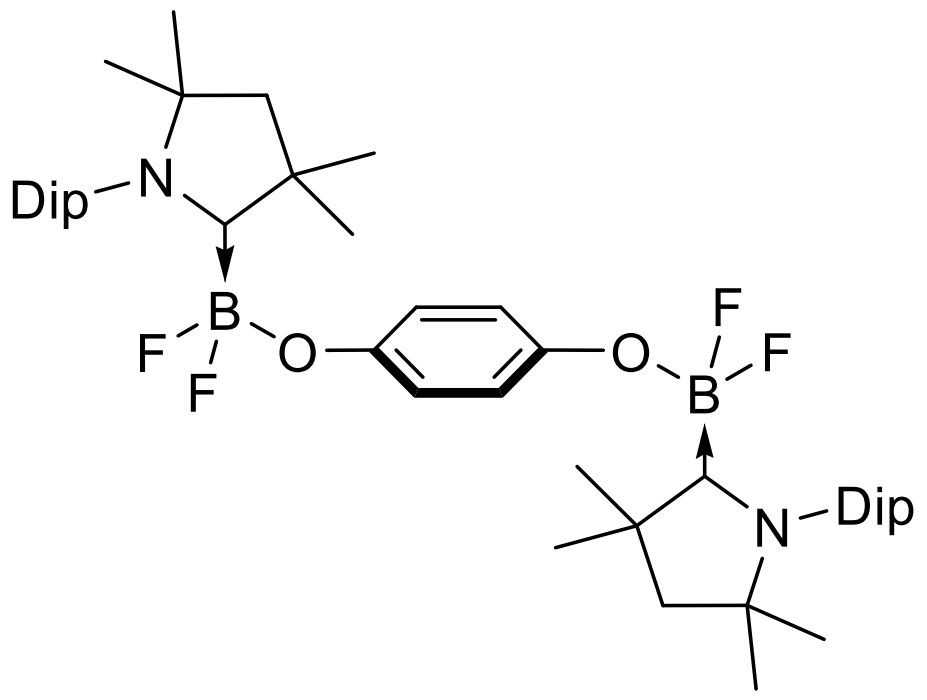

Oxidising reagent: *para*-benzoquinone (11.5 mg, 106 µmol, 0.500 equiv.). The reaction was accompanied by the precipitation of a colourless solid. After filtration, the solid residue was washed with THF (2 × 0.5 mL) and *o*-difluorobenzene (DFB, 2×0.5 mL), and dried in vacuo, yielding **5** as a colourless solid (37.0 mg, 47.6 µmol, 45%). Single crystals suitable for SCXRD analysis were obtained by slow evaporation of a saturated THF solution at −30 °C. ^{1}H NMR (500.1 MHz, C_6D_6): δ = 7.36 (t, 2H, ^{3}J = 7.8 Hz, *p*-Dip-*H*), 7.21 (d, 4H, ^{3}J = 7.8 Hz, *m*-Dip-*H*), 6.39 (s, 4H, Bz-*H*), 2.69 (sept, 4H, ^{3}J = 6.8 Hz, *i*Pr-C*H*), 2.09 (s, 4H, CH_2), 1.68 (s, 12H, CC(CH_3)$_2$), 1.37 (s, 12H, NC(CH_3)$_2$), 1.28 (d, 12H, ^{3}J = 6.8 Hz, *i*Pr-CH_3), 1.15 (d, 12H, ^{3}J = 6.8 Hz, *i*Pr-CH_3) ppm. ^{13}C{^{1}H} NMR (125.8 MHz, C_6D_6): δ = 221.5 (B*C*, detected by HMBC), 148.8 (Bz-C_q), 144.8 (*o*-Dip-*C*), 132.8 (*i*-Dip-*C*), 129.4 (*p*-Dip-*C*), 124.5 (*m*-Dip-*C*), 119.0 (Bz-*C*H), 80.2 (N*C*Me$_2$), 52.7 (C*C*Me$_2$), 51.3 (*C*H$_2$), 29.6 (*i*Pr-*C*H), 28.9 (NC(*C*H$_3$)$_2$), 28.8 (CC(*C*H$_3$)$_2$), 25.8 (*i*Pr-*C*H$_3$), 23.8 (*i*Pr-*C*H$_3$) ppm. ^{11}B{^{1}H} NMR (160.5 MHz, C_6D_6): δ = 0.5 (br t, $^{1}J_{B\text{-}F}$ = 44 Hz, *B*F$_2$) ppm. ^{19}F NMR (470.6 MHz, C_6D_6): δ = −140.8 (br m) ppm. HRMS (LIFDI-pos., THF) for $[C_{46}H_{66}B_2F_4N_2O_2]^+$ = $[M]^+$: *m/z* calcd. = 776.5241; found = 776.5227.

Attempted two-electron reduction of 1 with KC_8

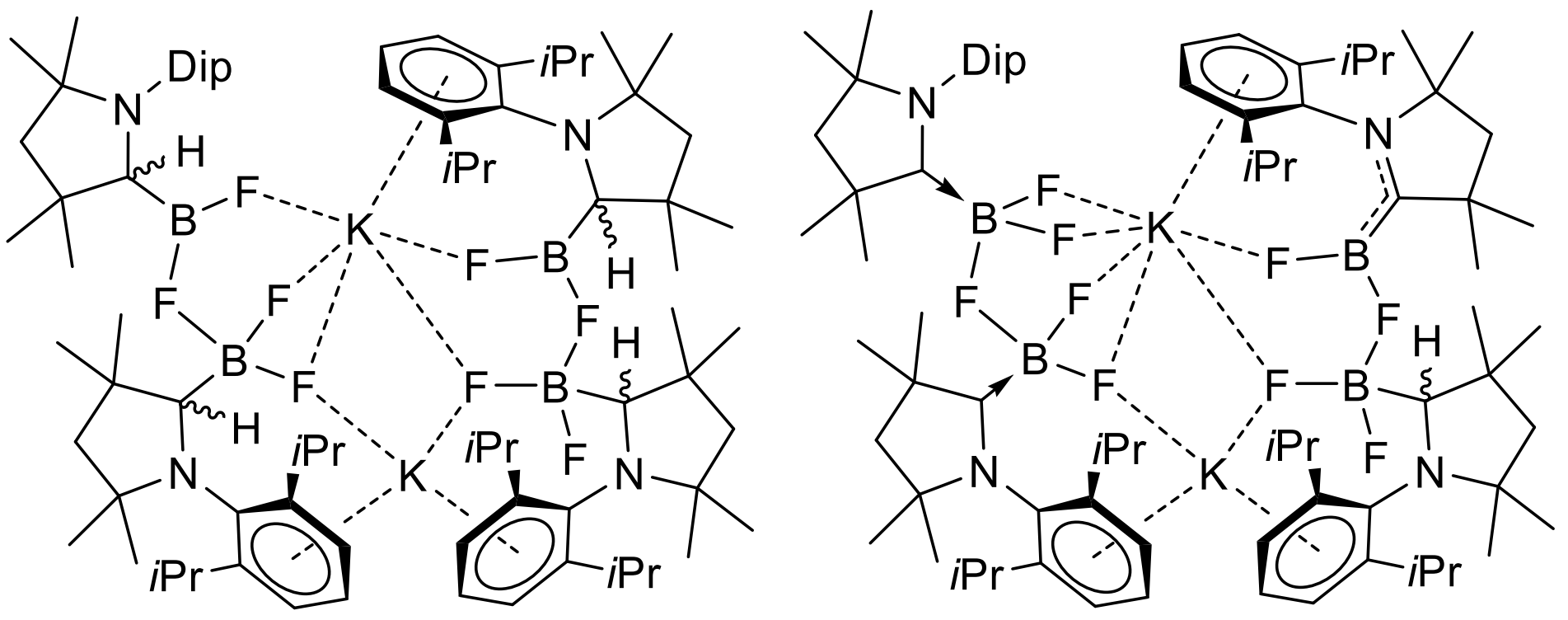

Compound **1** (487 mg, 1.38 mmol, 1.00 equiv.) and KC_8 (423 mg, 3.13 mmol, 2.27 equiv.) were dissolved in benzene. The mixture turned deep purple, and was stirred overnight at rt. After filtration and removal of all volatiles *in vacuo*, crude **6** was obtained as a pale pink powder (445 mg). Recrystallisation in pentane at –30 °C yielded a small crop of red crystals suitable for SCXRD analysis, the data of which refined as a 4:1 mixture of the tetranuclear complexes **6a** and **6b**. Various recrystallisations of **6** always provided the same set of ^{1}H, ^{11}B and ^{19}F NMR

resonances but in different relative compositions, suggesting the formation of multiple tetramers with varying compositions, thereby preventing its full characterisation. NMR data are provided for crude **6**. ^{1}H NMR (400.1 MHz, C_6D_6): δ = 6.96 (br, Dip-*H*), 5.08 (s, BC*H*), 4.85 (s, BC*H*), 4.20 (br, *i*Pr-C*H*), 4.05 (s, BC*H*), 3.40-3.75 (multiple overlapping br, *i*Pr-C*H*), 2.81-3.16 (multiple overlapping br, *i*Pr-C*H*), 1.70-1.99 (multiple overlapping m, CH_2), 0.98-1.61 (multiple overlapping br + s, *i*Pr-CH_3 + $C(CH_3)_2$), 0.83 (s, $C(CH_3)_2$), 0.73 (s, $C(CH_3)_2$) ppm. ^{11}B{^{1}H} NMR (128.4 MHz, C_6D_6): δ = 23-34 (v. br, BF_2), –1.2-0.0 (m, BF_3) ppm. ^{19}F NMR (376.6 MHz, C_6D_6): δ = −128.0 (br, B*F*B), –133.1 (br, BF_3K), –135.3 (br, BF_3K), –152.8 (br, BF_2) ppm.

Synthesis of 7^{Na}

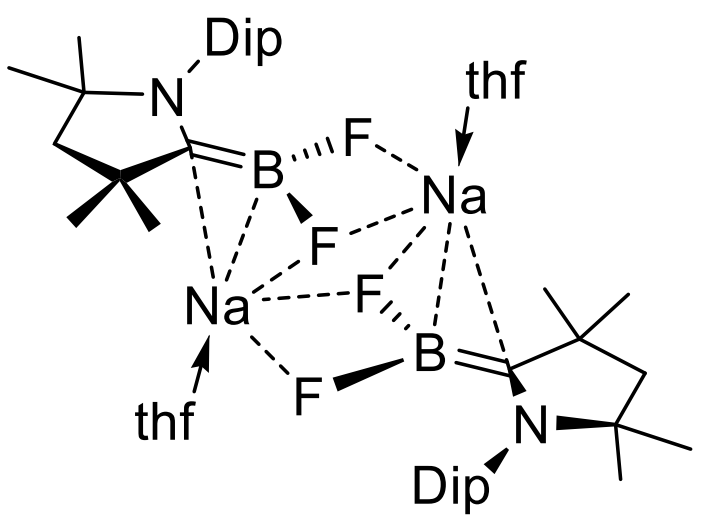

Compound **1** (0.20 g, 0.57 mmol, 1.0 equiv.) and sodium sand (32 mg, 1.4 mmol, 2.5 equiv.), and naphthalene (7.2 mg, 57 µmol, 0.10 equiv.) were dissolved in THF at rt. The mixture turned instantly purple, then, after a few minutes of vigorous stirring, dark red, and was left to stir overnight at rt. After removal of the solvent under reduced pressure, the dark red residue was extracted with hexane (1 mL) and the solution stored overnight at −30 °C, affording orange-red crystals of **7^{Na}** (0.12 g, 0.34 mmol, 59%). *Note: Since drying of isolated 7^{Na} always resulted in some decomposition, NMR data was acquired on a sample prepared directly in d_8-THF.* ^{1}H NMR (500.1 MHz, d_8-THF): δ = 6.82-6.71 (m, 3H, Dip-*H*), 3.78 (sept, ^{3}J = 6.9 Hz, 2H, *i*Pr-C*H*), 1.76 (s, 2H, CH_2), 1.33 (s, 6H, $NC(CH_3)_2$), 1.12 (d, ^{3}J = 6.9 Hz, 6H, *i*Pr-CH_3), 1.10 (s, 6H, $CC(CH_3)_2$), 1.09 (d, ^{3}J = 6.9 Hz, 6H, *i*Pr-CH_3) ppm. ^{13}C{^{1}H} NMR (125.8 MHz, d_8-THF): δ = 150.6 (*o*-Dip-*C*), 145.9 (*i*-Dip-*C*), 122.3 (*m*-Dip-*C*), 121.9 (*p*-Dip-*C*), 61.5 (d, $^{4}J_{C\text{-}F}$ = 2.0 Hz, CH_2), 61.1 (CMe_2), 40.4 (dd, $^{3}J_{C\text{-}F}$ = 5.2 Hz, $^{3}J_{C\text{-}F}$ = 7.7 Hz, $NCMe_2$), 33.1 ($NC(CH_3)_2$), 27.9 ($CC(CH_3)_2$), 27.0 (*i*Pr-*C*H), 24.8 (*i*Pr-CH_3), 24.1 (*i*Pr-CH_3) ppm. *Note: due to excessive broadening the carbene carbon resonance could not be detected by HMBC.* ^{11}B NMR (160.5 MHz, d_8-THF): δ = 27.7 (br s, $\omega_{1/2} \approx$ 330 Hz, BF_2) ppm. ^{19}F{^{11}B} NMR (470.6 MHz, d_8-THF): δ = –156.6 (d, $^{2}J_{19F\text{-}19F}$ = 32.3 Hz, 1F, $BFNa_2$), –159.7 (d, $^{2}J_{19F\text{-}19F}$ = 32.3 Hz, 1F, B*F*Na) ppm. ^{23}Na NMR (132.3 MHz, d_8-THF): δ = 6.6 (br s, $\omega_{1/2} \approx$ 310 Hz) ppm. *Note: compound 7^{Na} proved too sensitive for further characterisation by HRMS or elemental analysis.*

Disproportionation of 7^{Na} in benzene

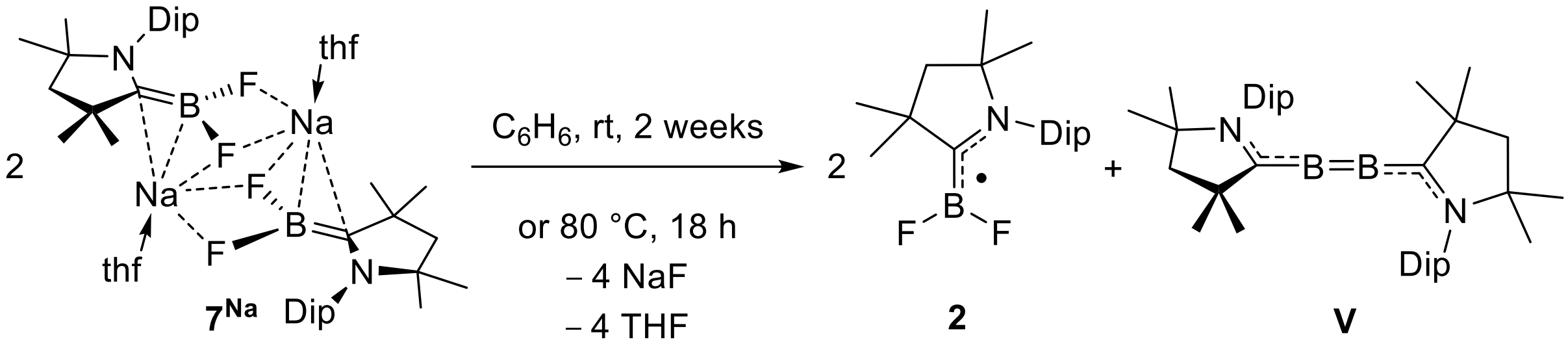

Isolated 7^{Na} (20.0 mg, 28.0 µmol) was dissolved in benzene (0.5 mL) and the red solution left undisturbed at rt for two weeks, during which it turned purple and purple crystals of **V**[4] formed (identified by ^{11}B NMR spectroscopy and SCXRD). An EPR spectrum of the solution showed the presence of large quantities of radical **2**. Full disproportionation of 7^{Na} to **2** and **V** could be achieved by heating 7^{Na} in benzene at 80 °C overnight. ^{1}H NMR (400.1 MHz, C_6D_6): δ = 7.06-6.98 (m, 6H, Dip-*H*), 3.40 (sept, ^{3}J = 6.8 Hz, 2H, *i*Pr-C*H*), 3.08 (sept, ^{3}J = 6.8 Hz, 2H, *i*Pr-C*H*), 2.09 (s, 4H, CH_2), 1.87 (s, 6H C(CH_3)$_2$), 1.66 (s, 6H C(CH_3)$_2$), 1.49 (d, ^{3}J = 6.8 Hz, 6H, *i*Pr-CH_3), 1.28 (d, ^{3}J = 6.8 Hz, 6H, *i*Pr-CH_3), 1.21 (d, ^{3}J = 6.8 Hz, 6H, *i*Pr-CH_3), 1.20 (s, 6H C(CH_3)$_2$), 1.02 (s, 6H C(CH_3)$_2$), 0.68 (d, ^{3}J = 6.8 Hz, 6H, *i*Pr-CH_3) ppm. ^{11}B NMR (128.4 MHz, C_6D_6): δ = 81 ppm (**V**). *Note: these data are in agreement with those reported in the literature.*[4]

Direct synthesis of V from 1

1 (50.0 mg, 142 µmol, 2.00 equiv) was dissolved in diethyl ether (1 mL) and Li sand in NaF (10.0 wt%, 29.5 mg, 425 µmol, 2.50 equiv.) was added. The reaction mixture, which turned rapidly purple, was stirred for 24 at rt prior to filtration and removal of volatiles from the filtrate *in vacuo*. The purple residue was extracted with *n*-hexane (3×0.5 mL) and recrystallised at rt, yielding **V** as purple crystals (14.8 mg, 25.0 µmol, 35%).

***In-situ* synthesis of 7^{Li} in THF**

1 (25.0 mg, 70.8 µmol, 1.00 equiv.) was dissolved in THF (1 mL) and Li sand in NaF (10.0 wt%, 19.6 mg, 283 µmol, 4.00 equiv.) added. The reaction mixture was stirred at rt for 24 h prior to filtration, yielding a clean, dark-red solution of 7^{Li} in THF to be used for further reactivity. The similarity of the ^{19}F NMR spectrum with 7^{Na} suggests a similar dimeric structure. ^{11}B NMR (160.5 MHz, d_8-THF): δ = 28.2 (br s, $\omega_{1/2}$ ≈ 280 Hz) ppm. $^{19}F\{^{11}B\}$ NMR (470.6 MHz, d_8-THF): δ = –157.1 (br, B*F*Li_2), –159.1 (br, B*F*Li_2) ppm.

***In-situ* synthesis of 7^{K} in Et_2O**

1 (25.0 mg, 70.8 µmol, 1.00 equiv.) was dissolved in diethyl ether (1 mL) and KC_8 (38.3 mg, 283 µmol, 4.00 equiv.) added. The reaction mixture was stirred at rt for 24 h prior to filtration, yielding a clean, dark-red solution of $\mathbf{7^{K}}$ in Et_2O to be used for further reactivity. The presence of a single broad resonance in the ^{19}F NMR spectrum significantly upfield from $\mathbf{7^{Li/Na}}$ suggests a different structure. ^{11}B NMR (160.5 MHz, d_8-THF): δ = 28.5 (br s, $\omega_{1/2}$ ≈ 260 Hz) ppm. $^{19}F\{^{11}B\}$ NMR (470.6 MHz, d_8-THF): δ = –152.1 (br m, $\omega_{1/2}$ ≈ 230 Hz) ppm.

Synthesis of 8^{nPr}

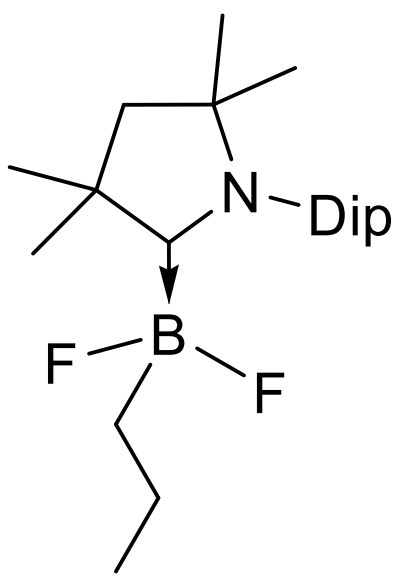

1 (25.0 mg, 70.8 µmol, 1.00 equiv.) was dissolved in diethyl ether (1 mL) and KC_8 (38.3 mg, 283 µmol, 4.00 equiv.) added. The reaction mixture was stirred at rt for 24 h. The now dark-red suspension was filtered and 1-iodopropane (50.0 µL, 515 µmol, 7.27 equiv.) added. The mixture instantly turned pale yellow. After removal of volatiles *in vacuo* the solid residue was washed with *n*-hexane (2×0.5 mL) and extracted with benzene (2×0.5 mL). Removal of the solvent in vacuo yielded $\mathbf{8^{\textit{n}Pr}}$ as a colourless solid (18.9 mg, 50.1 µmol, 71%). Crystals suitable for SCXRD analysis were obtained from a saturated hexane solution at –30 °C. ^{1}H NMR (500.1 MHz, C_6D_6): δ = 7.13-7.10 (m, 1H, *p*-Dip-*H*), 7.02-7.00 (m, 2H, *m*-Dip-*H*), 2.66 (sept, 2H, ^{3}J = 6.6 Hz, *i*Pr-C*H*), 1.97-1.88 (m, 2H, *n*Pr-CH_2Me), 1.47 (s, 6H, $C(CH_3)_2$), 1.43 (d, 6H, ^{3}J = 6.6 Hz, *i*Pr-CH_3), 1.38 (s, 2H, CH_2), 1.30 (t, 3H, ^{3}J = 7.4 Hz, *n*Pr-CH_3), 1.14 (d, 6H, ^{3}J = 6.6 Hz, *i*Pr-CH_3), 0.78 (s, 6H, $NC(CH_3)_2$), 0.76–0.65 (m, 2H, BCH_2) ppm. $^{13}C\{^{1}H\}$ NMR (125.8 MHz, C_6D_6): δ = 228.7 (BC_{CAAC}, determined by HMBC), 144.8 (*o*-Dip-*C*), 133.6 (*i*-Dip-*C*), 129.3 (*p*-Dip-*C*), 124.5 (*m*-Dip-*C*), 78.9 ($NCMe_2$), 52.2 ($CCMe_2$), 51.3 (CH_2), 29.7 (*i*Pr-*C*H), 28.8 ($C(CH_3)_2$), 28.3 ($NC(CH_3)_2$), 27.8 (br, BCH_2, determined by HMBC), 26.3 (*i*Pr-CH_3), 23.5 (*i*Pr-CH_3), 18.9 (*n*Pr-CH_3), 18.3 (*n*Pr-CH_2Me) ppm. ^{11}B NMR (160.5 MHz, C_6D_6): δ = 5.8 (br) ppm. ^{19}F NMR (470.6 MHz, C_6D_6): δ = −153.6 (br) ppm. HRMS (LIFDI-pos., toluene) for $[C_{23}H_{38}BFN]^{+}$ = $[M – F]^{+}$: *m/z* calcd. = 358.3076; found = 358.3065.

Synthesis of 8^{iPr}

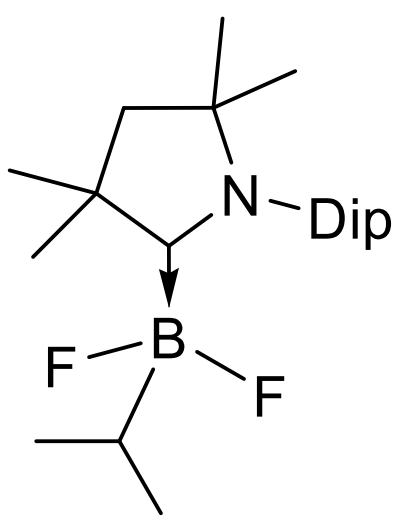

1 (25.0 mg, 70.8 µmol, 1.00 equiv.) was dissolved in diethyl ether (1 mL) and KC_8 (38.3 mg, 283 µmol, 4.00 equiv.) added. The reaction mixture was stirred at rt for 24 h. The now dark-red suspension was filtered and 1-iodopropane (50.0 µL, 500 µmol, 7.07 equiv.) added. The mixture instantly turned pale yellow. After removal of volatiles *in vacuo* the solid residue was washed

with *n*-hexane (2×0.5 mL) and extracted with benzene (2×0.5 mL). Removal of the solvent in vacuo yielded **8$^{\textbf{\textit{i}Pr}}$** as a colourless solid (21.5 mg, 57.0 µmol, 81%). Crystals suitable for SCXRD analysis were obtained from a saturated hexane solution at –30 °C. ^{1}H NMR (500.1 MHz, C_6D_6): δ = 7.13-7.10 (m, 1H, *p*-Dip-*H*), 7.03-7.00 (m, 2H, *m*-Dip-*H*), 2.70 (sept, 2H, ^{3}J = 6.6 Hz, Dip-*i*Pr-C*H*), 1.45 (d, 6H, ^{3}J = 6.6 Hz, *i*Pr-C*H₃*), 1.43 (s, 6H, C(C*H₃*)₂), 1.43 (d, 6H, ^{3}J = 6.6 Hz, *i*Pr-C*H₃*), 1.39 (s, 2H, C*H₂*), 1.32 (d, 6H, ^{3}J = 7.0 Hz, B*i*Pr-C*H₃*), 1.20-1.12 (m, 1H, B*i*Pr-C*H*), 1.43 (d, 6H, ^{3}J = 6.6 Hz, *i*Pr-C*H₃*), 1.39 (s, 2H, C*H₂*), 1.32 (br m, 1H, B*i*Pr-C*H₃*), 1.15 (d, 6H, ^{3}J = 6.6 Hz, *i*Pr-C*H₃*), 0.78 (s, 6H, NC(C*H₃*)₂) ppm. ^{13}C{^{1}H} NMR (125.8 MHz, C_6D_6): δ = 229.9 (BC_{CAAC}, determined by HMBC), 144.7 (*o*-Dip-*C*), 133.9 (*i*-Dip-*C*), 129.1 (*p*-Dip-*C*), 124.6 (*m*-Dip-*C*), 79.2 (N*C*Me₂), 52.0 (C*C*Me₂), 52.0 (*C*H₂), 29.5 (*i*Pr-*C*H), 28.7 (C(*C*H₃)₂), 28.6 (NC(*C*H₃)₂), 27.3 (*i*Pr-*C*H₃), 23.9 (*i*Pr-*C*H₃), 19.5 (*i*Pr-*C*H₃) ppm. *Note: the BiPr-*CH *resonance could not be detected even by HMBC due to quadrupolar broadening induced by the neighbouring boron nucleus.* ^{11}B NMR (160.5 MHz, C_6D_6): δ = 6.0 (br) ppm. ^{19}F NMR (470.6 MHz, C_6D_6): δ = −156.5 (br) ppm. HRMS (LIFDI-pos., toluene) for $[C_{23}H_{38}BFN]^{+}$ = $[M – F]^{+}$: *m/z* calcd. = 358.3076; found = 358.3064.

NMR spectra

Isolated compounds

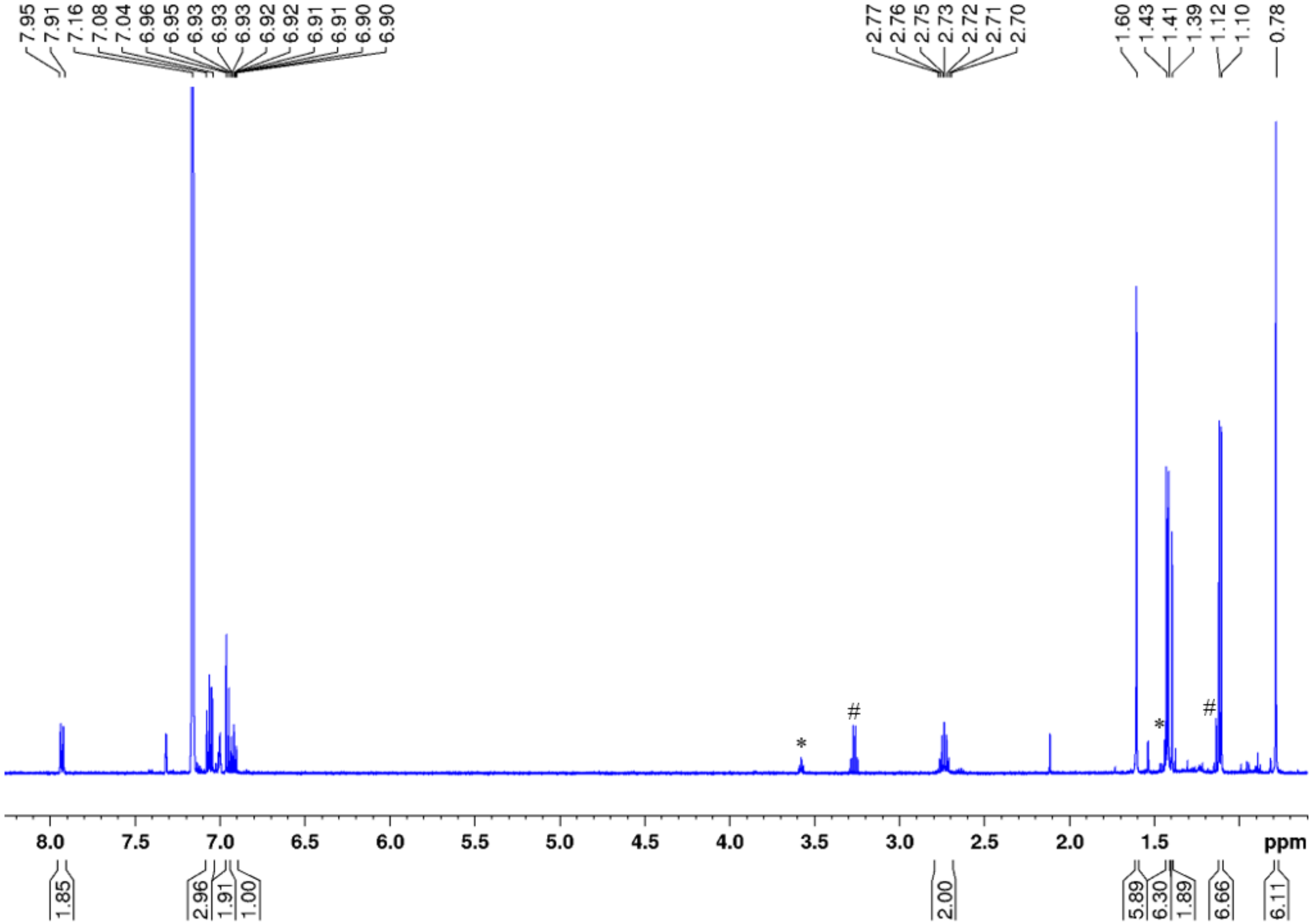

Figure S1. ^{1}H NMR spectrum of **3** in C_6D_6 (contaminated with ca. 5% of **1**). Residual THF and Et_2O resonances marked * and #, respectively.

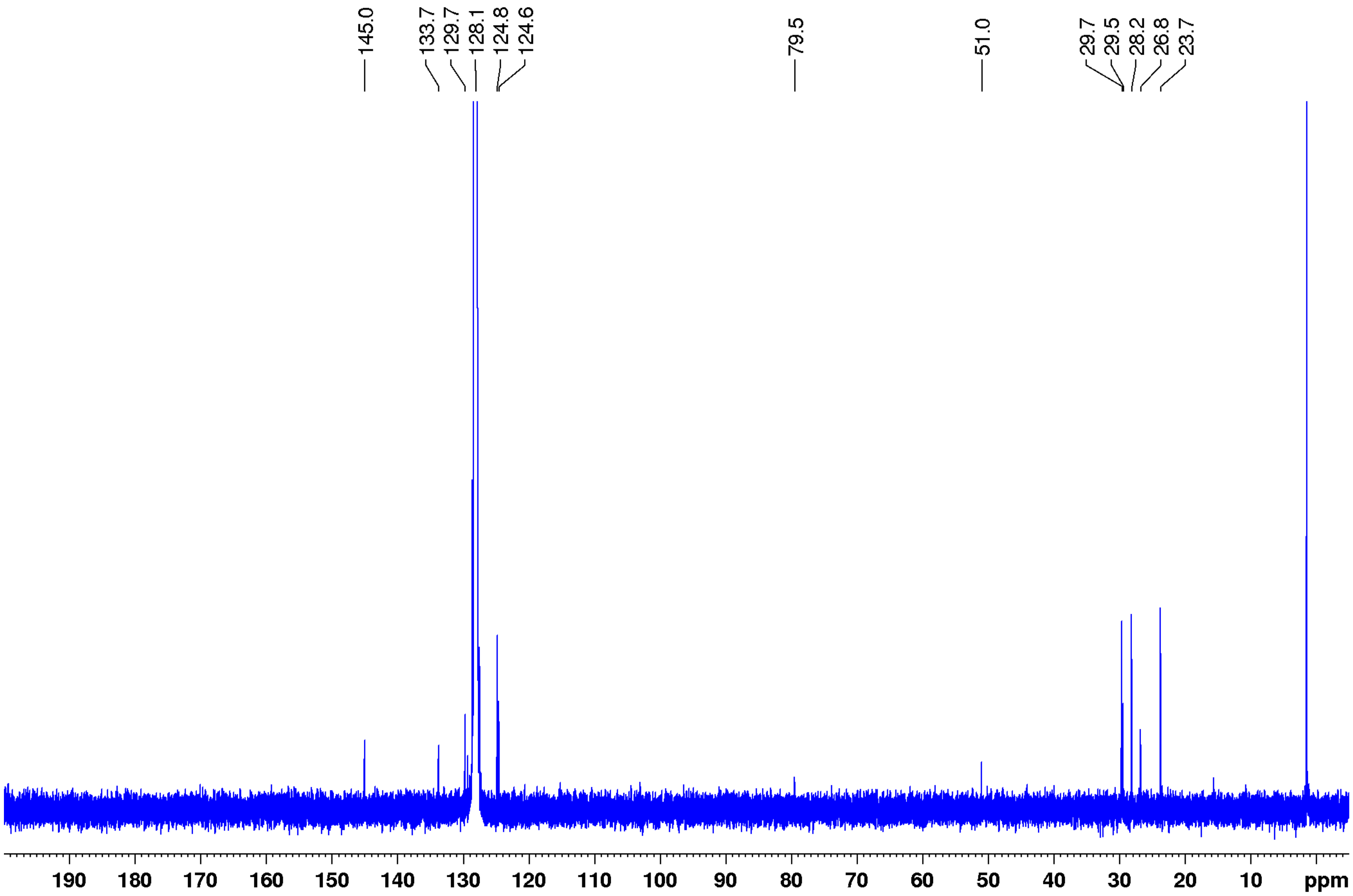

Figure S2. $^{13}C\{^1H\}$ NMR spectrum of **3** in C_6D_6.

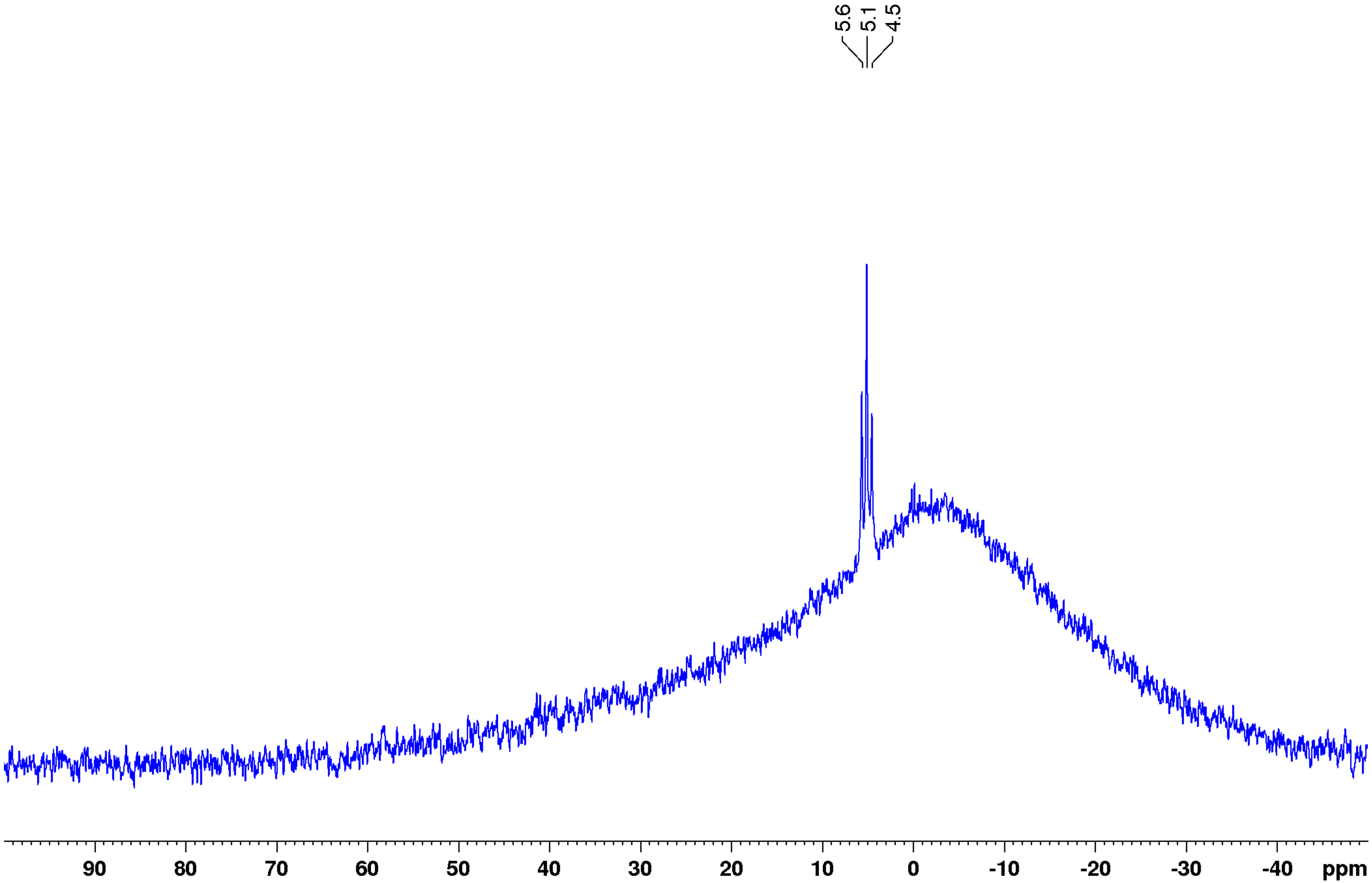

Figure S3. ^{11}B NMR spectrum of **3** in C_6D_6 (contaminated with ca. 5% of **1**).

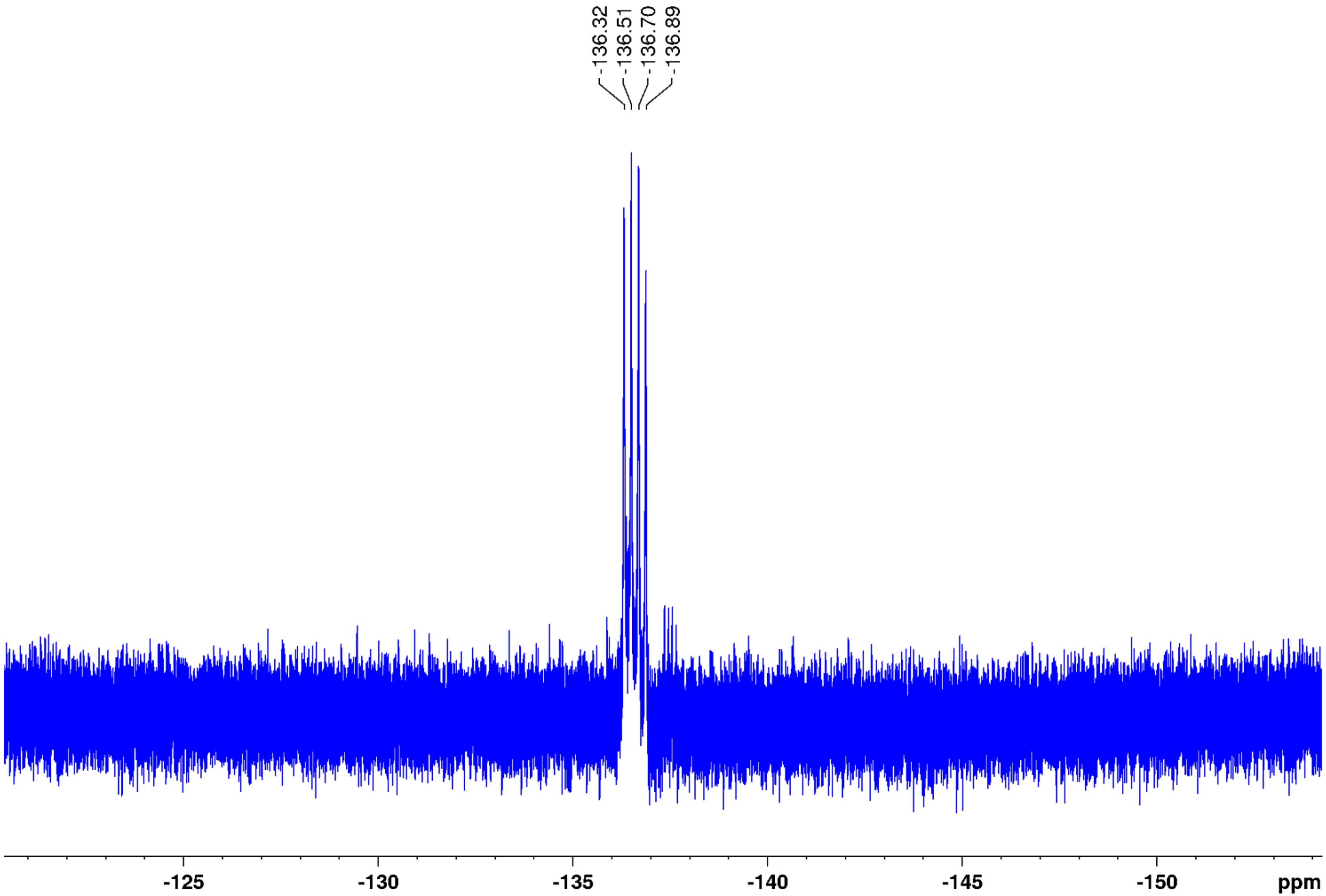

Figure S4. ^{19}F NMR spectrum of **3** in C_6D_6 (contaminated with ca. 5% of **1**).

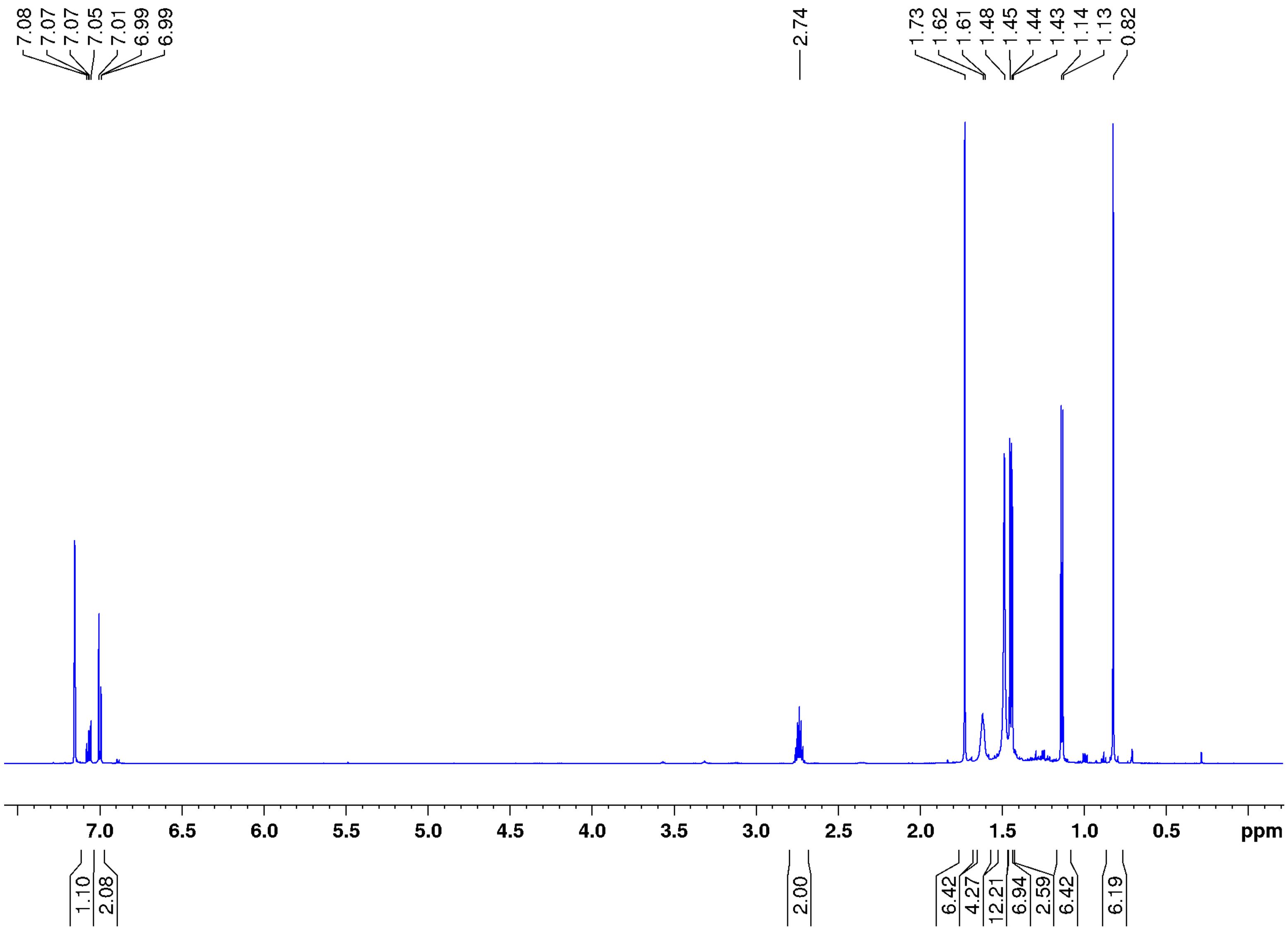

Figure S5. ^{1}H NMR spectrum of **4** in C_6D_6 (contaminated with ca. 1% of **1**).

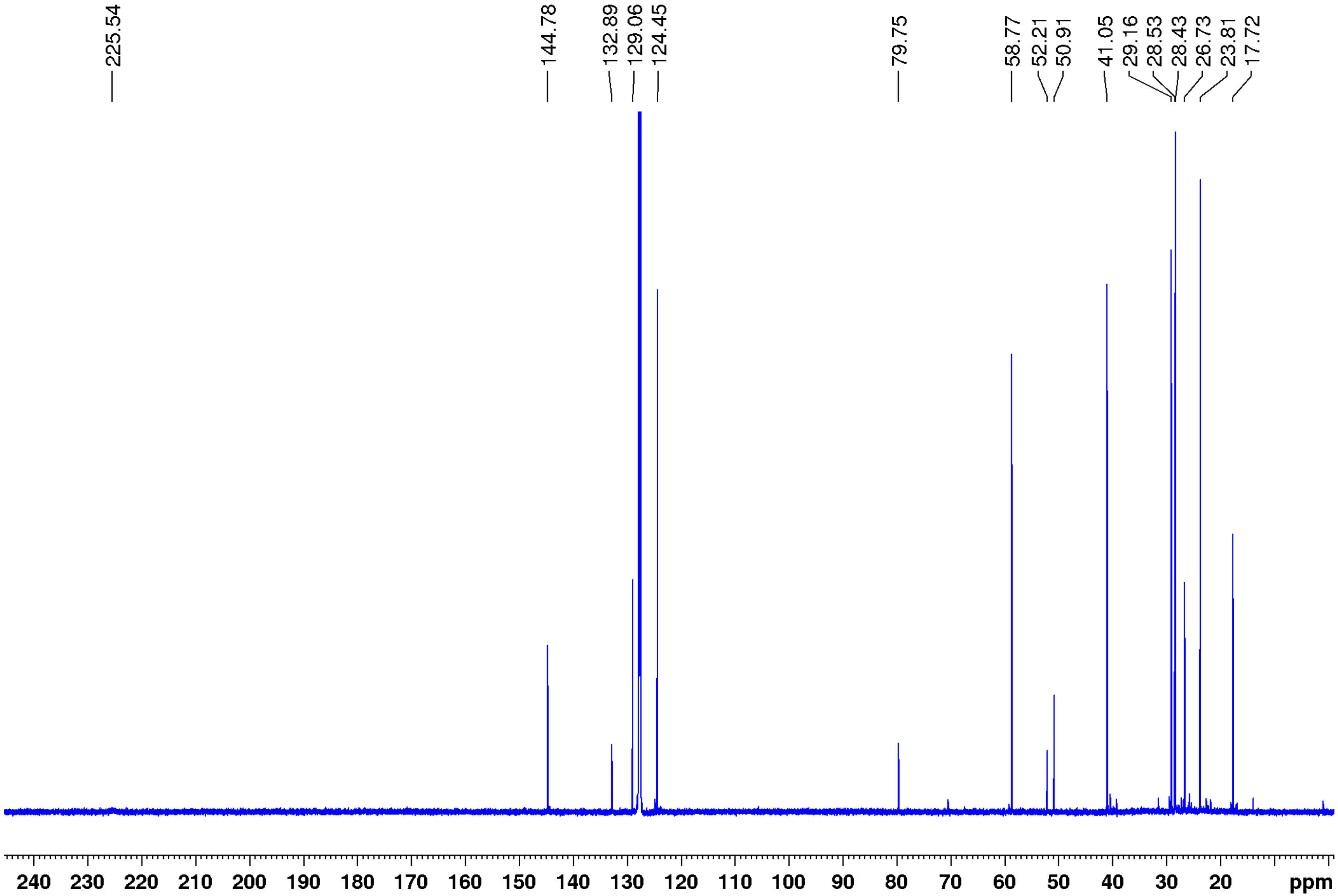

Figure S6. $^{13}C\{^1H\}$ NMR spectrum of **4** in C_6D_6 (contaminated with ca. 1% of **1**).

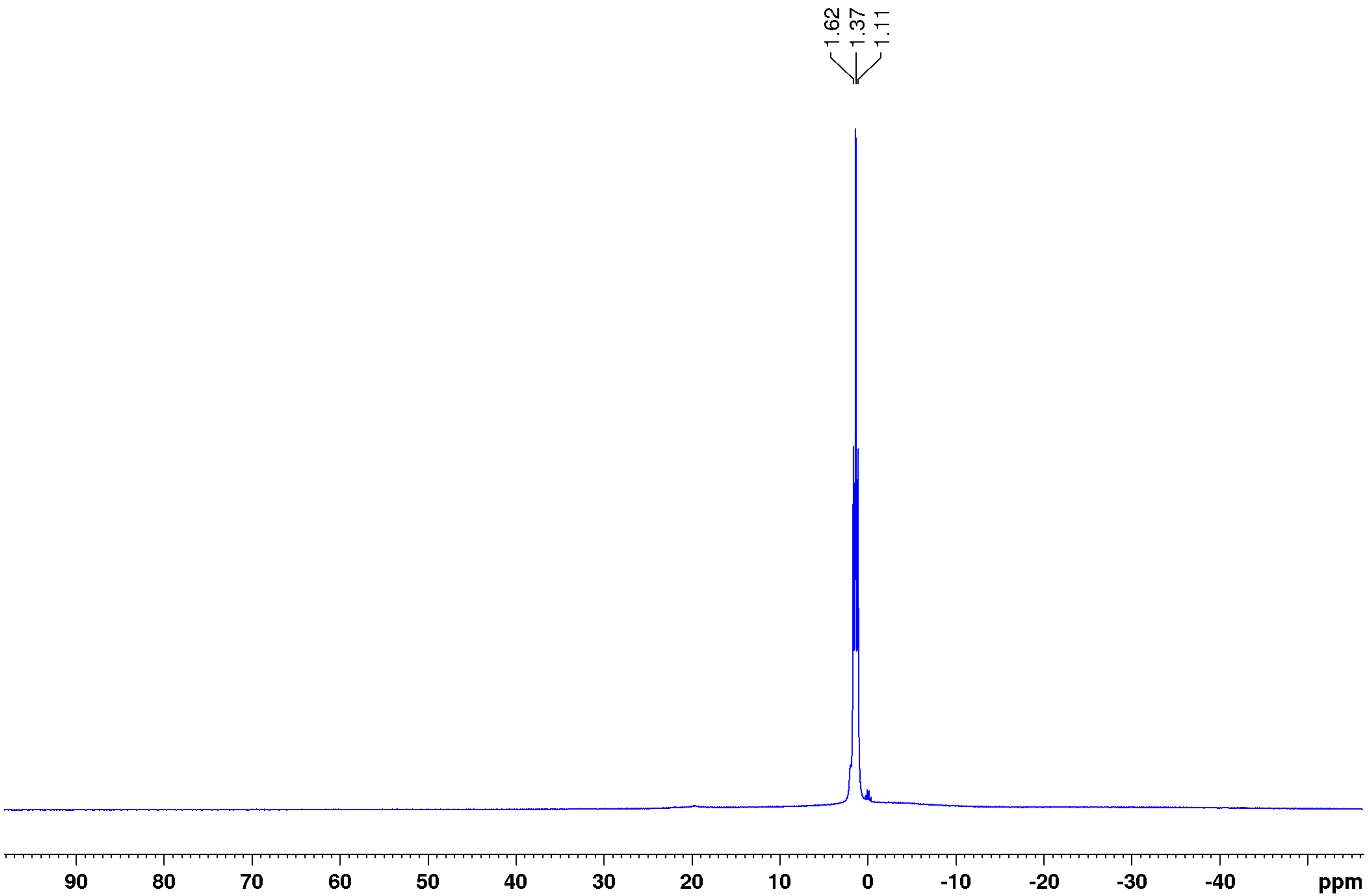

Figure S7. ^{11}B NMR spectrum of **4** in C_6D_6 (contaminated with ca. 1% of **1**).

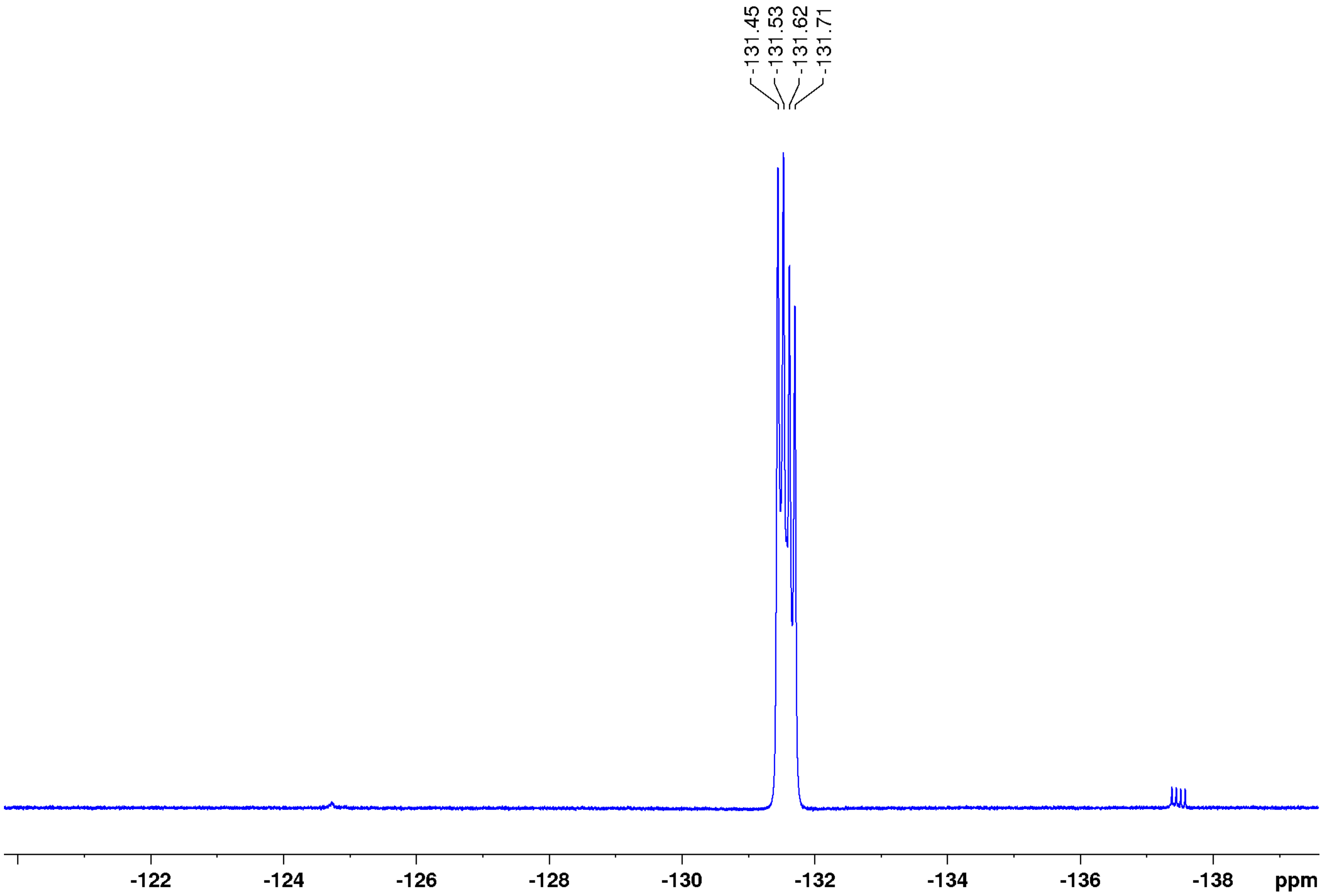

Figure S8. ^{19}F NMR spectrum of **4** in C_6D_6 (contaminated with ca. 1% of **1**).

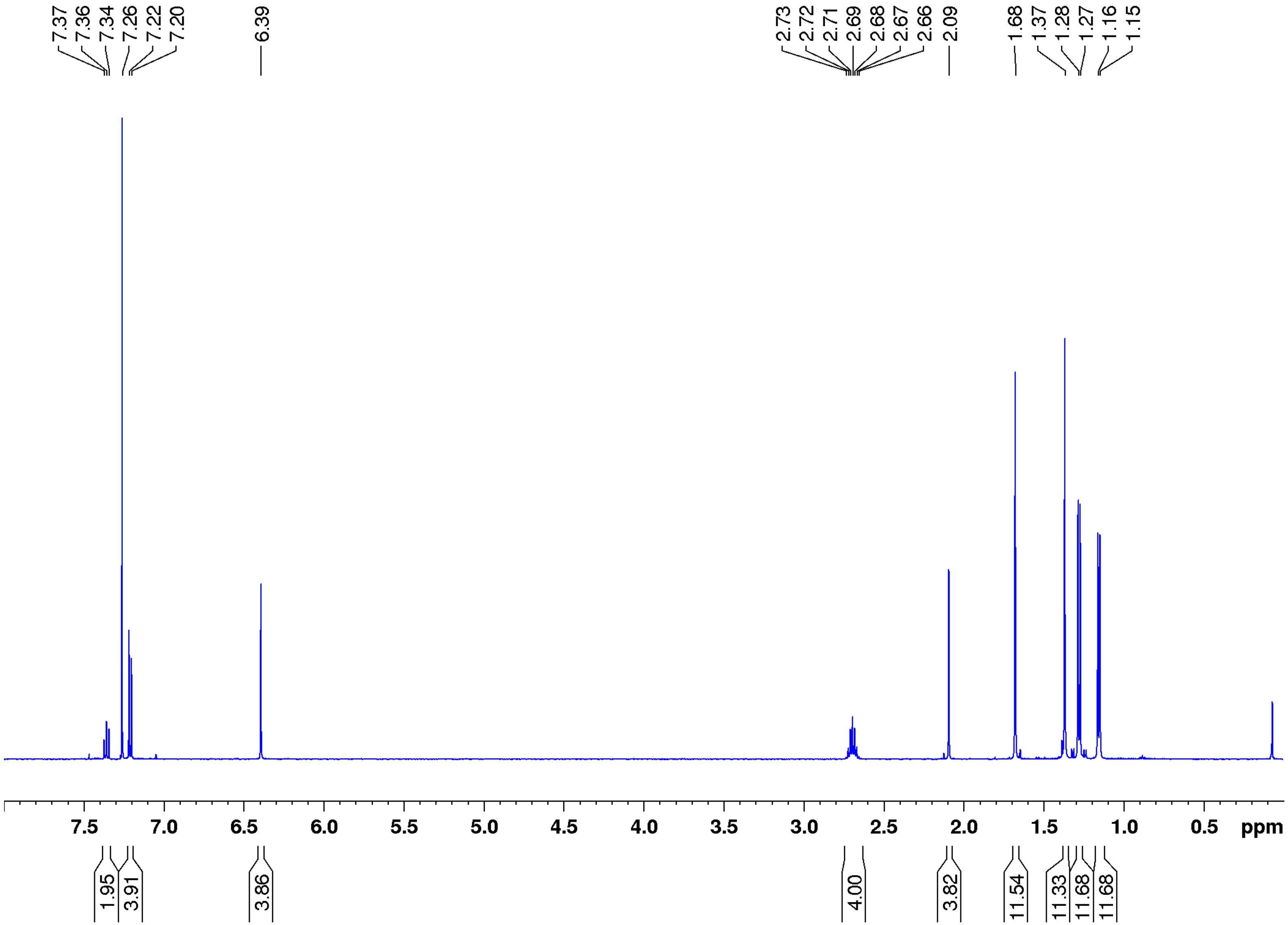

Figure S9. ^{1}H NMR spectrum of **5** in $CDCl_3$ (contaminated with ca. 3% of **1**).

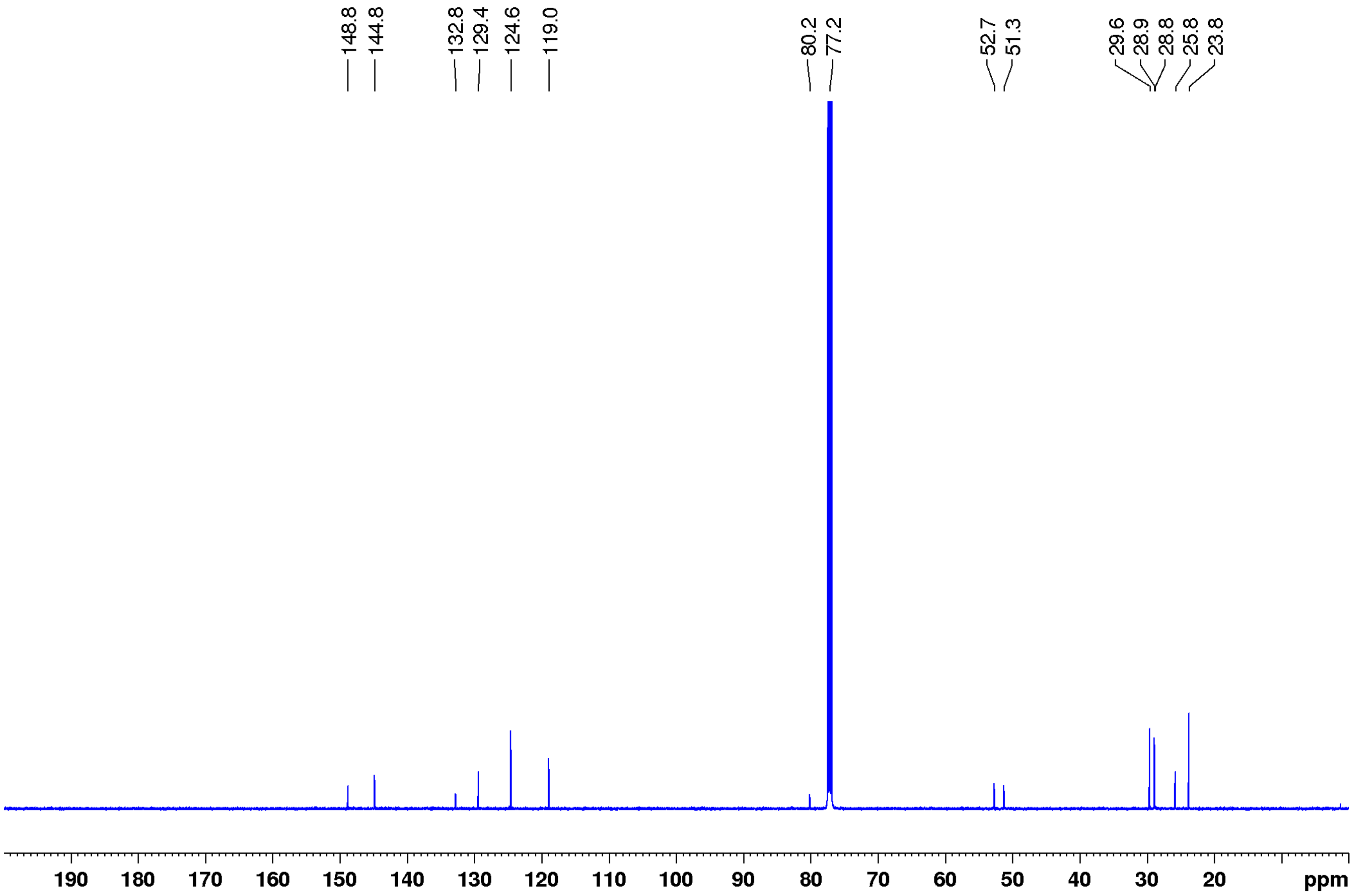

Figure S10. $^{13}C\{^1H\}$ NMR spectrum of **5** in $CDCl_3$.

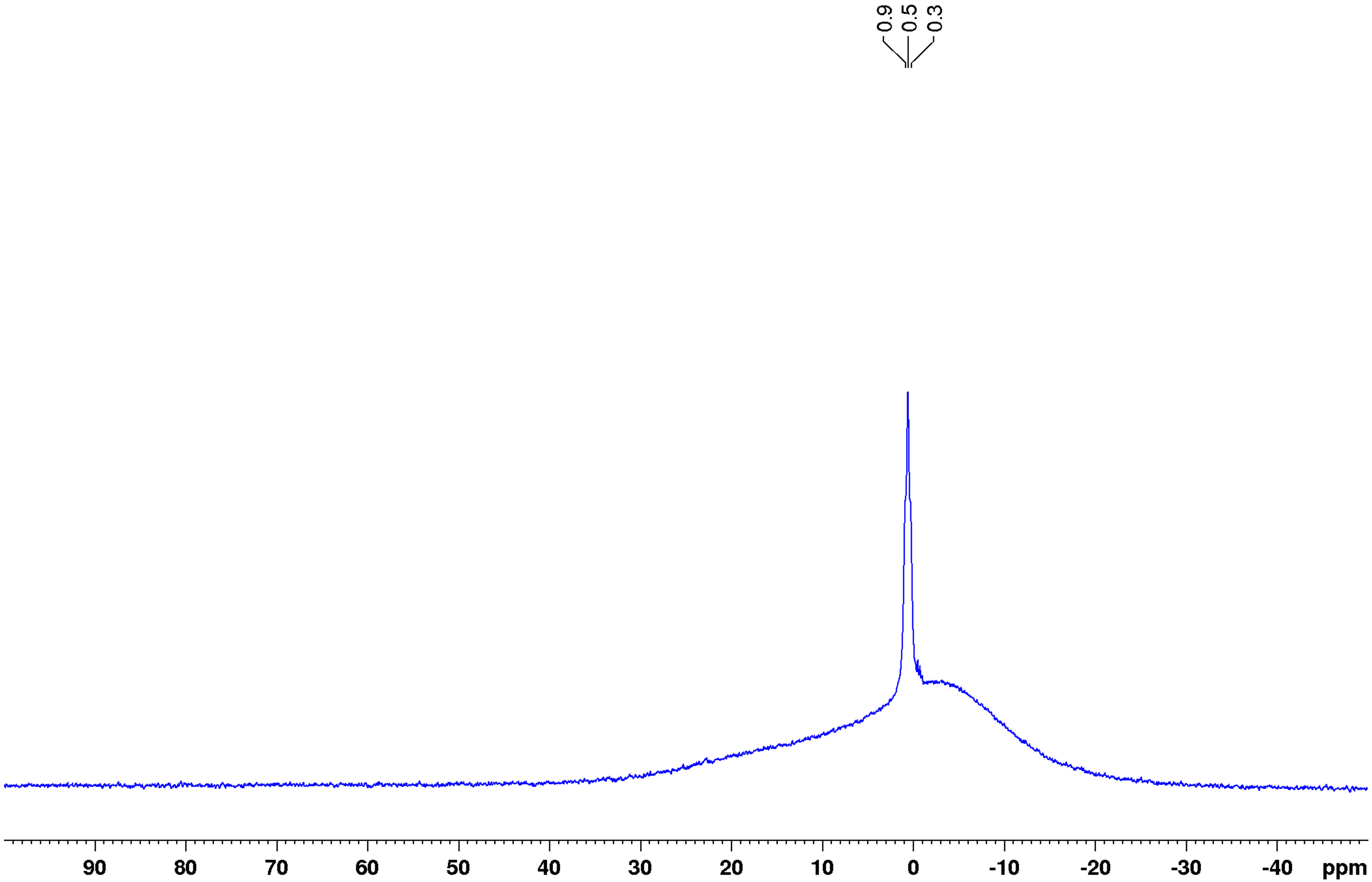

Figure S11. ^{11}B NMR spectrum of **5** in $CDCl_3$ (contaminated with ca. 3% of **1**).

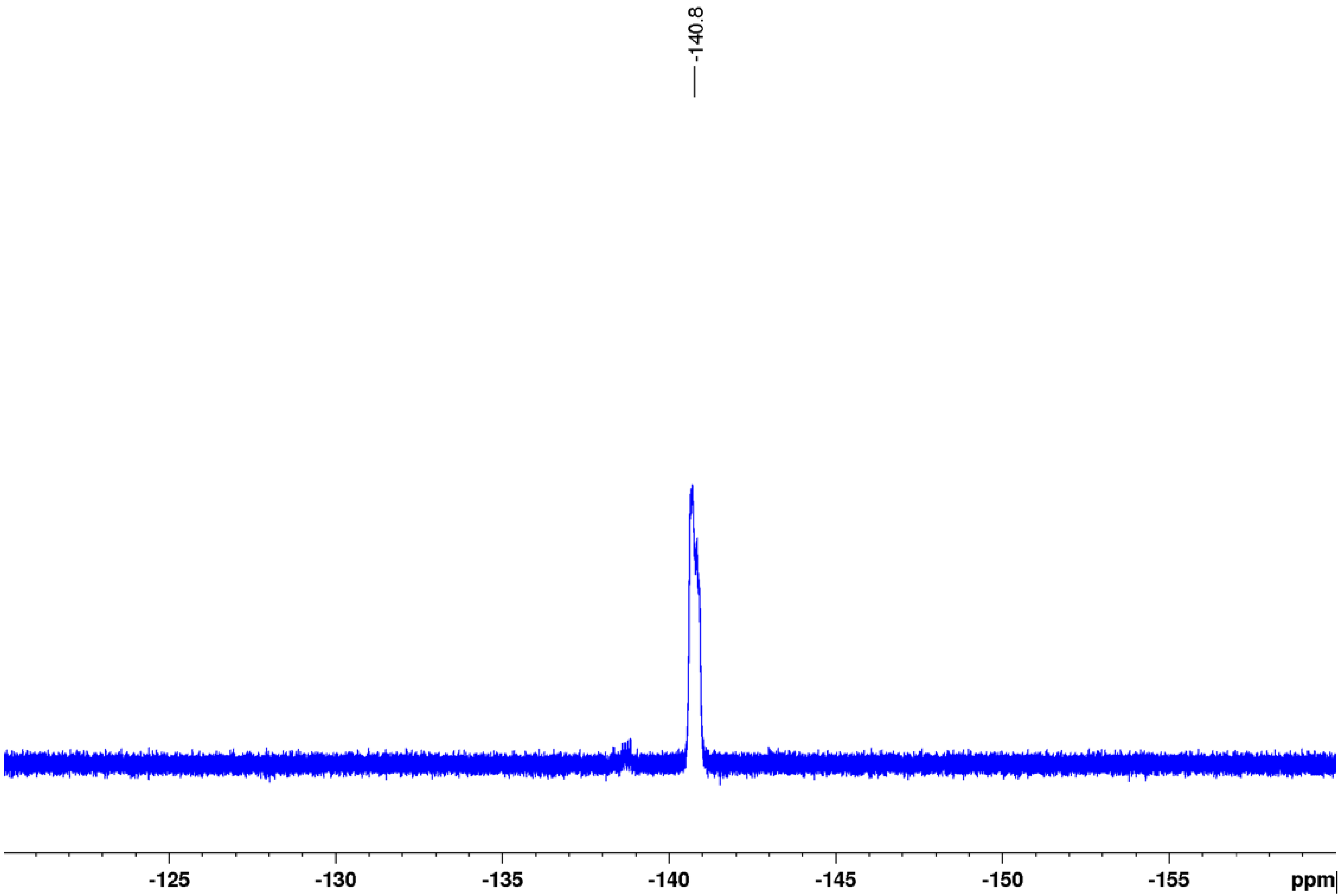

Figure S12. ^{19}F NMR spectrum of **5** in $CDCl_3$ (contaminated with ca. 3% of **1**).

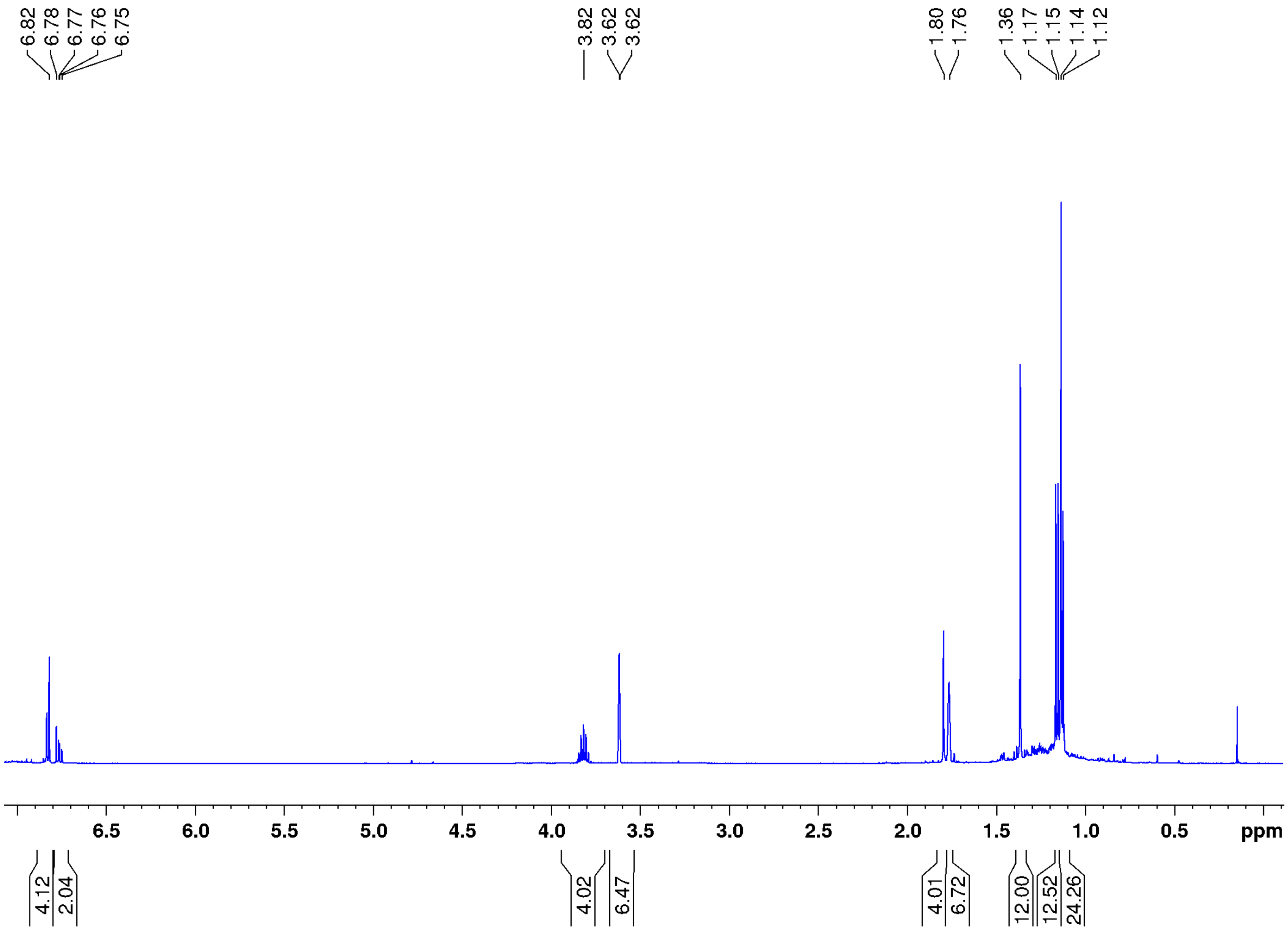

Figure S13. ^{1}H NMR spectrum of $\mathbf{7^{Na}}$ in d_8-THF.

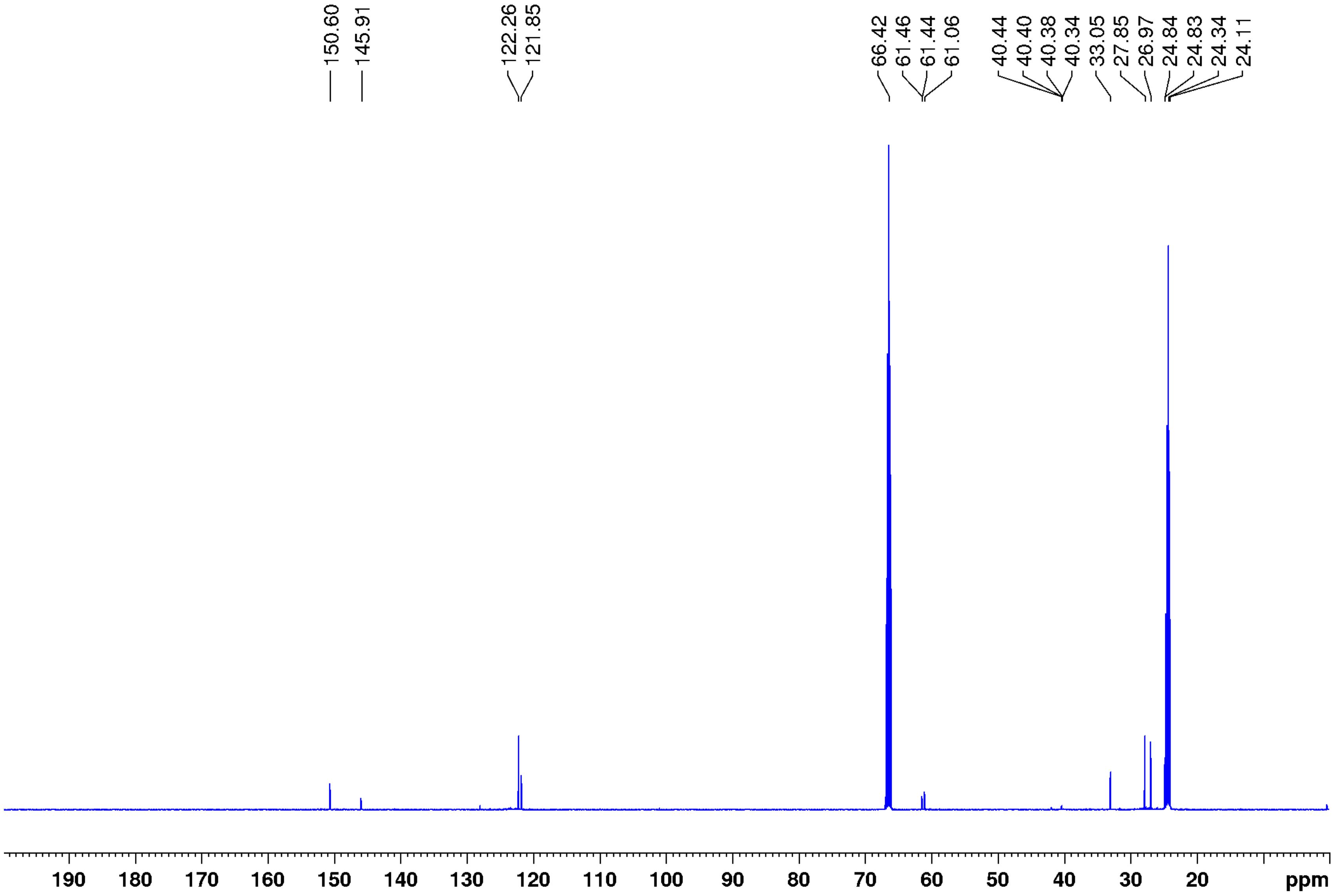

Figure S14. $^{13}C\{^{1}H\}$ NMR spectrum of $\mathbf{7^{Na}}$ in d_8-THF.

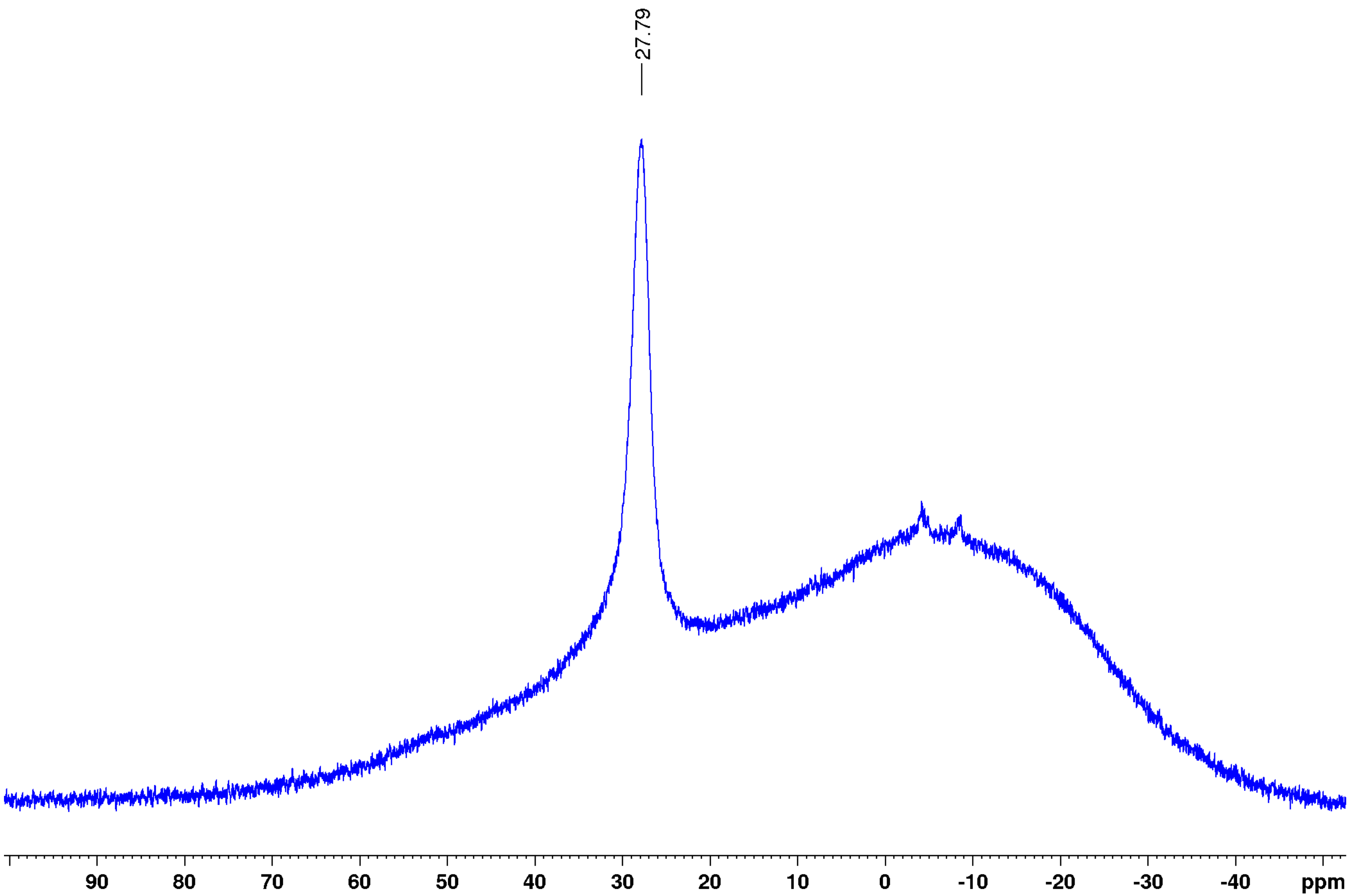

Figure S15. ^{11}B NMR spectrum of $\mathbf{7^{Na}}$ in d_8-THF.

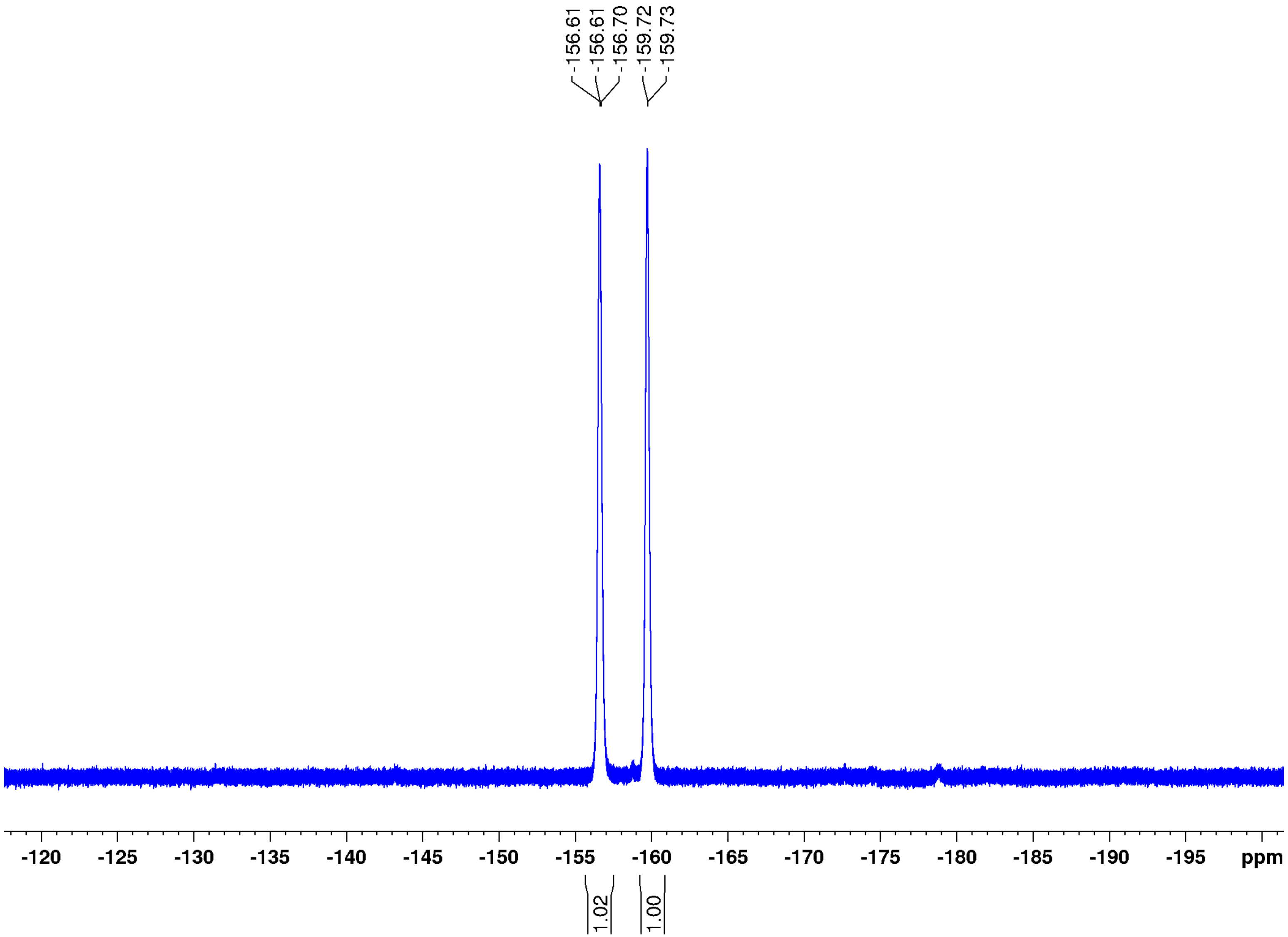

Figure S16. ^{19}F NMR spectrum of $\mathbf{7}^{\mathbf{Na}}$ in d_8-THF.

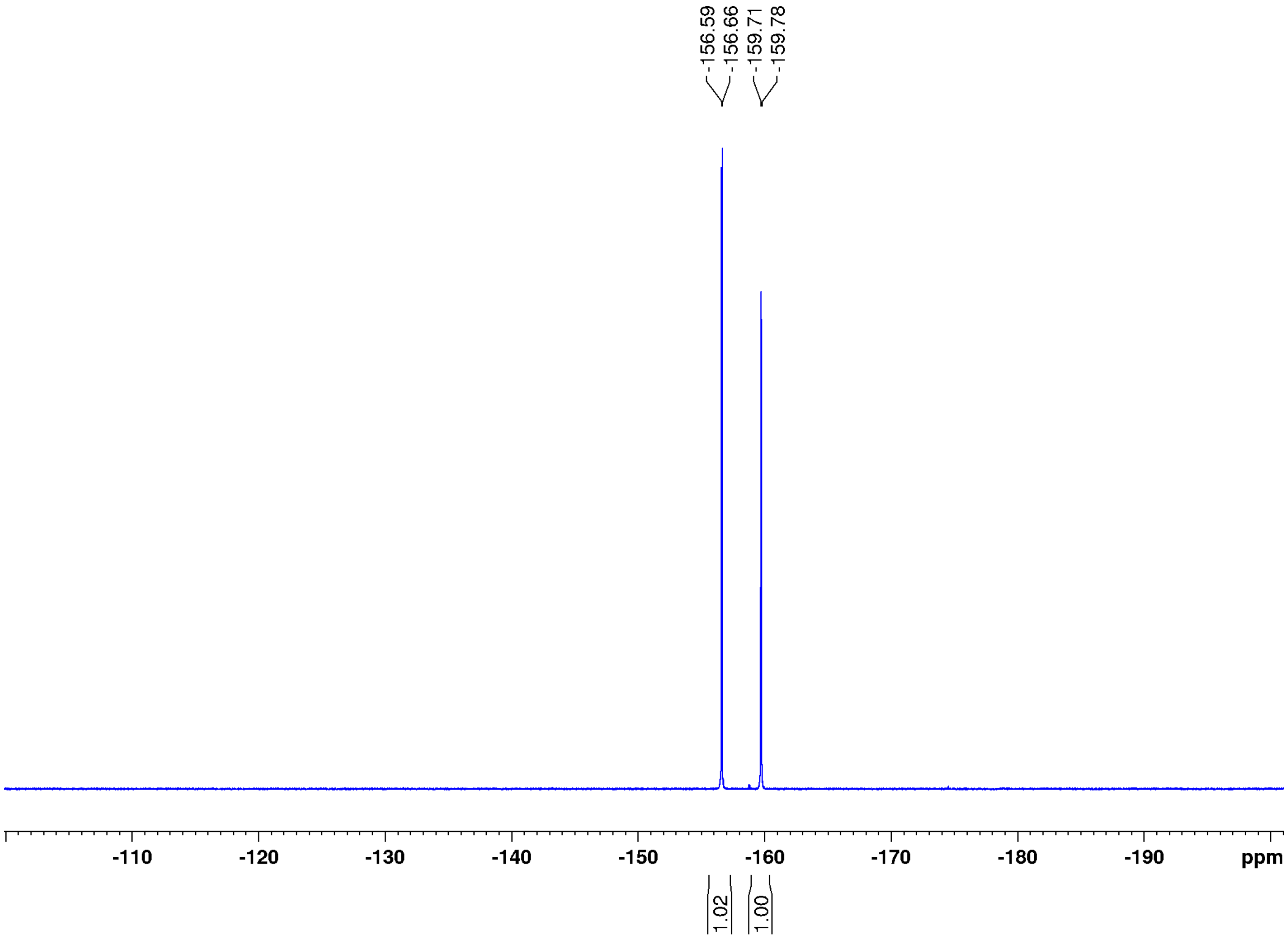

Figure S17. $^{19}F\{^{11}B\}$ NMR spectrum of $\mathbf{7^{Na}}$ in d_8-THF.

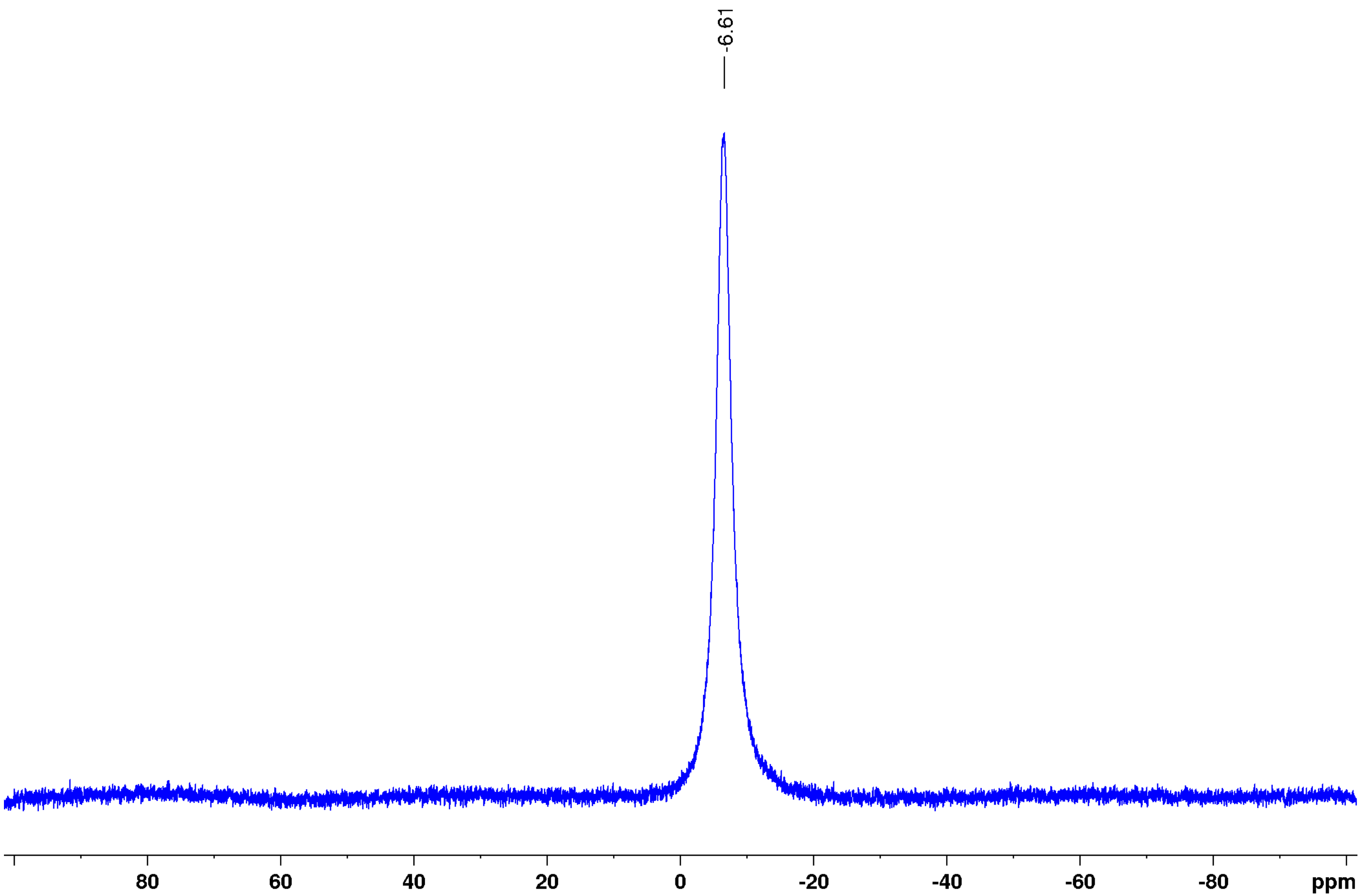

Figure S18. ^{27}Na NMR spectrum of $\mathbf{7^{Na}}$ in d_8-THF.

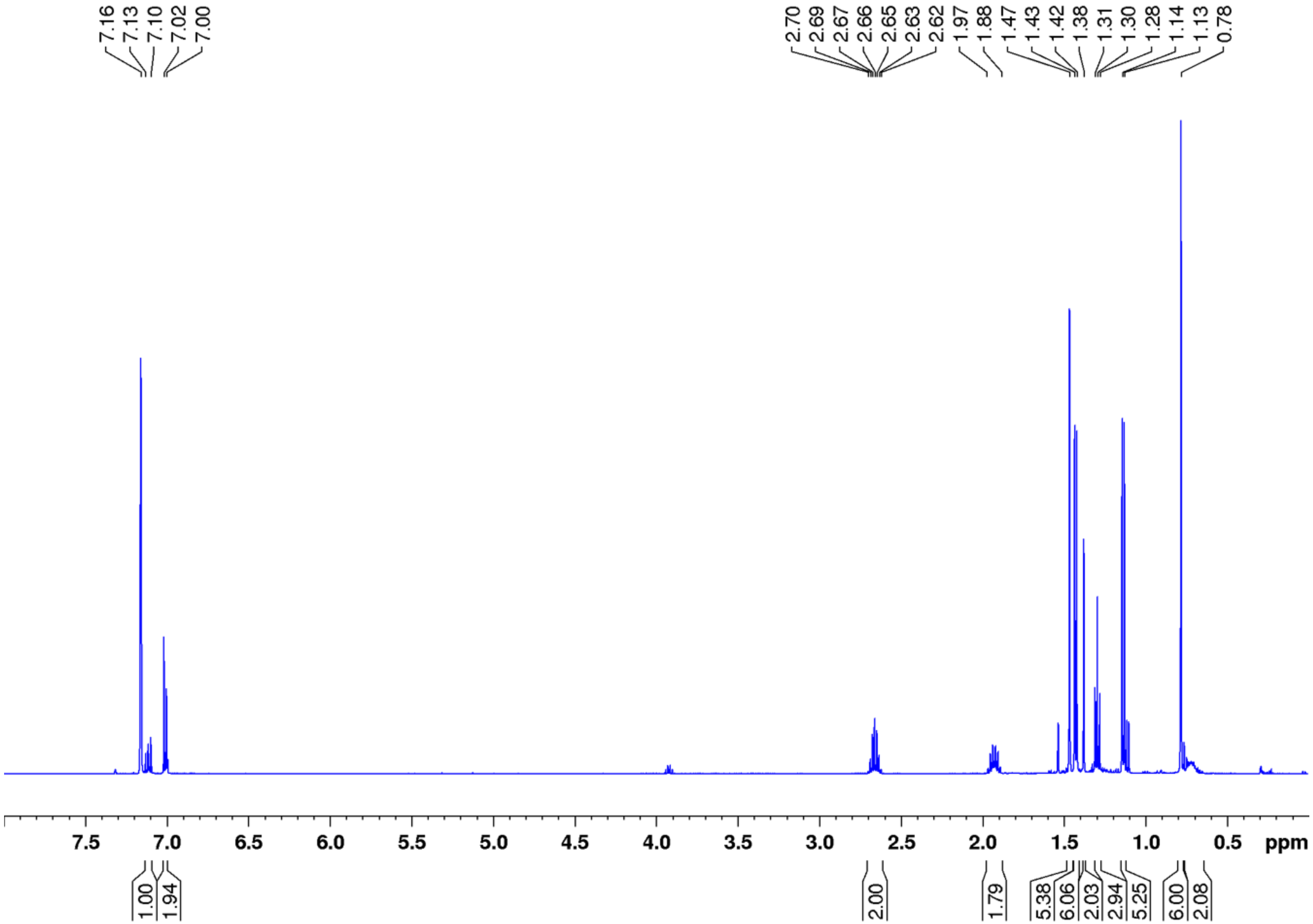

Figure S19. ^{1}H NMR spectrum of $\mathbf{8}^{\boldsymbol{n}\mathbf{Pr}}$ in C_6D_6.

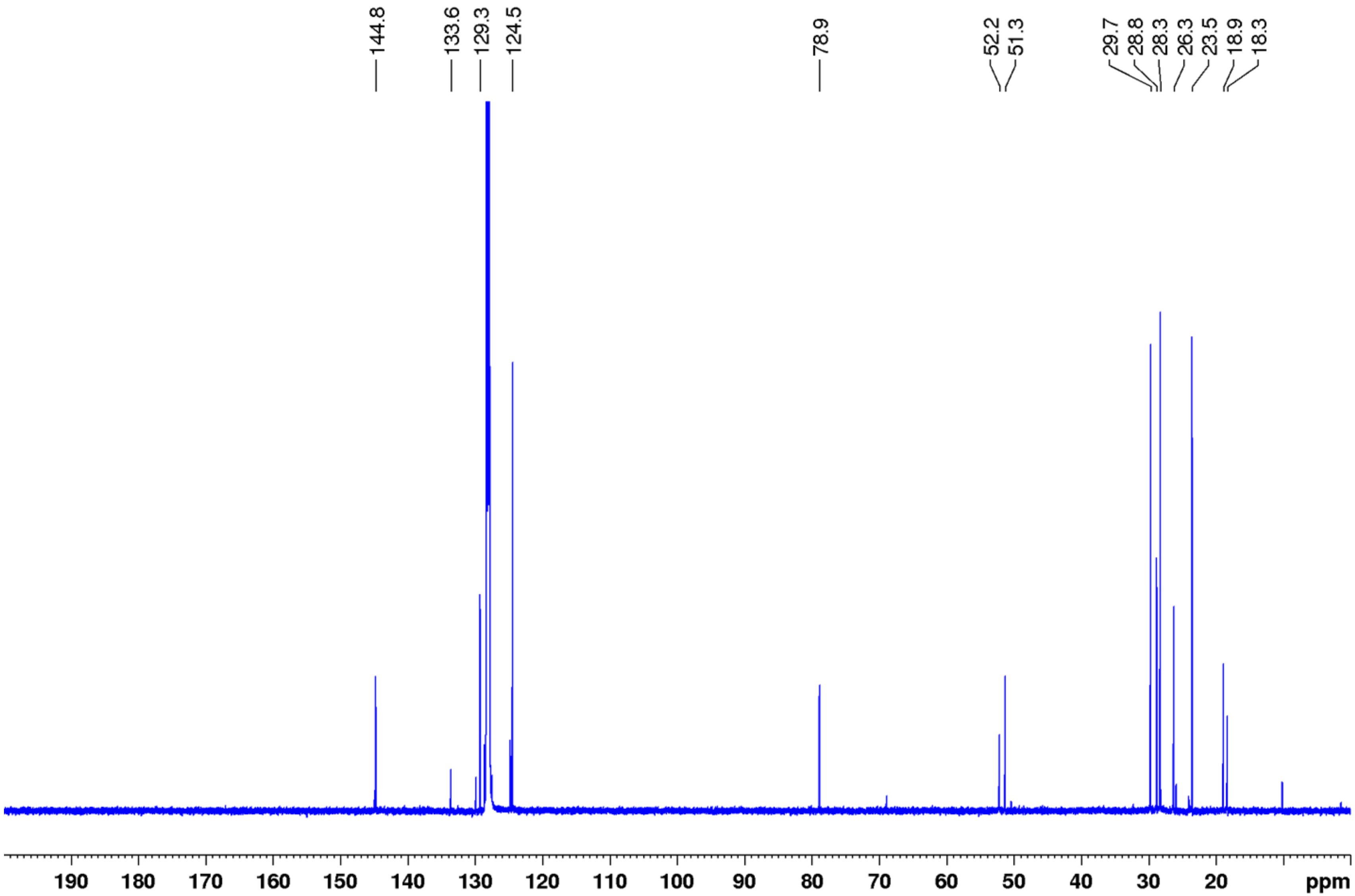

Figure S20. $^{13}C\{^{1}H\}$ NMR spectrum of **8**$^{n\mathbf{Pr}}$ in C_6D_6.

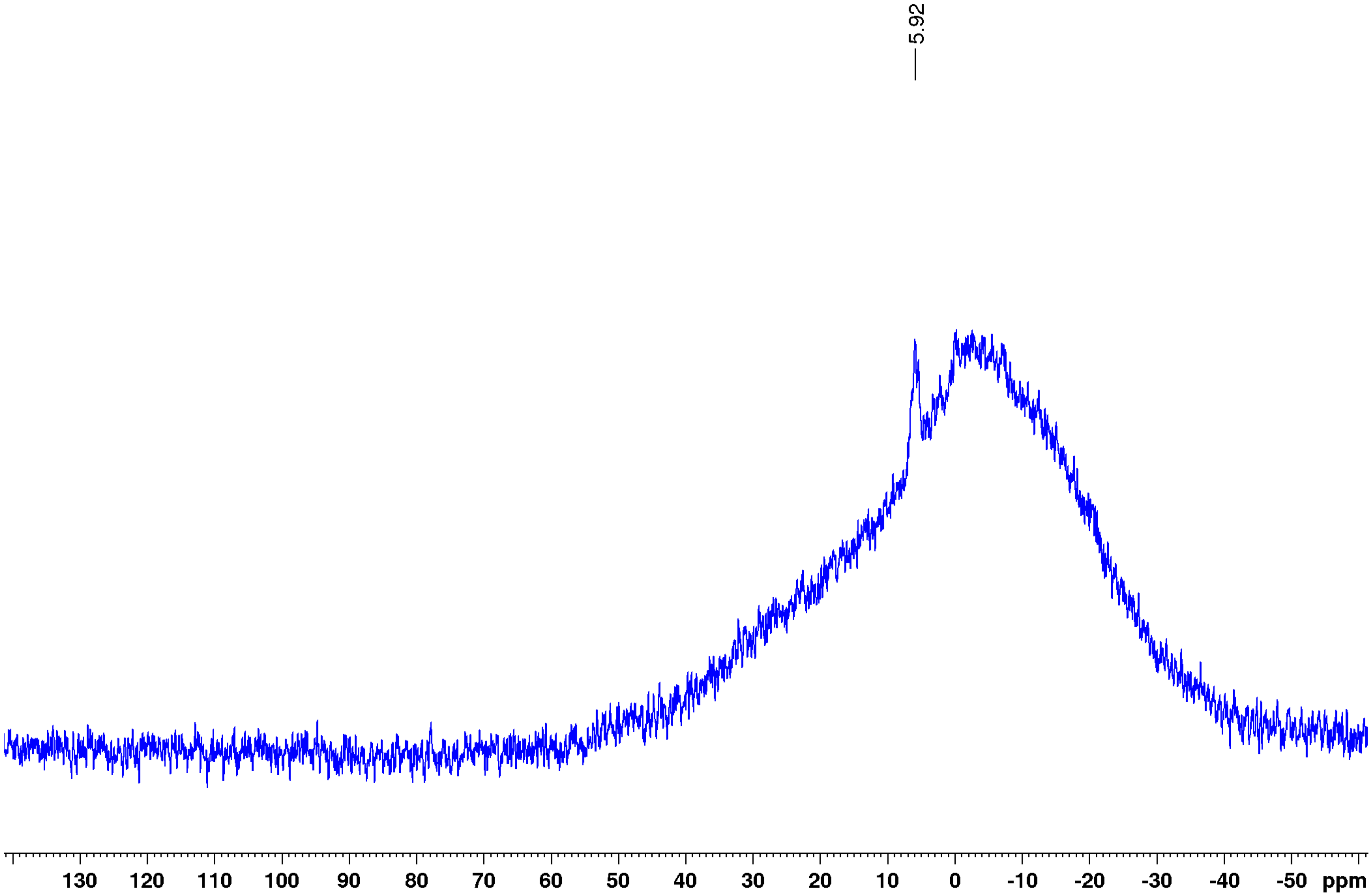

Figure S21. ^{11}B NMR spectrum of $\mathbf{8}^{n\mathbf{Pr}}$ in C_6D_6.

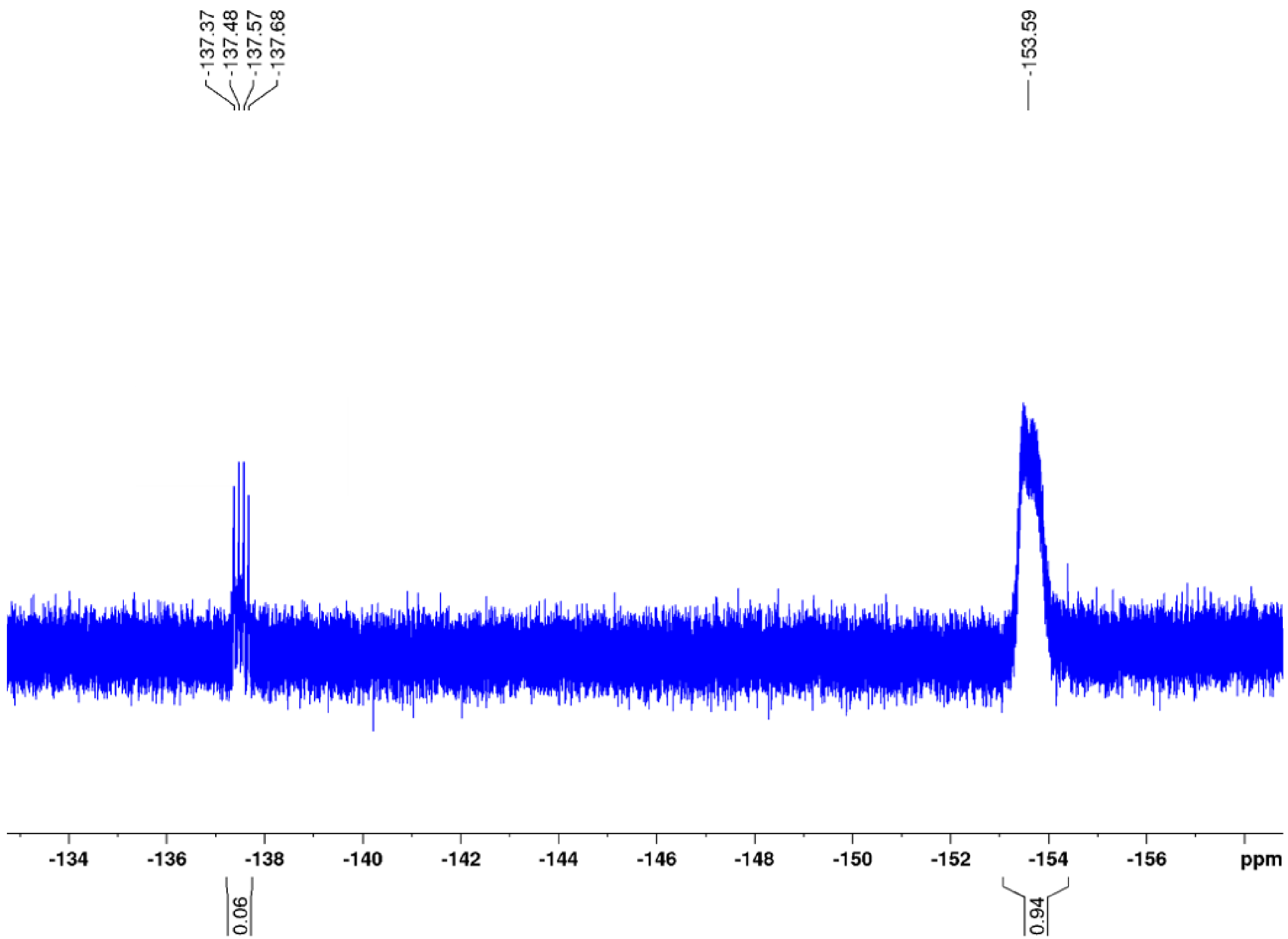

Figure S22. ^{19}F NMR spectrum of $\mathbf{8}^{n\mathbf{Pr}}$ in C_6D_6. The quartet at –137.5 ppm corresponds to residual **1**, which could not be removed by fractional crystallisation (ca. 6%).

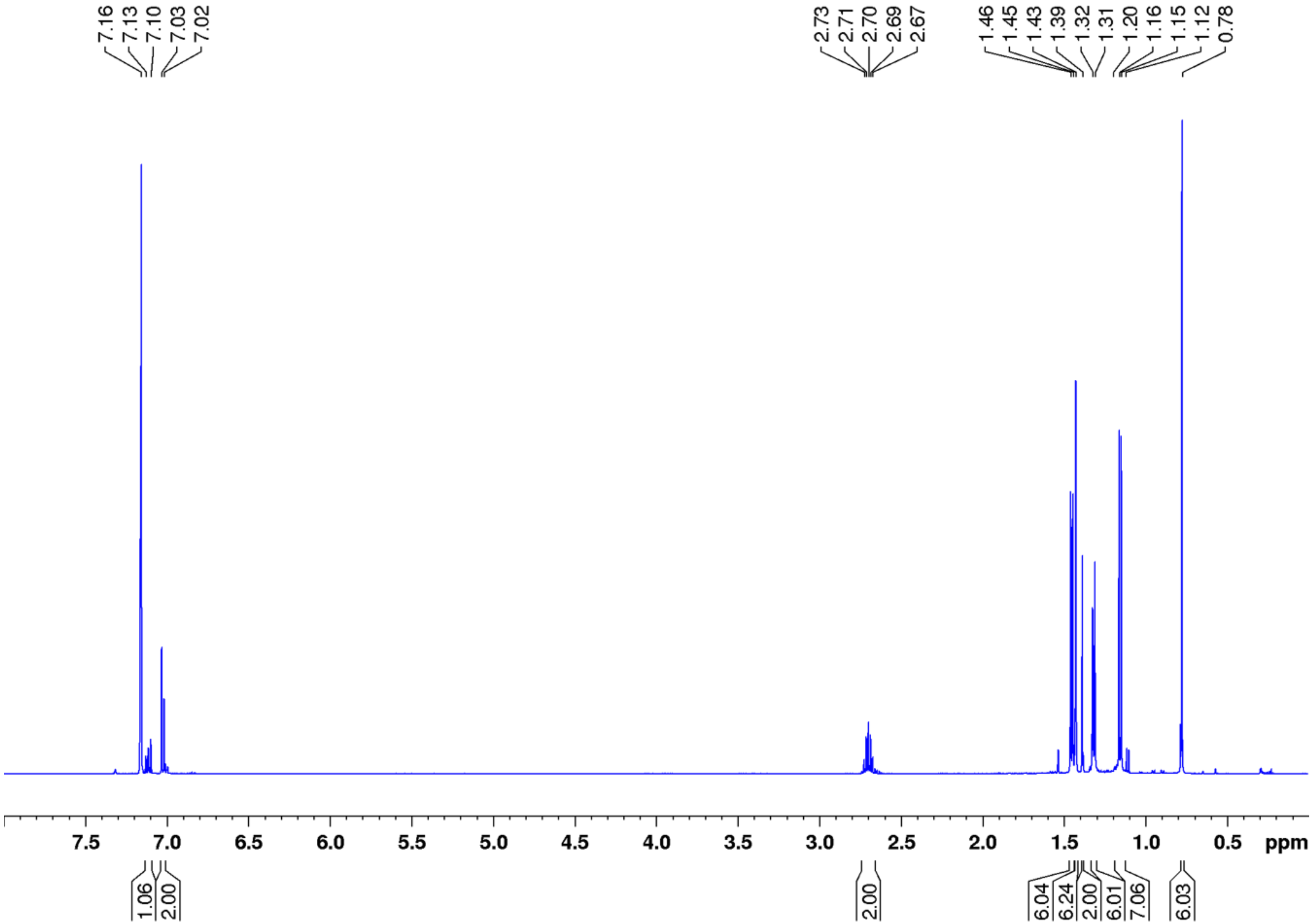

Figure S23. ^{1}H NMR spectrum of $\mathbf{8}^{\boldsymbol{i}\mathbf{Pr}}$ in C_6D_6.

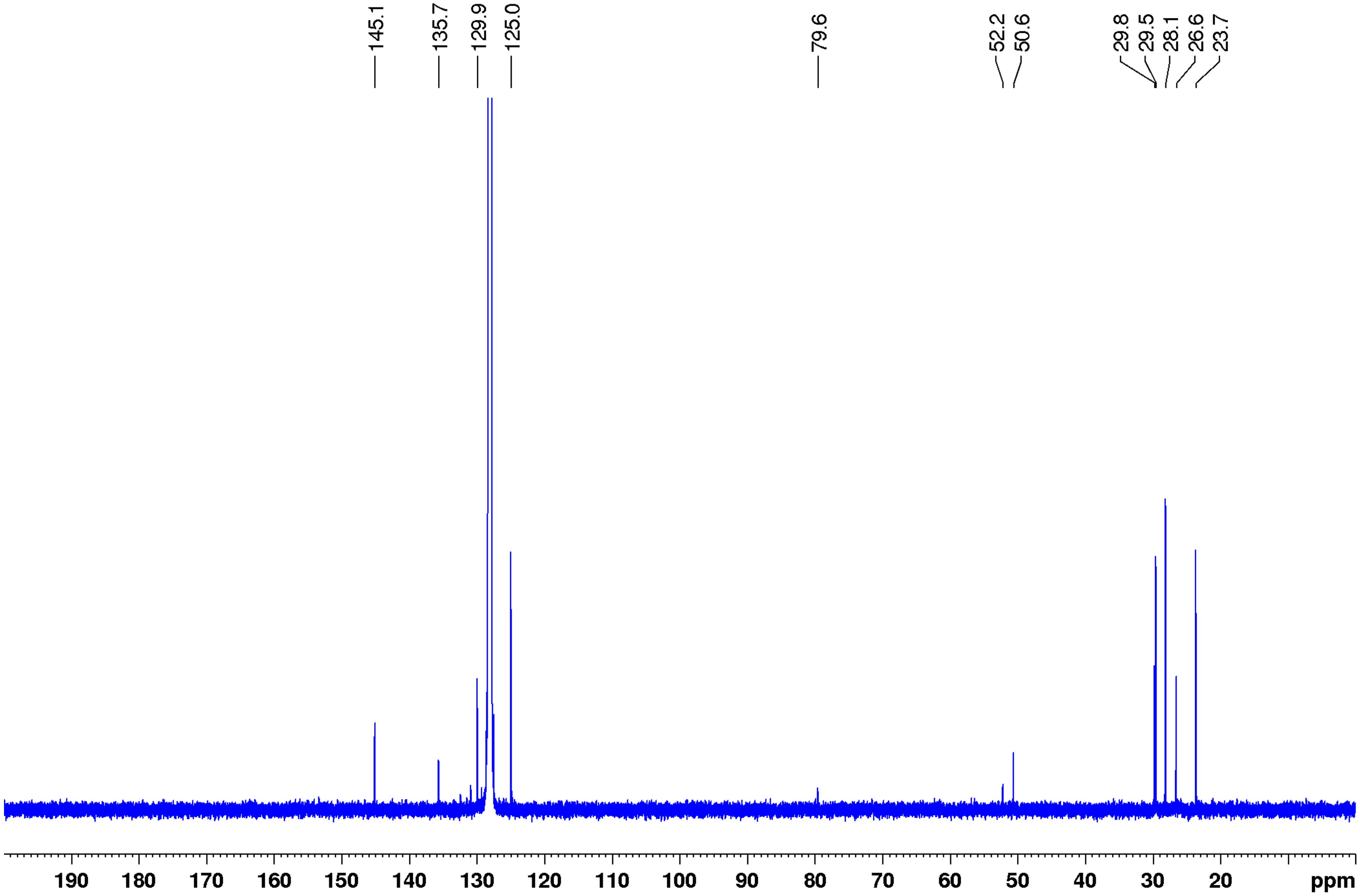

Figure S24. $^{13}C\{^{1}H\}$ NMR spectrum of **8^{iPr}** in C_6D_6.

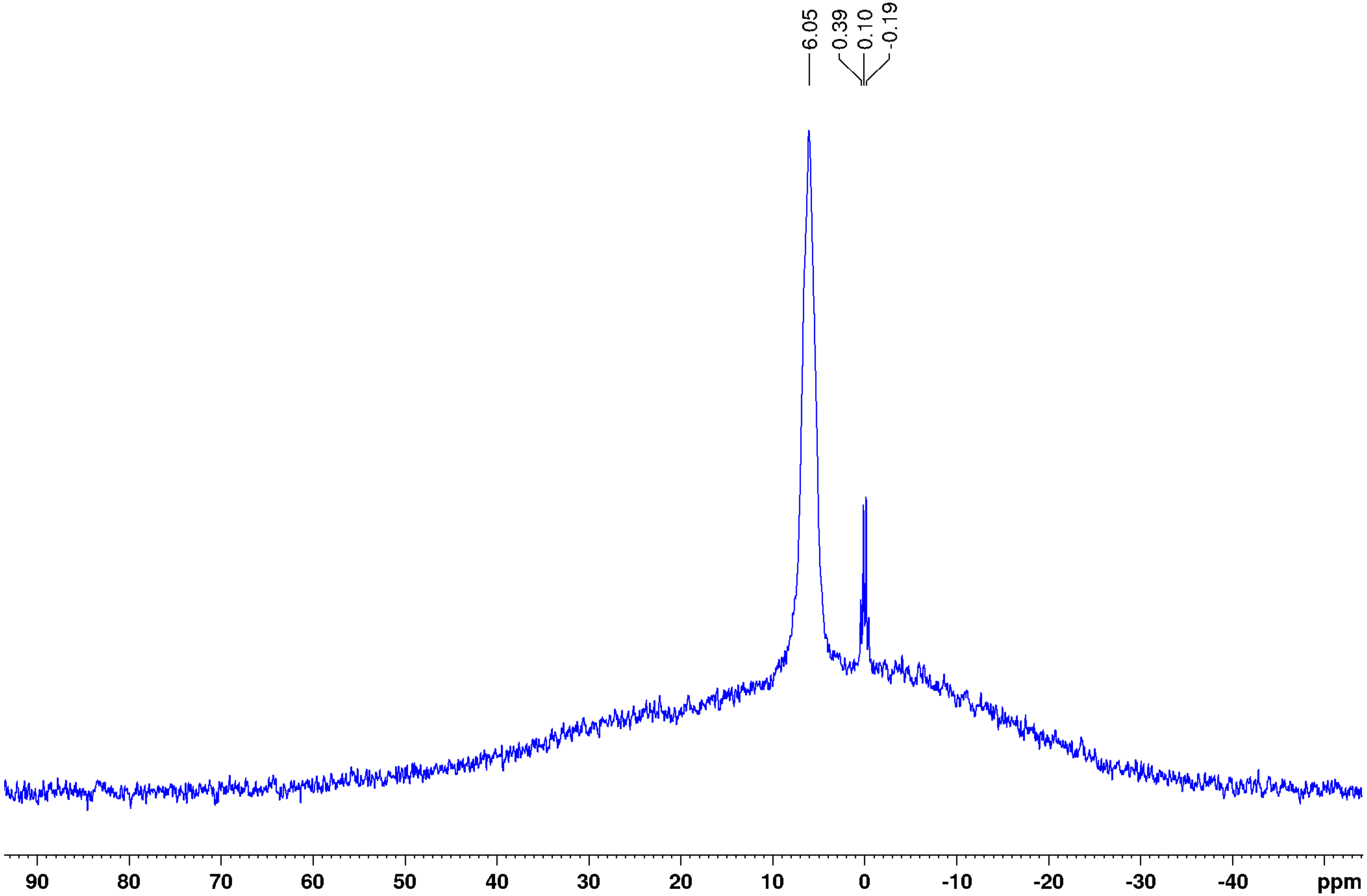

Figure S25. ^{11}B NMR spectrum of $\mathbf{8}^{\boldsymbol{i}\mathbf{Pr}}$ in C_6D_6. The quartet at –2 ppm corresponds to residual **1**, which could not be removed by fractional crystallisation (ca. 6% by ^{19}F NMR).

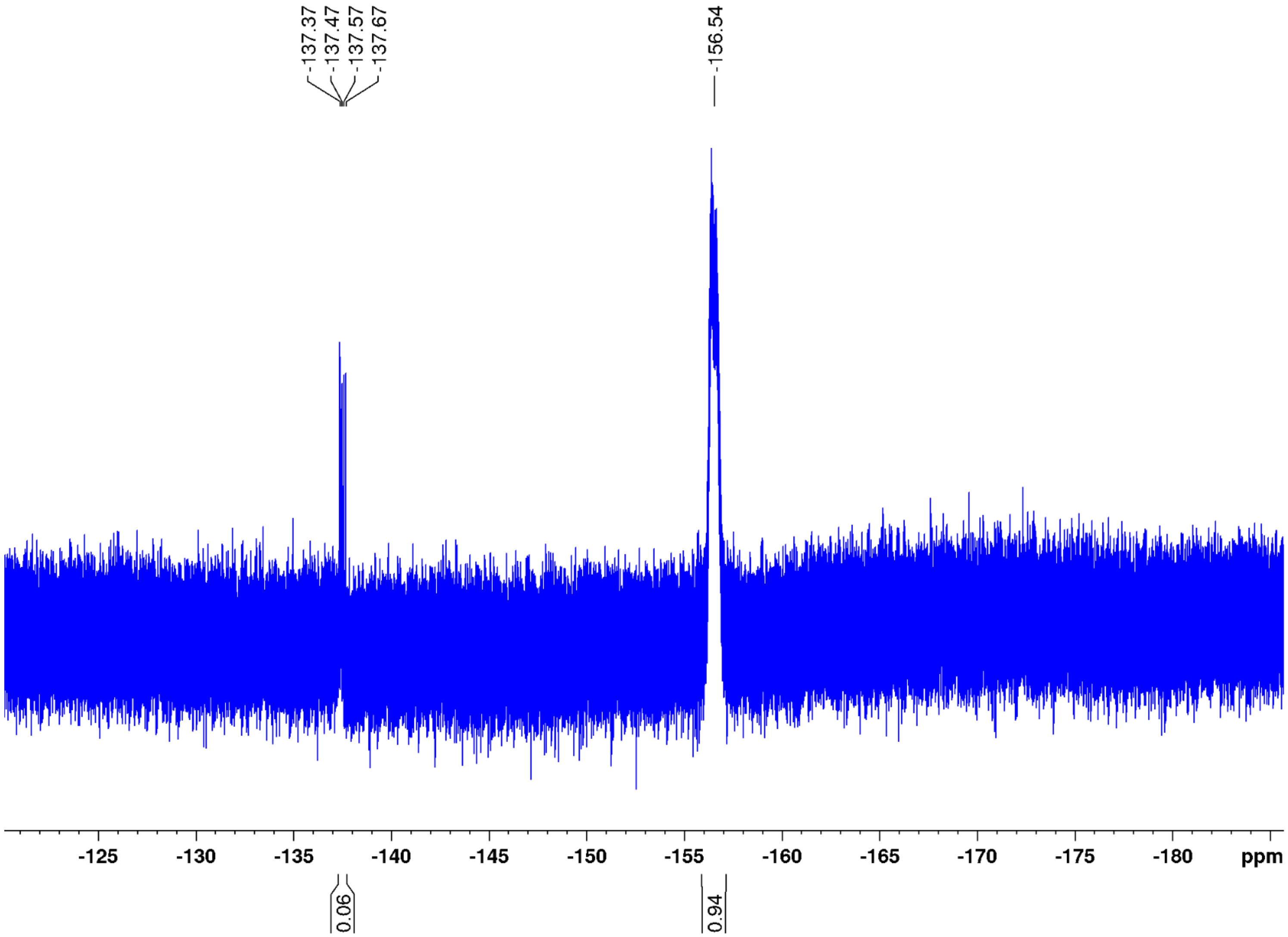

Figure S26. ^{19}F NMR spectrum of $\mathbf{8}^{\boldsymbol{i}\mathbf{Pr}}$ in C_6D_6. The quartet at –137.5 ppm corresponds to residual **1**, which could not be removed by fractional crystallisation (ca. 6%).

***In-situ* generation of 6a/b, 7^{Li} and 7^{K}**

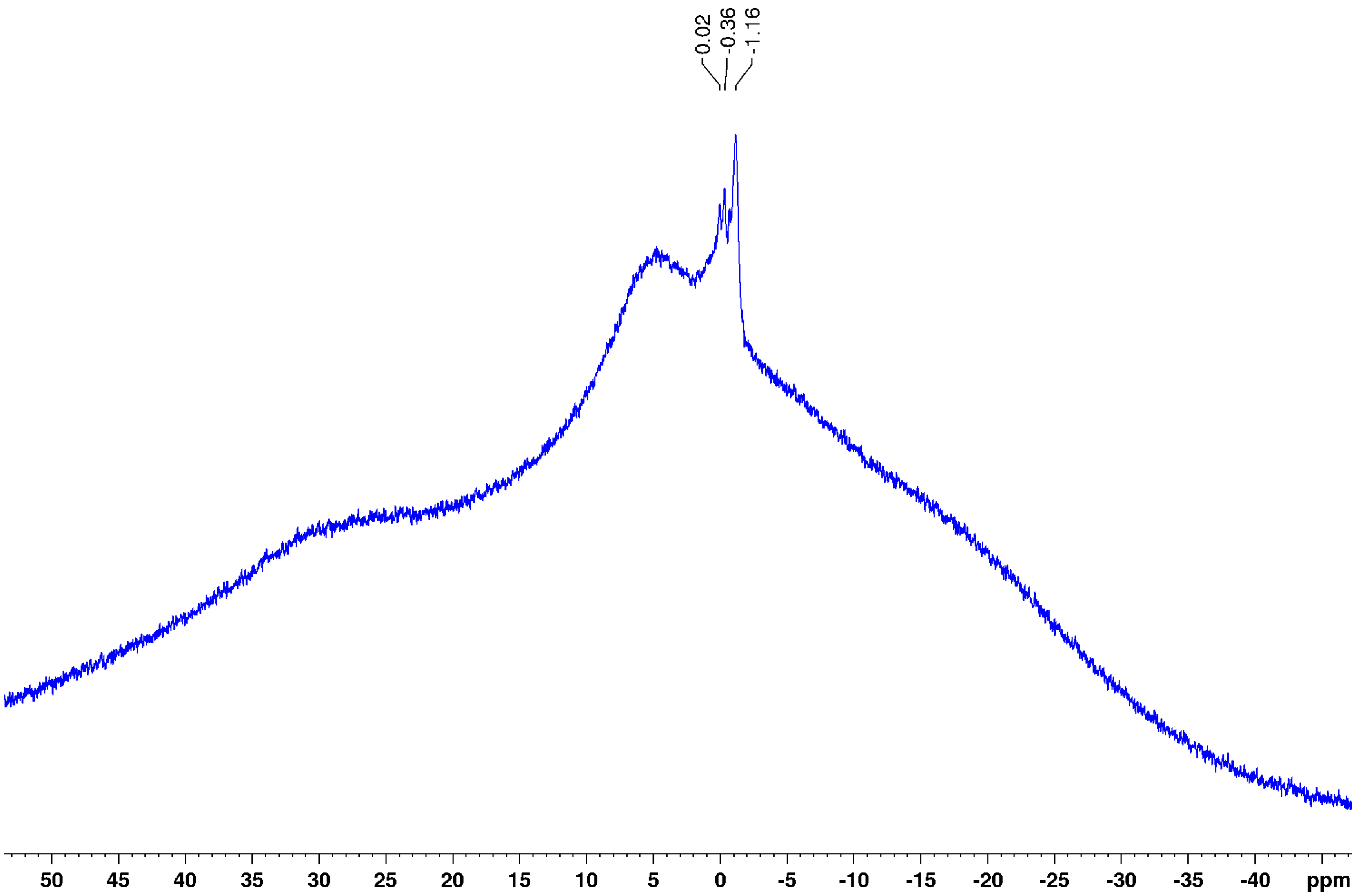

Figure S27. ^{11}B NMR spectrum of **6a/b** in C_6D_6.

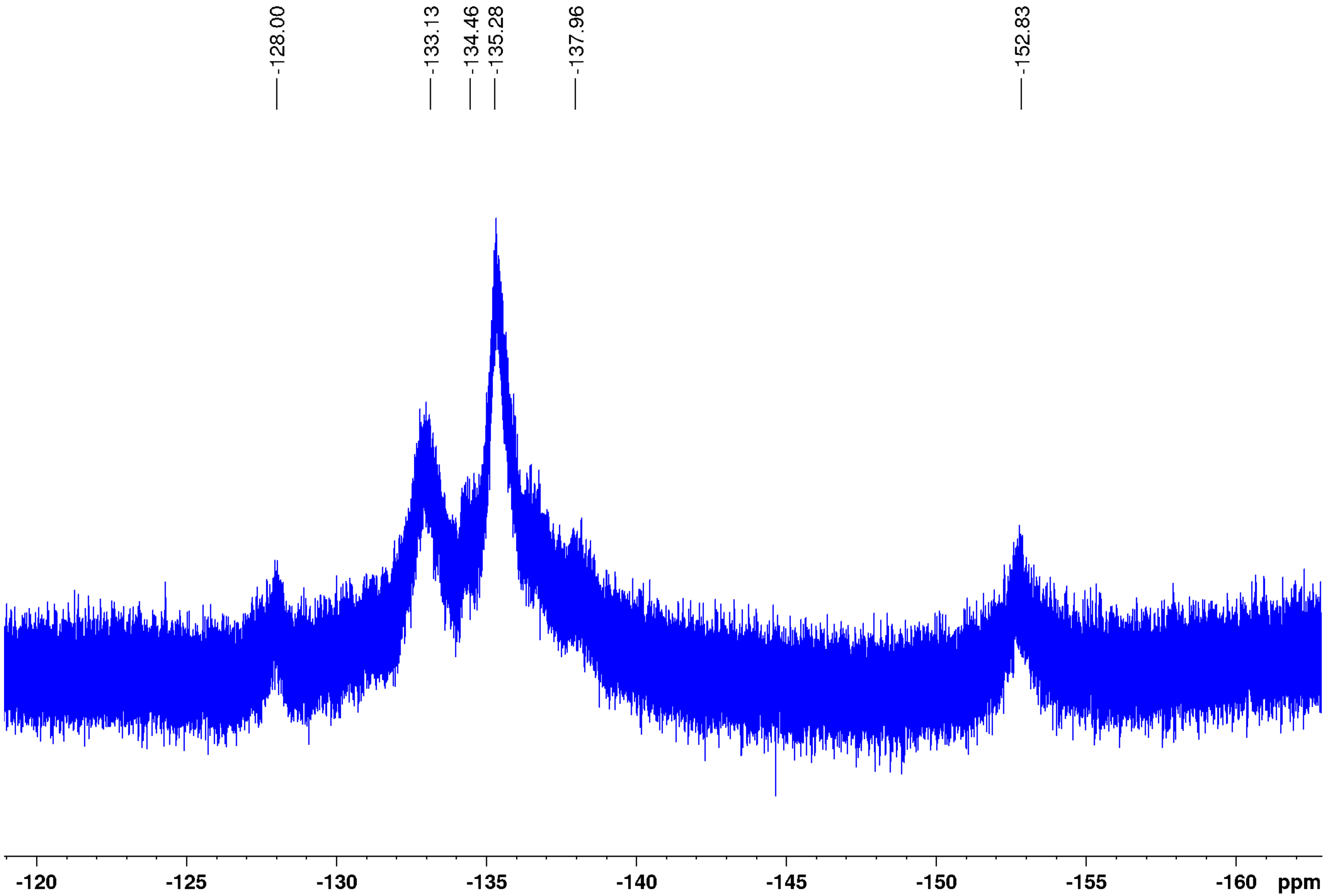

Figure S28. ^{19}F NMR spectrum of **6a/b** in C_6D_6.

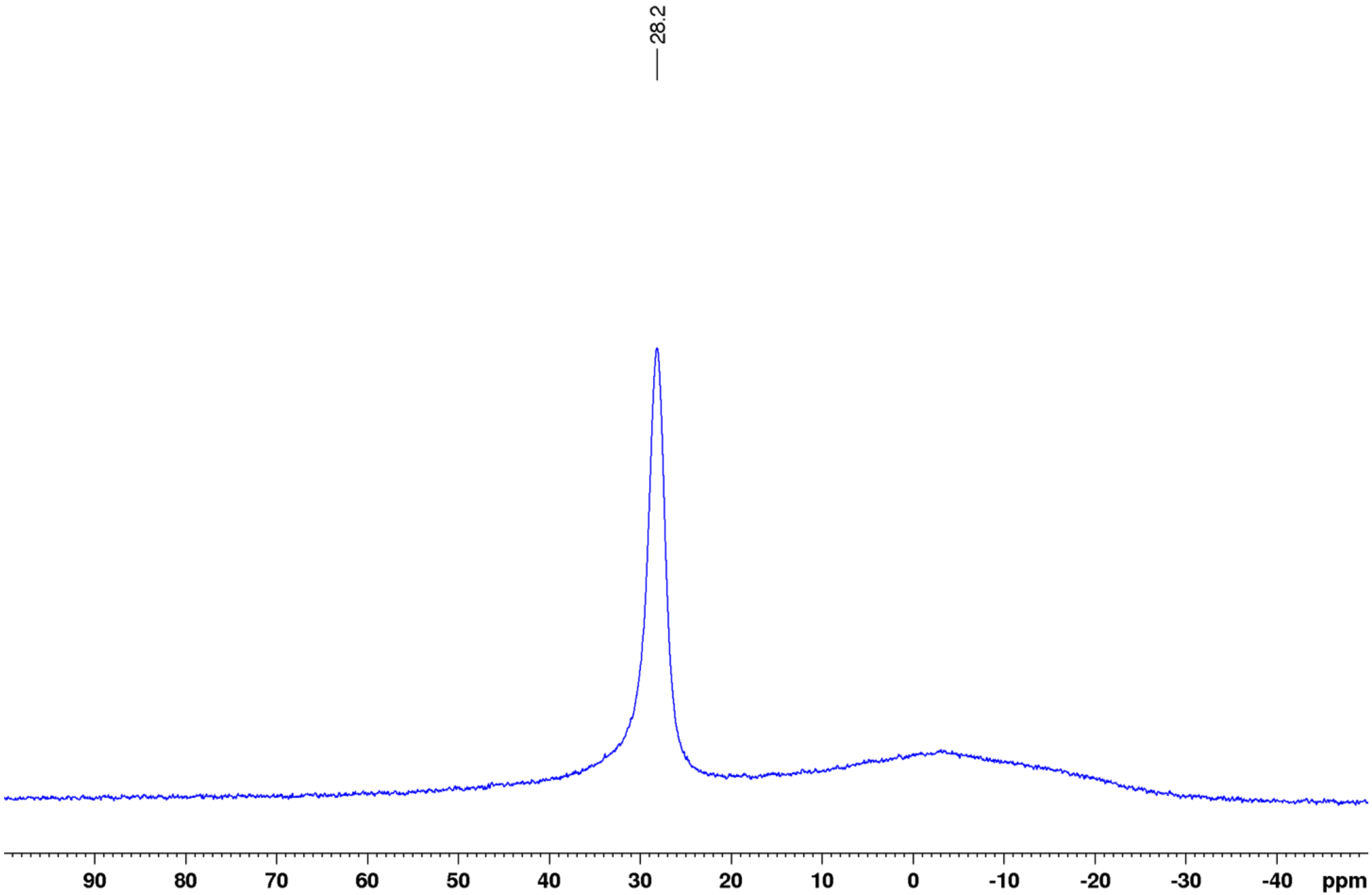

Figure S29. ^{11}B NMR spectrum of $\mathbf{7^{Li}}$ in THF.

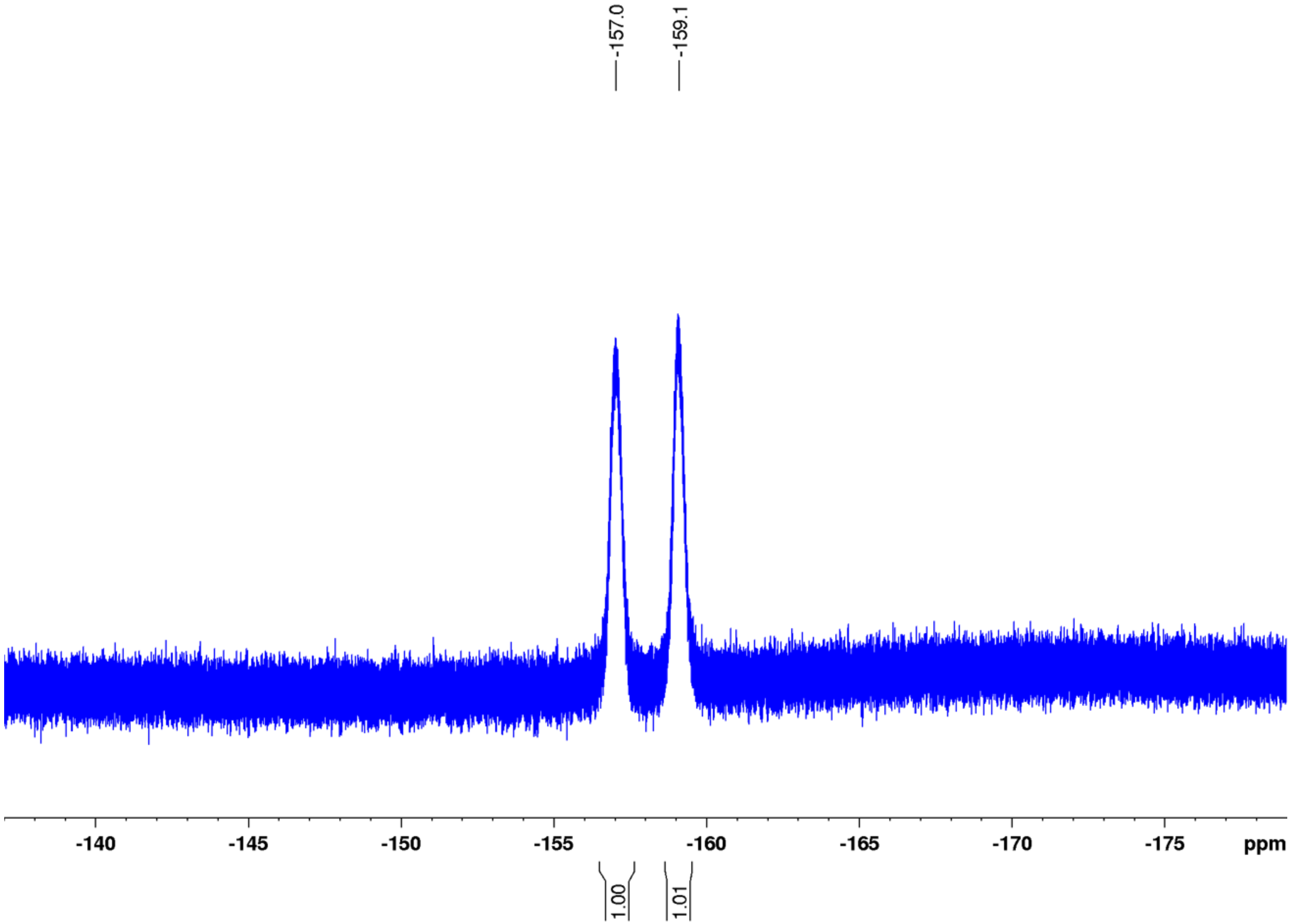

Figure S30. ^{19}F NMR spectrum of $\mathbf{7^{Li}}$ in THF.

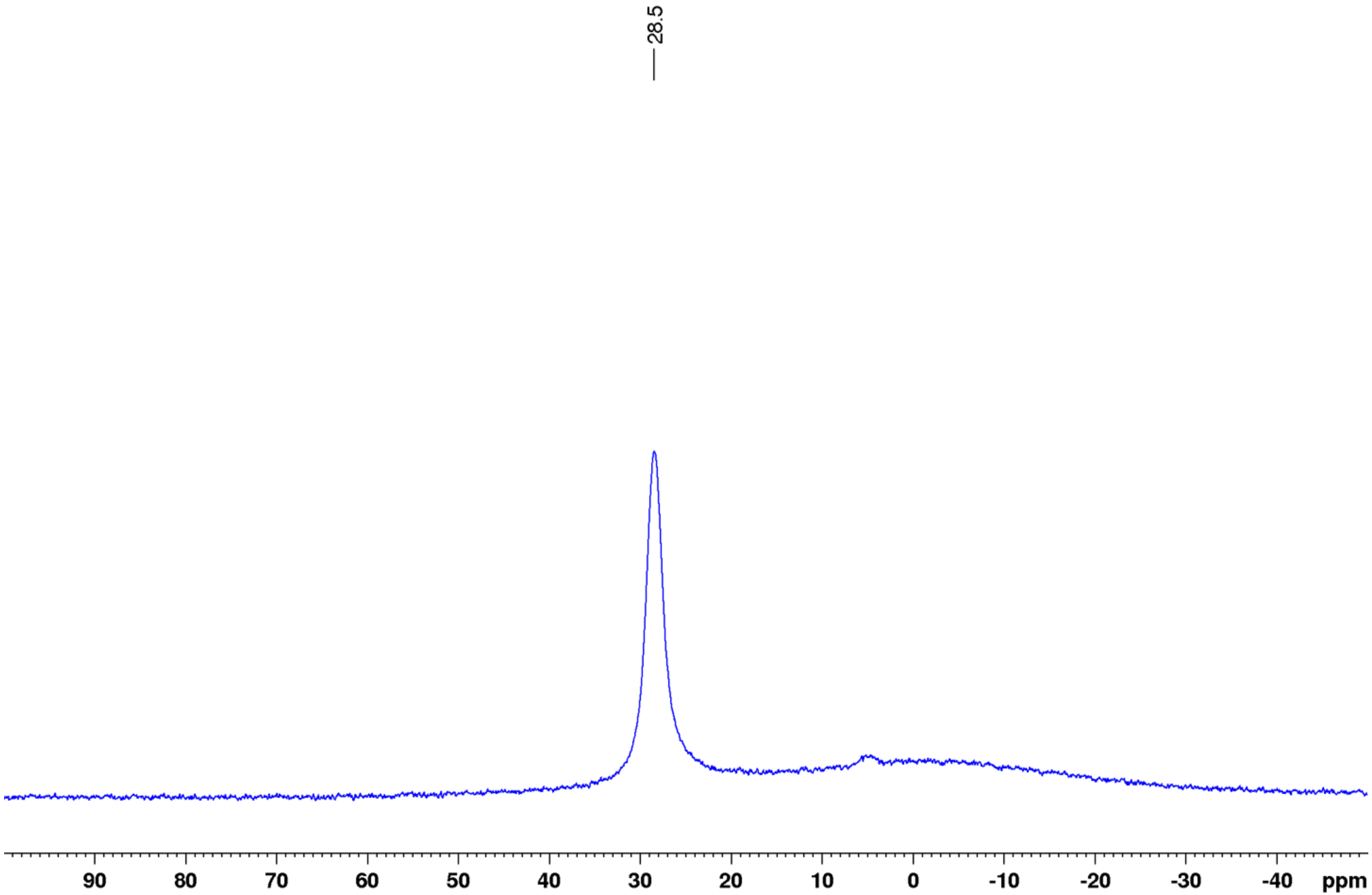

Figure S31. ^{11}B NMR spectrum of $\mathbf{7^K}$ in Et_2O.

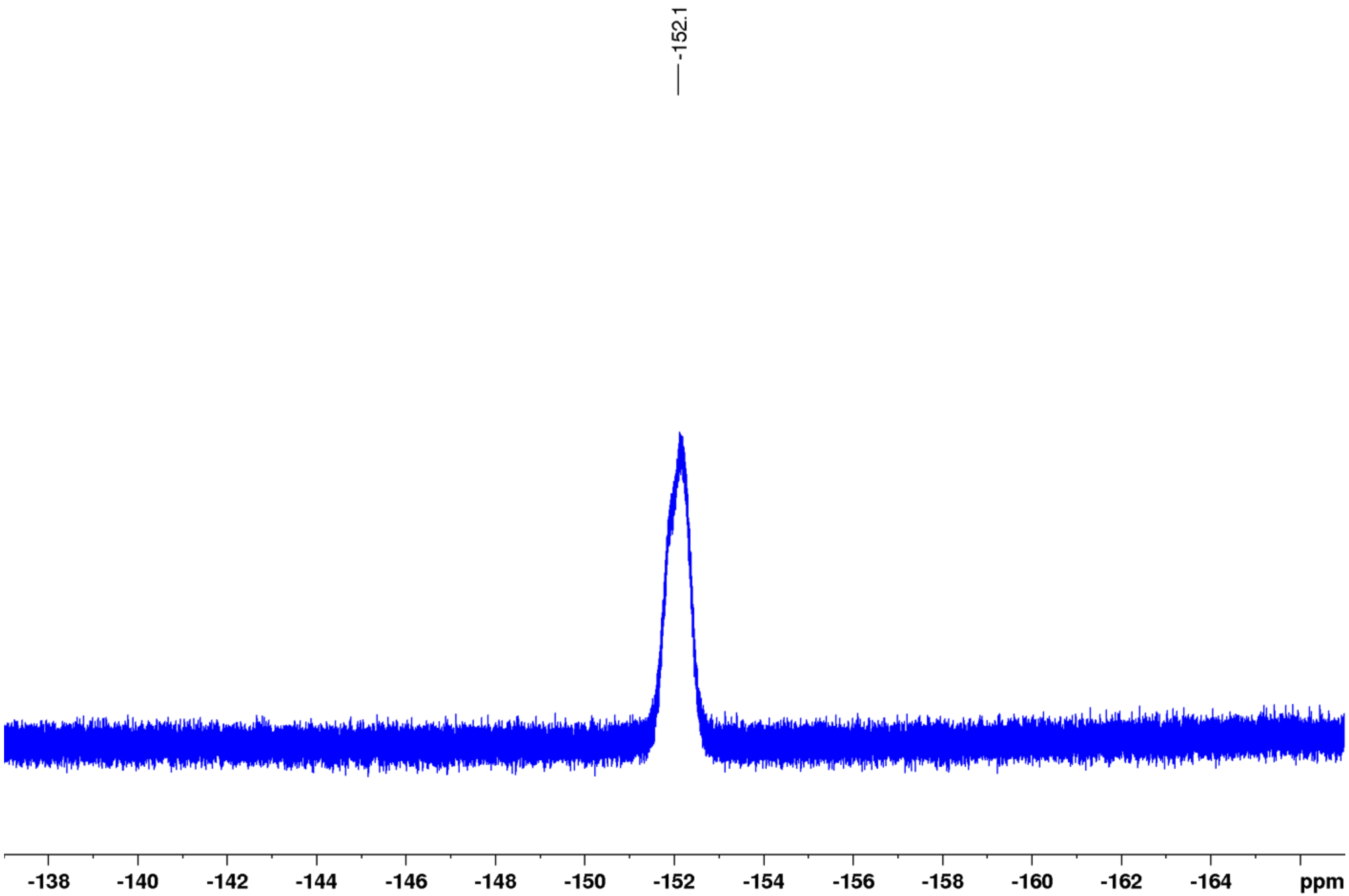

Figure S32. ^{19}F NMR spectrum of $\mathbf{7^K}$ in Et_2O.

Disproportionation of 7[Na] into cumulene V and radical 2

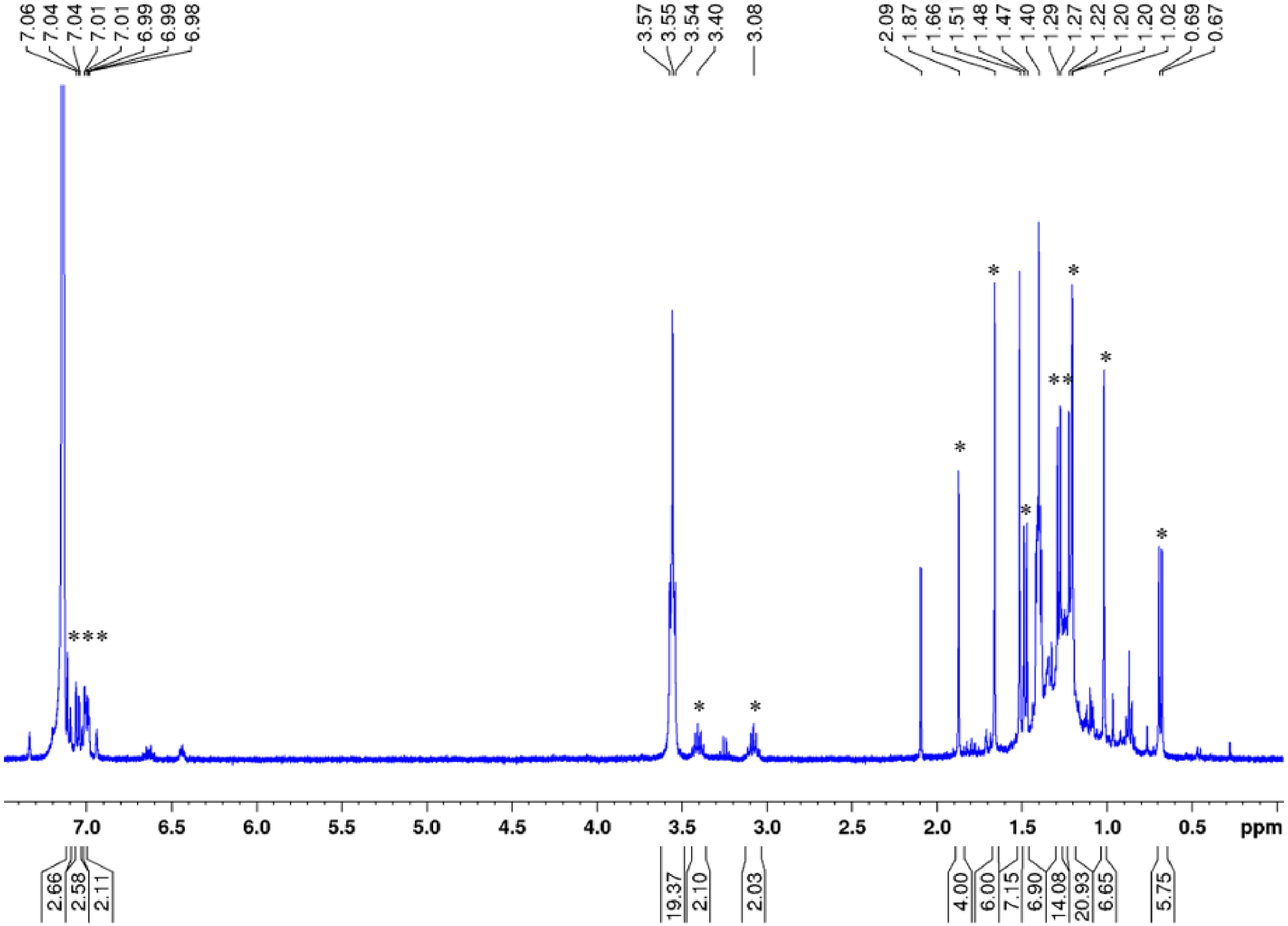

Figure S33. ^{1}H NMR spectrum of a solution of **7[Na]** in C_6D_6 left at rt for 2 weeks. The resonances marked * belong to the diboracumulene **V**.(ref)

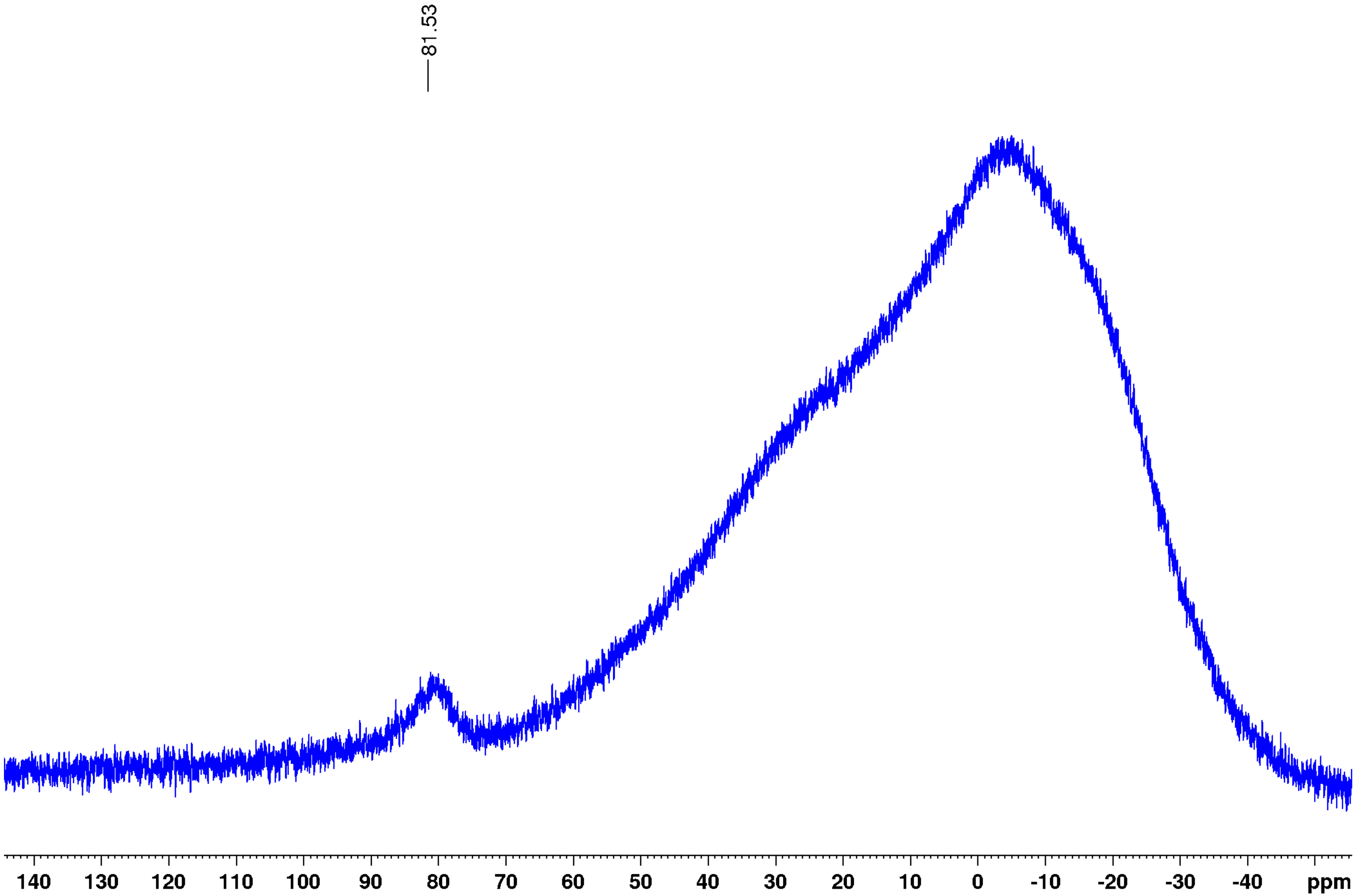

Figure S34. ^{11}B NMR spectrum of a solution of $\mathbf{7^{Na}}$ in C_6D_6 left at rt for 2 weeks, showing only **V**.

EPR spectra

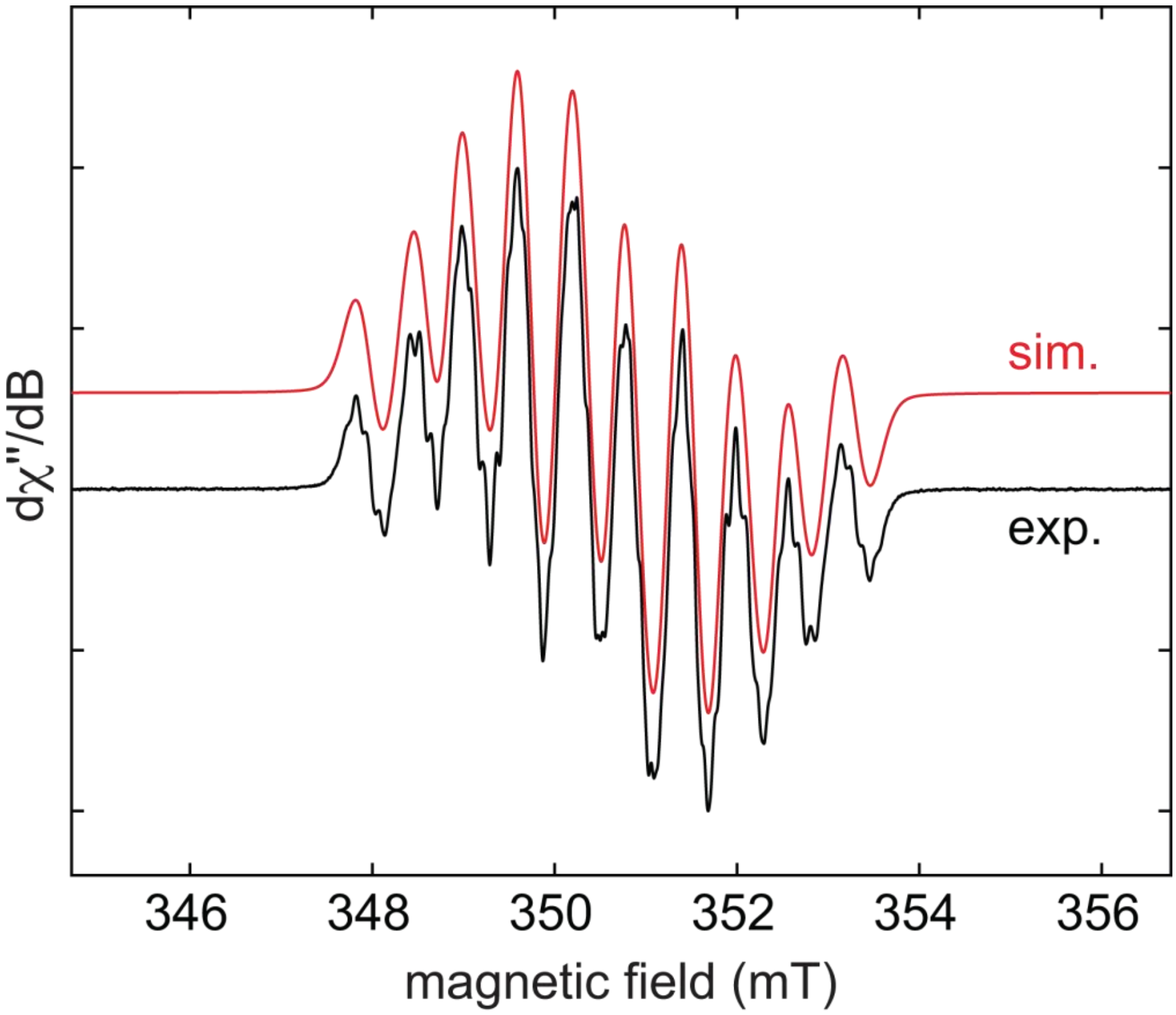

Figure S35. Experimental (black) and simulated (red) continuous-wave (CW) X-band EPR spectra of **2** in benzene at room temperature. The best-fit simulation parameters are g_{iso} = 2.0033; $a(^{10/11}B)$ = 16.4, $a(^{14}N)$ = 19.5, $a(^{19}F, 2F)$ = 30 MHz. Experimental parameters: microwave frequency = 9.83 GHz; microwave power = 0.2 mW; modulation amplitude = 0.2 G; conversion time = 60 ms; modulation frequency = 100 kHz.

SCXRD details

The crystal data of all compounds were collected on a Rigaku XtaLAB Synergy-R diffractometer with a HPA area detector and multi-layer mirror monochromated $Cu_{K\alpha}$ radiation. The structure was solved using intrinsic phasing method,[5] refined with the ShelXL program[6] and expanded using Fourier techniques. All non-hydrogen atoms were refined anisotropically. All hydrogen atoms were assigned to idealised positions unless specified otherwise.

Crystallographic data have been deposited with the Cambridge Crystallographic Data Center as supplementary publication no. CCDC 2564348 (**4**), 2564349 (**6**), 2564350 (**5**), 2564351 (**3**), 2564352 (**1**), and 2564353 (**7**$^{\mathbf{Na}}$), 2588263 (**8**$^{\boldsymbol{i}\mathbf{Pr}}$), and 2588264 (**8**$^{\boldsymbol{n}\mathbf{Pr}}$). These data can be obtained free of charge from The Cambridge Crystallographic Data Centre *via* www.ccdc.cam.ac.uk/data_request/cif.

Compound 1

Crystal data for 1: $C_{26}H_{37}BF_3N$, $M_r = 431.38$, colourless block, 0.350×0.160×0.080 mm^3, monoclinic space group $P2_1/n$, $a = 7.86000(10)$ Å, $b = 14.4395(3)$ Å, $c = 21.9488(4)$ Å, $\beta = 93.691(2)°$, $V = 2485.90(8)$ Å^3, $Z = 4$, $\rho_{calcd} = 1.153$ g·cm^{-3}, $\mu = 0.655$ mm^{-1}, $F(000) = 928$, $T = 100(2)$ K, $R_1 = 0.0527$, $wR_2 = 0.1178$, 5047 independent reflections [$2\theta \leq 148.982°$] and 288 parameters.

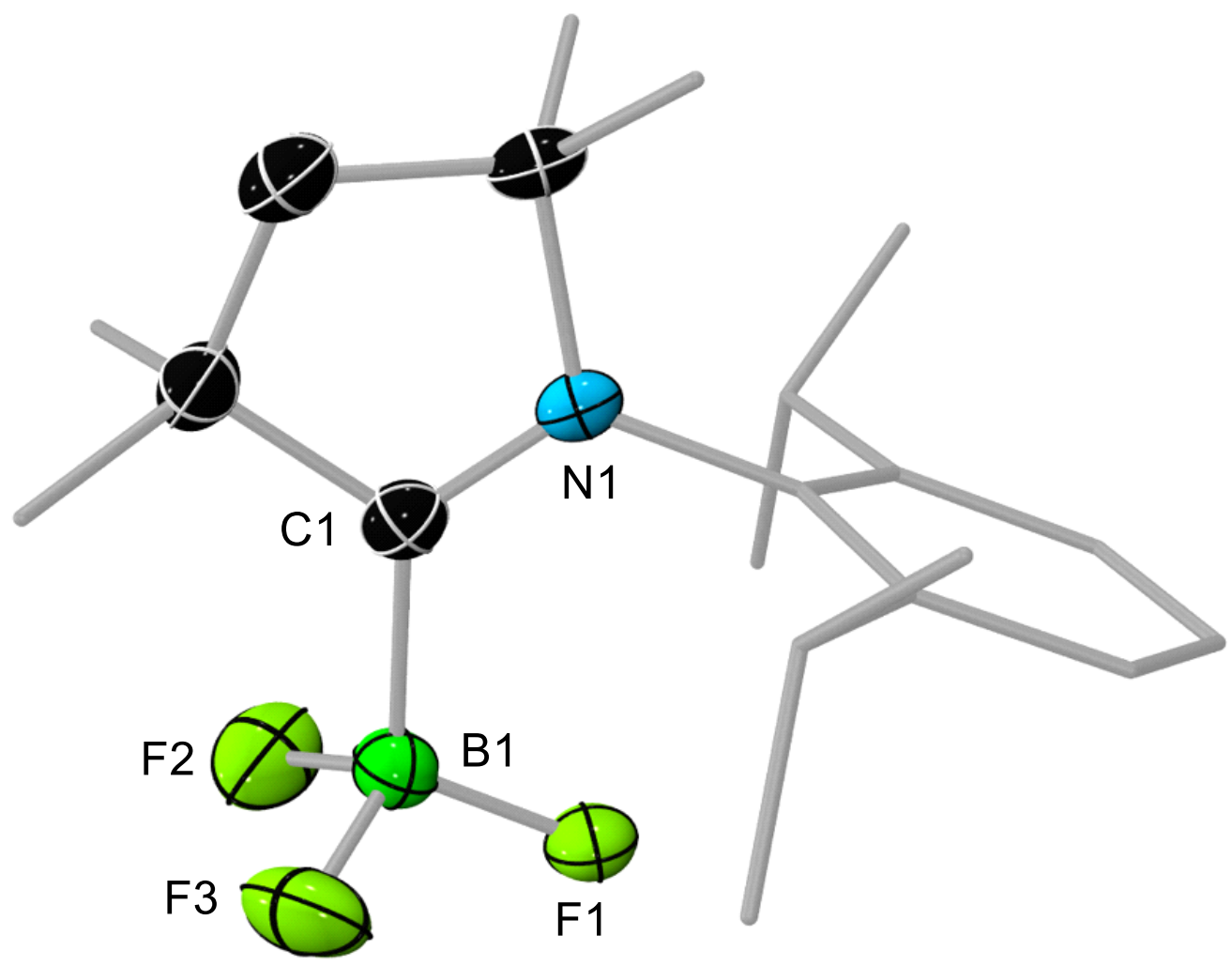

Figure S36. SCXRD-derived solid-state structure of **1**. Atomic displacement ellipsoids set at 50% probability. Ellipsoids of ligand periphery and hydrogen atoms omitted for clarity.

Compound 3

Crystal data for 3: $C_{26}H_{36}BF_2NS$, $M_r = 443.43$, colourless block, 0.330×0.200×0.120 mm^3, monoclinic space group $P2_1/n$, $a = 11.91520(10)$ Å, $b = 16.36970(10)$ Å, $c = 12.98810(10)$ Å, $\beta = 103.2870(10)°$, $V = 2465.49(3)$ Å^3, $Z = 4$, $\rho_{calcd} = 1.195$ g·cm^{-3}, $\mu = 1.386$ mm^{-1}, $F(000) = 952$, $T = 100(2)$ K, $R_1 = 0.0401$, $wR_2 = 0.0981$, 4821 independent reflections [$2\theta \leq 142.242°$] and 288 parameters.

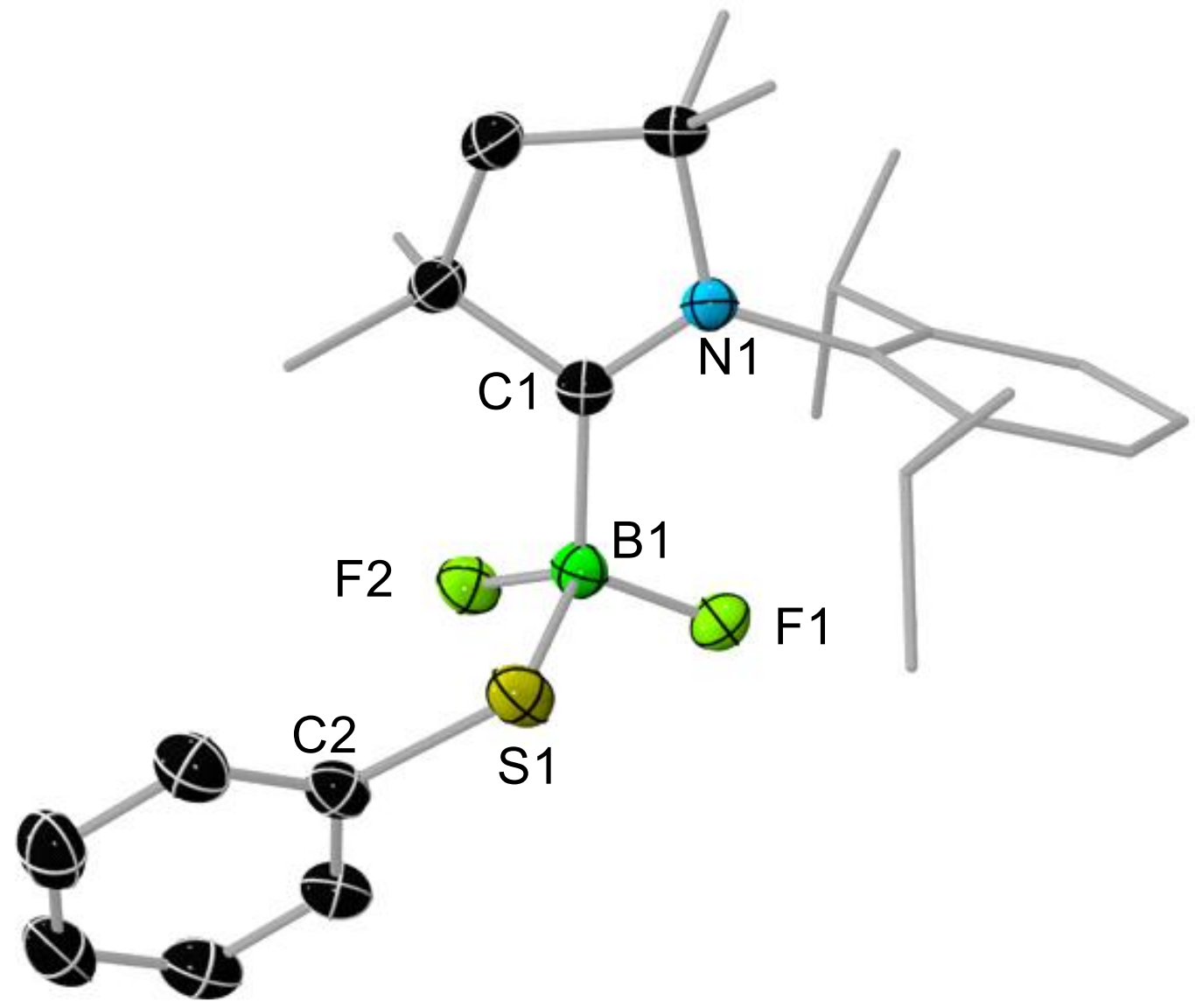

Figure S37. SCXRD-derived solid-state structure of **3**. Atomic displacement ellipsoids set at 50% probability. Ellipsoids of ligand periphery and hydrogen atoms omitted for clarity.

Compound 4

Special refinement details for 4: Refined as a two-component twin. Component 2 rotated by 5.8955° around [0.52 –0.09 0.85] (reciprocal) or [0.91 –0.11 0.39] (direct) The BASF parameter was refined to 19.7%.

Crystal data for 4: $C_{29}H_{49}B_2F_2N_2O$, M_r = 490.51, colourless block, 0.229×0.139×0.103 mm^3, monoclinic space group $P2_1/n$, a = 10.8763(2) Å, b = 12.0696(2) Å, c = 21.8234(3) Å, β = 100.219(2)°, V = 2819.37(8) $Å^3$, Z = 4, ρ_{calcd} = 1.156 g·cm^{-3}, μ = 0.614 mm^{-1}, $F(000)$ = 1072, T = 100(2) K, R_1 = 0.0723, wR_2 = 0.1527, 10877 independent reflections [$2\theta \leq 140.136°$] and 329 parameters.

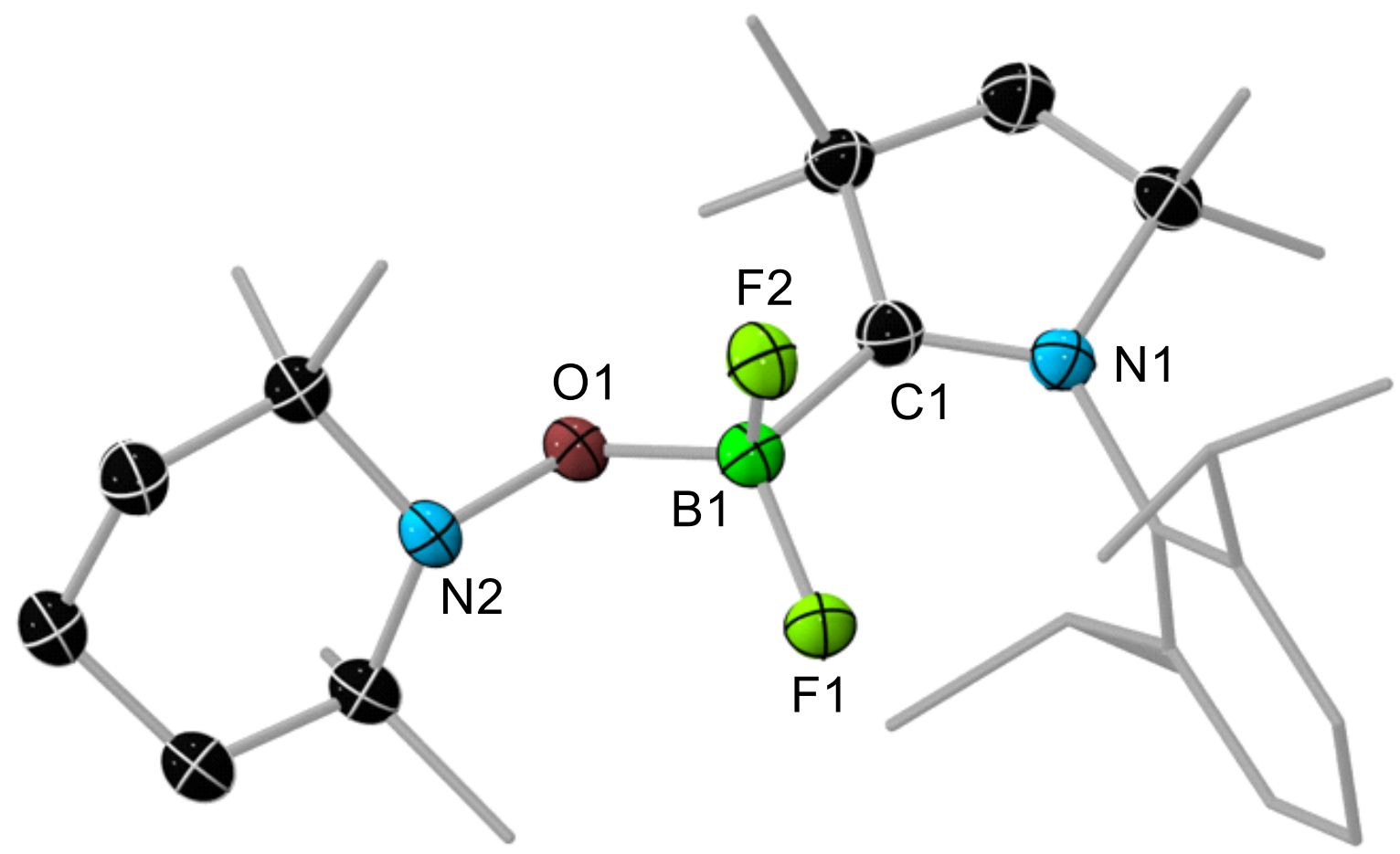

Figure S38. SCXRD-derived solid-state structure of **4**. Atomic displacement ellipsoids set at 50% probability. Ellipsoids of ligand periphery and hydrogen atoms omitted for clarity.

Compound 5

Crystal data for 5: $C_{46}H_{66}B_2F_4N_2O_2$, M_r = 776.62, colourless plate, 0.110×0.060×0.050 mm^3, monoclinic space group $P2_1/c$, a = 8.171(4) Å, b = 13.683(2) Å, c = 19.341(4) Å, β = 92.66(3)°, V = 2160.2(11) Å^3, Z = 2, ρ_{calcd} = 1.194 g·cm^{-3}, μ = 0.667 mm^{-1}, F(000) = 836, T = 100(2) K, R_1 = 0.0380, wR_2 = 0.0836, 4226 independent reflections [$2\theta \leq 144.206°$] and 261 parameters.

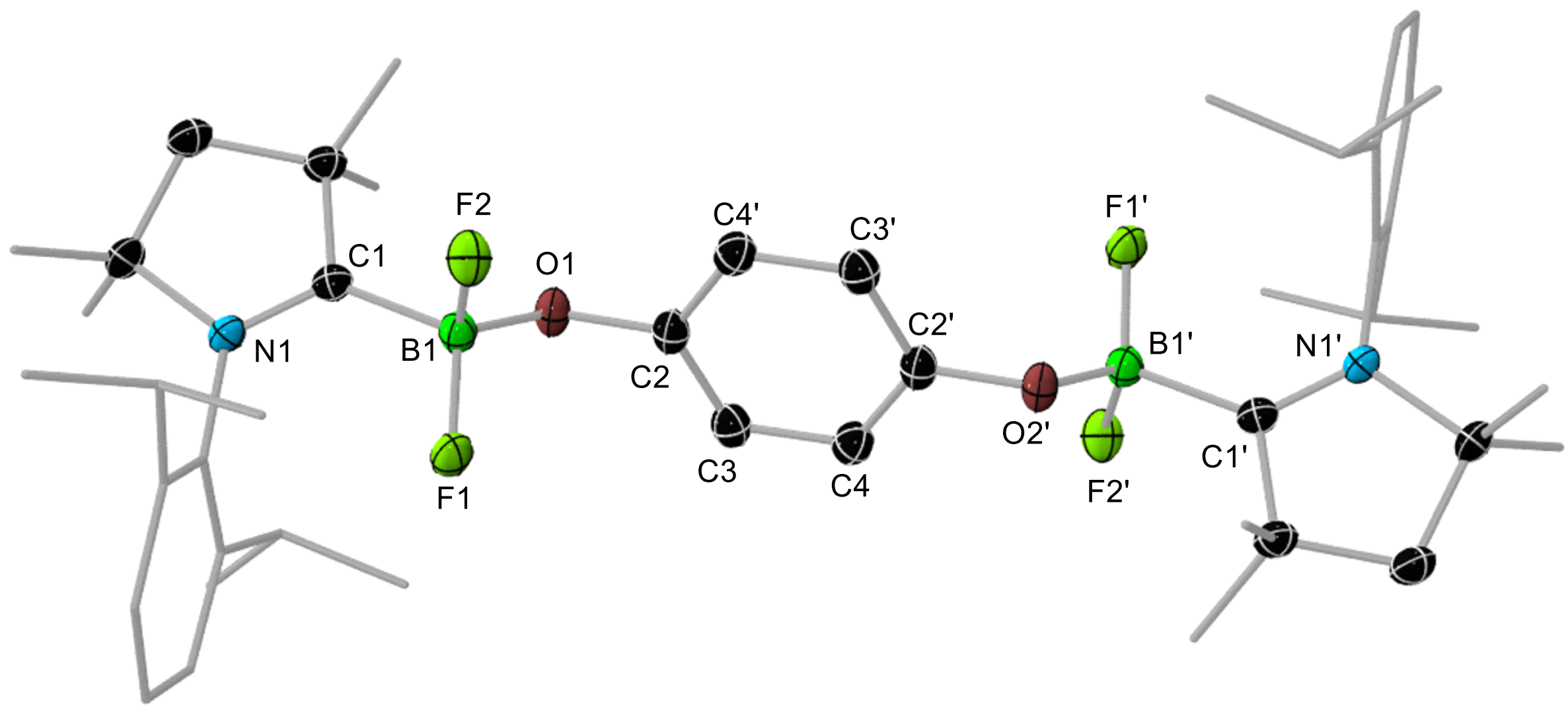

Figure S39. SCXRD-derived centrosymmetric solid-state structure of **5**. Atomic displacement ellipsoids set at 50% probability. Ellipsoids of ligand periphery and hydrogen atoms omitted for clarity.

Compound 6a/6b

Special refinement details for 6a/6b: The asymmetric unit contains a highly disordered pentane molecule, which could not be adequately modelled and was therefore treated as diffuse contribution to the overall electron density by the Platon program Squeeze.[7] 166 electrons were thus removed from the unit cell, corresponding to four molecules of pentane. The asymmetric unit also contains a tetrameric assembly of various (CAAC)BF_3-derived monomers, connected by intermolecular three-center-two-electron B−F−B and electrostatic F···K···F bridges. The main tetrameric unit, **6a** (80%), may be viewed as an assembly of two neutral [(CAACH)BF_2] borane (RESI 11 (80%) and 411/412 (60%) or its enantiomer 421/422 (20%)), a [(CAACH)BF_3]K borate (RESI 311/312), and a [(CAACH)BF]K boryl anion unit (RESI 21), or as two [(CAACH)BF_3]K borate (RESI 411/412-421/422 and 311/312) and two [(CAACH)BF]$^{\bullet}$ boryl radical units (RESI 11 and 21). The second tetrameric unit, **6b** (20%), which overlaps with the first, may be viewed as an assembly of a [(CAAC)BF_3] borane (RESI 121/122), 2 [(CAAC)BF_2]K boryl anion (RESI 22 and 431/432), and a [(CAACH)BF_2] borane unit (RESI 321/322). The 1,2- and 1,3-distances in the residues labelled RBF (= (CAACH)BF), BF_3, L (= CAAC) and LH (= CAACH) were restrained to similarity with SAME 0.01, and all ADPs with SIMU 0.005. As a result, the data may only be used for proof of connectivity and not discussed in detail.

Crystal data for 6a/6b: $[C_{80}H_{128}B_4F_8K_2N_4]_{0.8}\cdot[C_{80}H_{125}B_4F_9K_2N_4]_{0.2}\cdot[C_5H_{10}]_{squeezed}$, M_r = 1494.48, orange block, 0.170×0.150×0.100 mm^3, monoclinic space group $P2_1/c$, a = 15.73800(10) Å, b = 16.82410(10) Å, c = 32.9129(2) Å, β = 99.0400(10)°, V = 8606.35(9) Å^3, Z = 4, ρ_{calcd} = 1.154 g·cm^{-3}, μ = 1.463 mm^{-1}, F(000) = 3068, T = 100(2) K, R_1 = 0.0794, wR_2 = 0.1878, 16348 independent reflections [$2\theta \leq 140.152°$] and 1962 parameters.

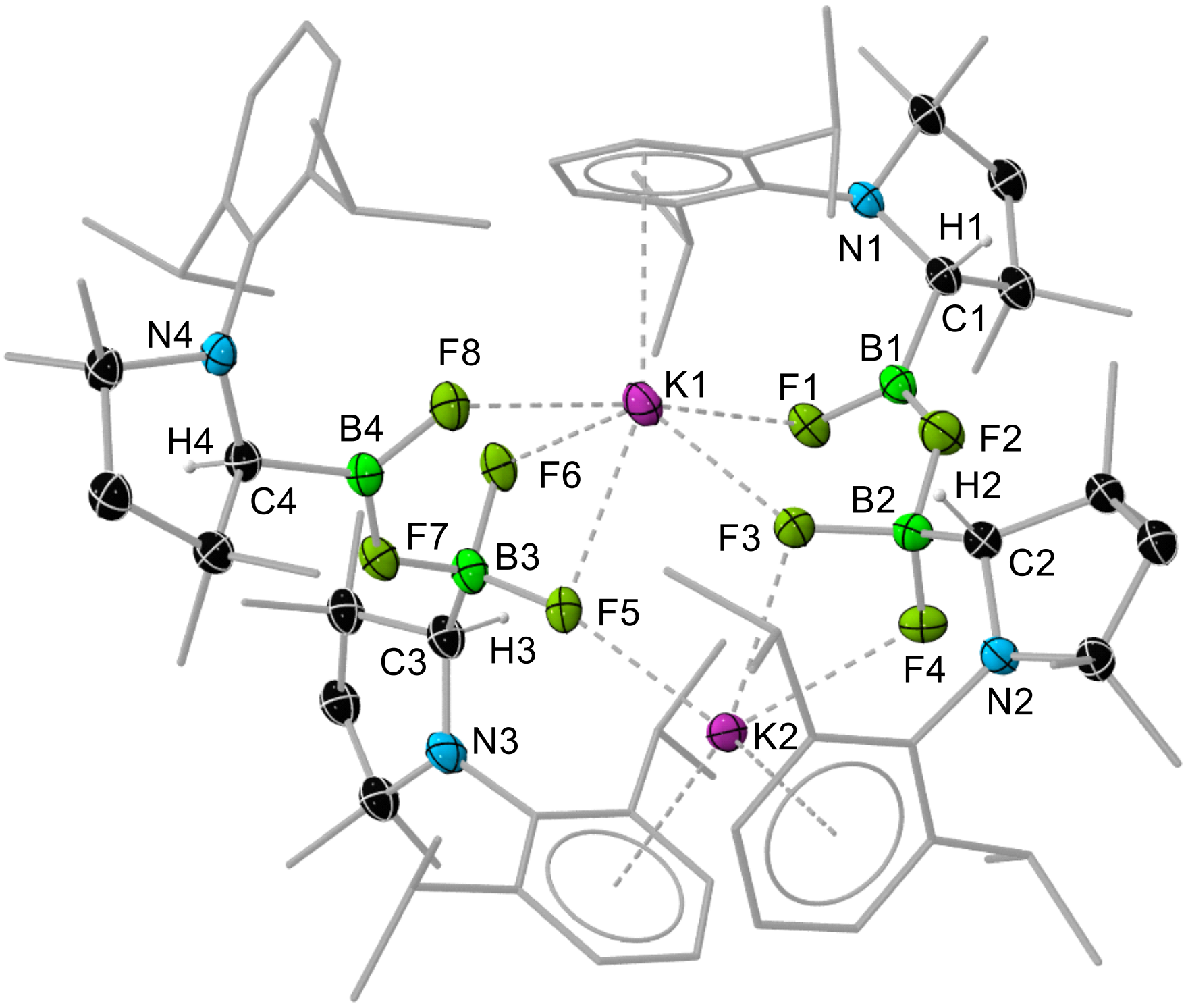

6a

4:1

6b

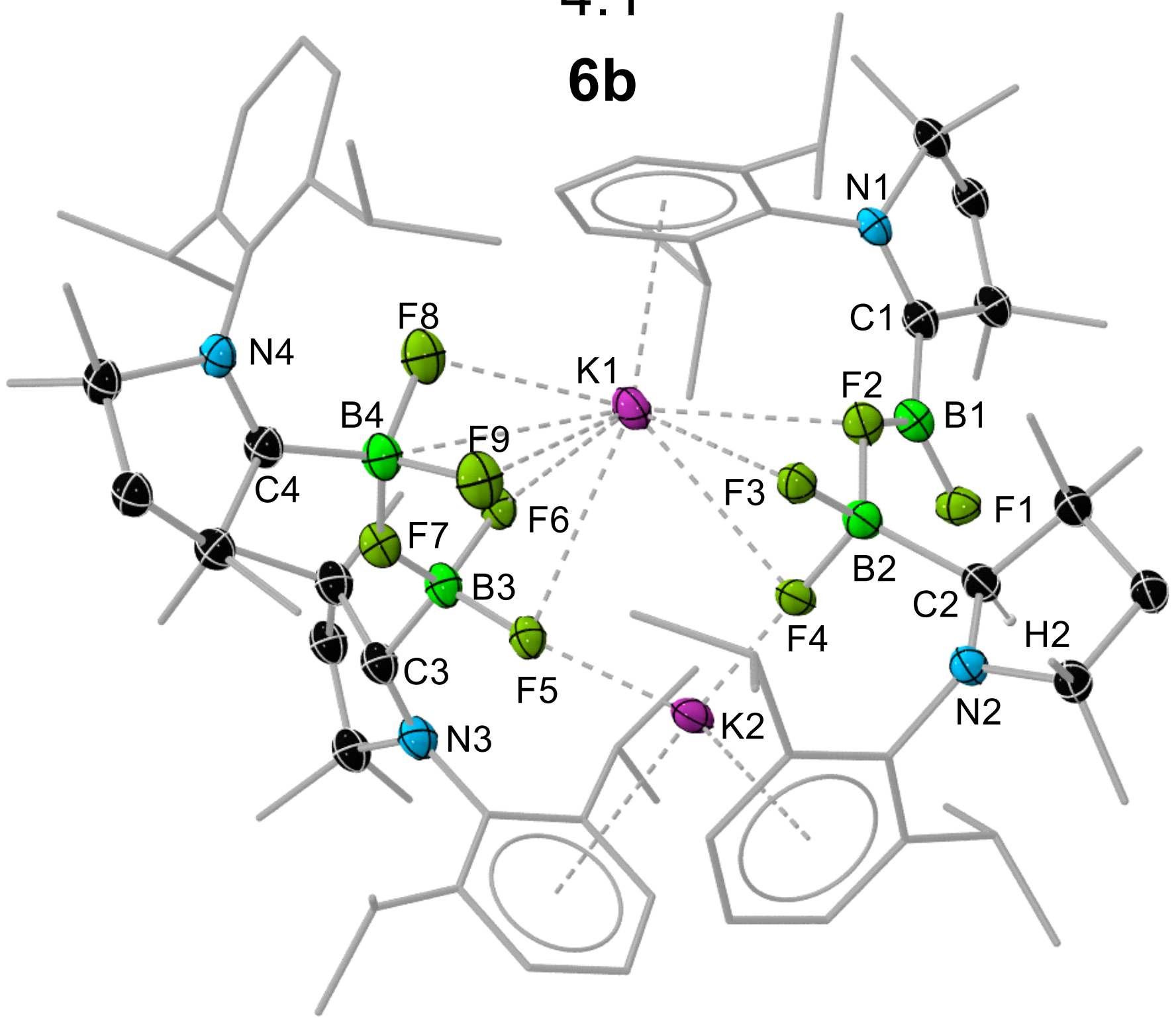

Figure S40. SCXRD-derived centrosymmetric solid-state structure of **6a** and **6b**, overlapping in the asymmetric unit in a 4:1 ratio. Atomic displacement ellipsoids set at 50% probability. Ellipsoids of ligand periphery and hydrogen atoms omitted for clarity, except for CAAC*H* protons.

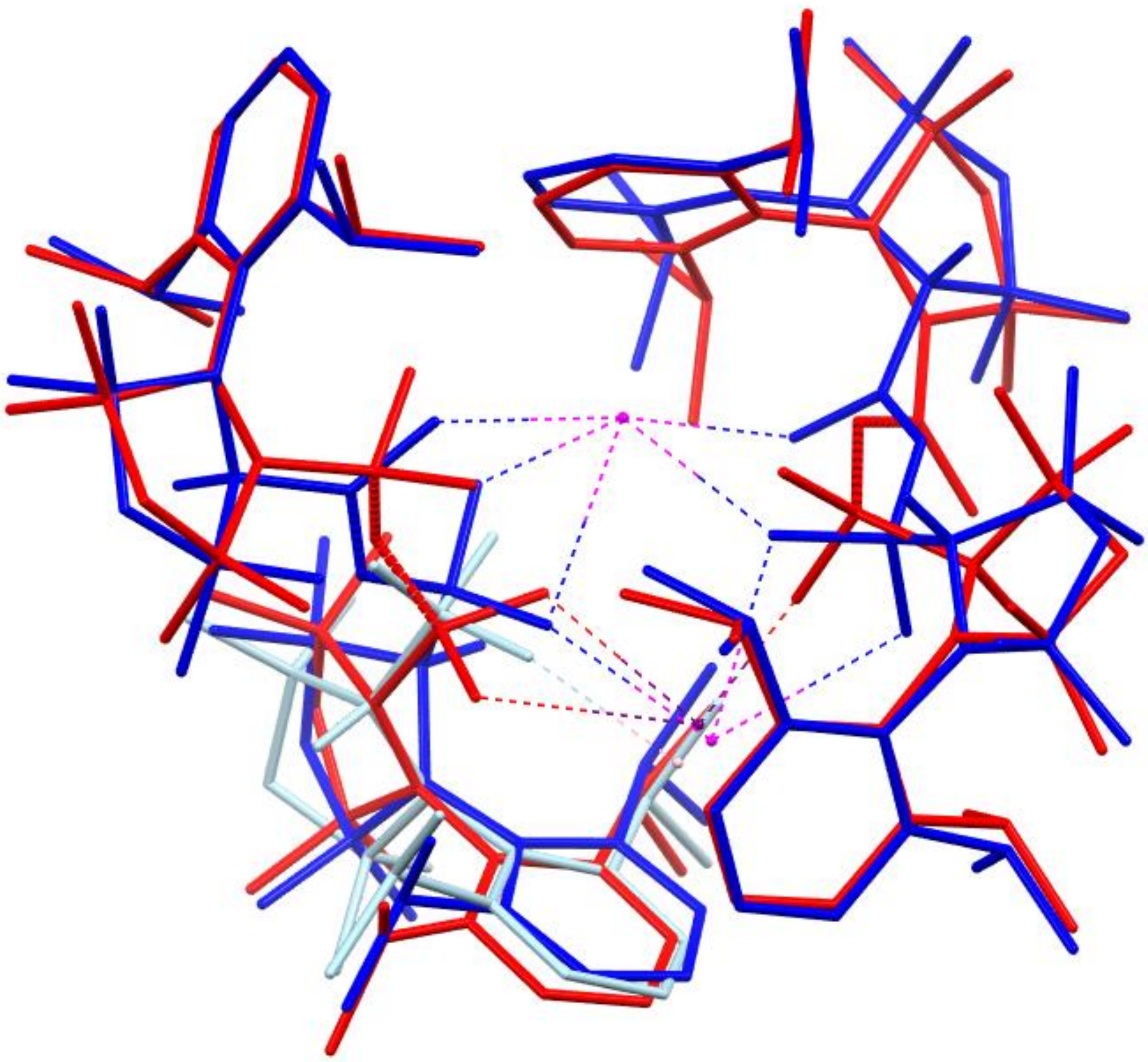

Figure S41. Stick representation of the asymmetric unit, showing the overlap of **6a** (marine blue: main diastereomer, pale blue: minor diastereomer) and **6b**. Hydrogen atoms omitted for clarity, except for CAAC*H* protons.

Compound 7^{Na}

Special refinement details for 7^{Na}: One THF ligand was modelled as twofold disordered in a 71:29 ratio (RESI 31 and 32 THF). ADPs within this disorder were restrained to similarity with SIMU 0.005.

Crystal data for 7^{Na}: $[C_{20}H_{31}BF_2NNa\cdot OC_4H_8]_2$, $M_r = 429.36$, yellow block, $0.202\times0.143\times0.108$ mm^3, triclinic space group $P\bar{1}$, $a = 9.60880(10)$ Å, $b = 14.2431(2)$ Å, $c = 18.9929(4)$ Å, $\alpha = 68.281(2)°$, $\beta = 87.2050(10)°$, $\gamma = 88.0300(10)°$, $V = 2411.58(7)$ Å^3, $Z = 2$, $\rho_{calcd} = 1.183$ g·cm^{-3}, $\mu = 0.802$ mm^{-1}, $F(000) = 928$, $T = 100(2)$ K, $R_1 = 0.0489$, $wR_2 = 0.1203$, 9066 independent reflections [$2\theta \leq 140.120°$] and 603 parameters.

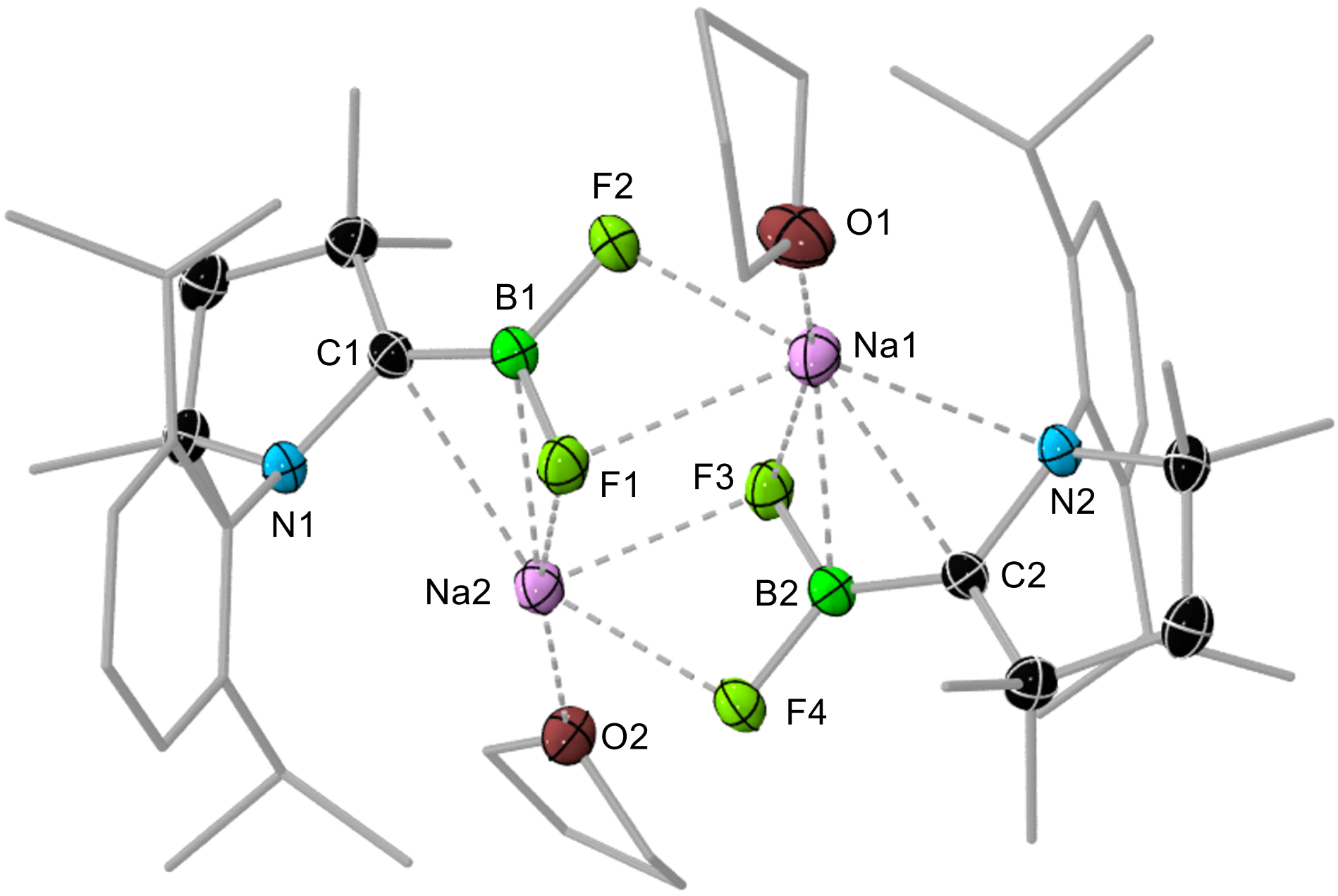

Figure S42. SCXRD-derived solid-state structure of 7^{Na}. Atomic displacement ellipsoids set at 50% probability. Ellipsoids of ligand periphery and hydrogen atoms omitted for clarity.

Compound $8^{n\text{Pr}}$

Special refinement details for $8^{n\text{Pr}}$: Two reflections were removed as outliers (1 5 3, 2 5 2). The NDip moiety was modelled as twofold disordered (RESI 21/22 NDIP) in a 48:52 ratio. Benzene rings within this disorder were idealised with AFIX 66, ADPs restrained to similarity with SIMU 0.005 and 1,2-/1,3-distances with SAME. The *n*Pr substituent was modelled as fourfold disordered in the terminal CH_2CH_3 moiety (RESI 41/42/43/44) with four FVAR summed up to 1.0, and refined to a 67:11:8:14 ratio. ADPs within this disorder were restrained to similarity with SIMU 0.005, 1,2-distances with SAME and 1,2/1,3-distances to C21_3 with SADI.

Crystal data for $8^{n\text{Pr}}$: $C_{23}H_{38}BF_2N$, M_r = 377.35, colourless block, 0.490×0.170×0.150 mm^3, monoclinic space group $P2_1/n$, a = 10.326(3) Å, b = 16.453(3) Å, c = 13.6962(19) Å, β = 101.176(17)°, V = 2282.9(8) Å^3, Z = 4, ρ_{calcd} = 1.098 g·cm^{-3}, μ = 0.579 mm^{-1}, F(000) = 824, T = 100(2) K, R_1 = 0.0827, wR_2 = 0.2381, 4320 independent reflections [$2\theta \leq 140.124°$] and 408 parameters.

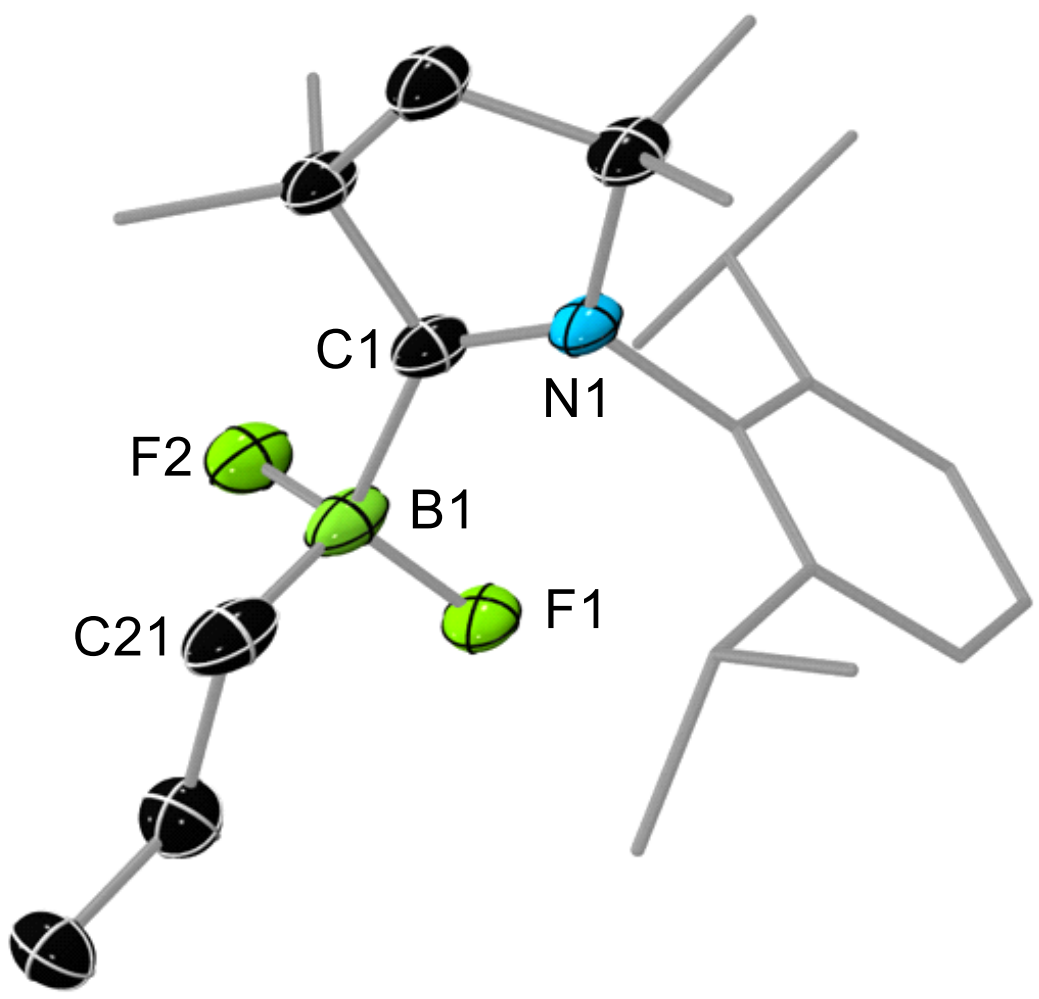

Figure S43. SCXRD-derived solid-state structure of $8^{n\text{Pr}}$. Atomic displacement ellipsoids set at 50% probability. Ellipsoids of ligand periphery and hydrogen atoms omitted for clarity.

Compound 8^{iPr}

Special refinement details for 8^{iPr}: The crystals were long, very thin needles and heavily twinned (up to 6 components). Only the two major components (ca. 40%) were integrated as a pseudo-merohedral twin. Component 2 rotated by 4.57% around [–0.03 0.01 1.00] (reciprocal) or [0.09 0.02 1.00] (direct). Only component 1 kept for refinement as the data for component 2 was of insufficient quality.

Crystal data for 8^{iPr}: $C_{26}H_{45}BF_2N$, M_r = 420.44, colourless needle, 0.234×0.055×0.037 mm^3, monoclinic space group $P2_1/c$, a = 8.0404(2) Å, b = 14.8379(5) Å, c = 21.6242(6) Å, β = 96.759(3)°, V = 2561.89(13) Å^3, Z = 4, ρ_{calcd} = 1.090 g·cm^{-3}, μ = 0.559 mm^{-1}, F(000) = 924, T = 100(2) K, R_1 = 0.1075, wR_2 = 0.2344, 5037 independent reflections [$2\theta \leq 151.114°$] and 282 parameters.

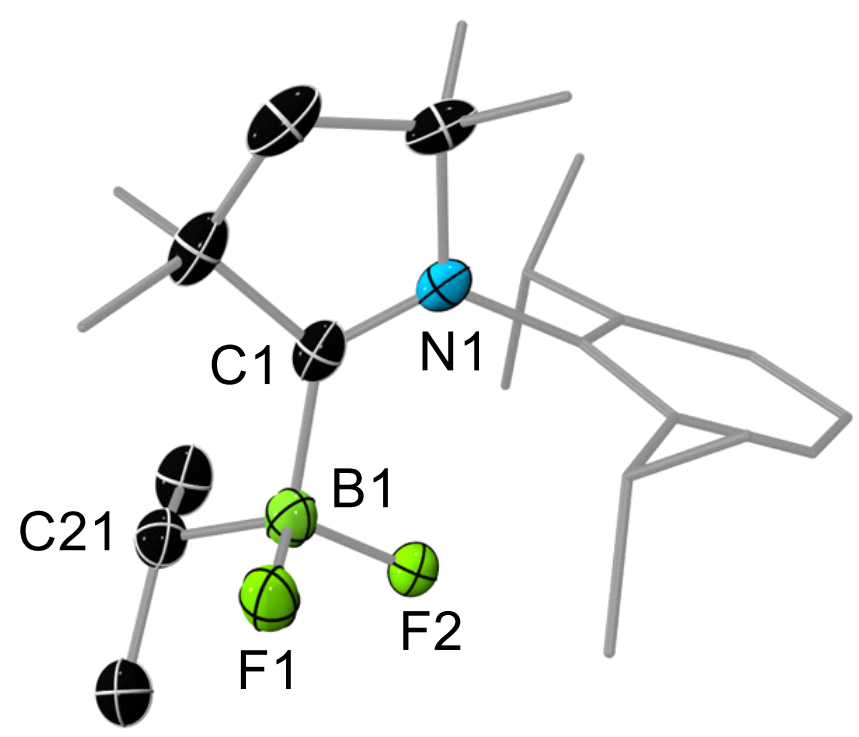

Figure S44. SCXRD-derived solid-state structure of 8^{iPr}. Atomic displacement ellipsoids set at 50% probability. Ellipsoids of ligand periphery and hydrogen atoms omitted for clarity.

Computational details

Density functional theory (DFT) calculations were carried out with Turbomole 7.9[8] as implemented in the user interface TmoleX2024.[9] Optimisations were carried out at the (U)ωB97X[10]-D4[11]-def2-TZVP[12,13] level of theory using the relevant crystallography-derived solid-state structures as starting points, where possible. True minima were confirmed by frequency calculations, which provided no imaginary frequencies. Solvation in benzene (ε =2.274) was taken into account by performing energy calculations with the COSMO-RS (conductor-like screening model – real solvents) solvent model,[14] using a fine cavity and removing the inner cavity. Wiberg bond indices (WBIs), and natural bond orbital (NBO)[16] populations analyses were carried out within the TmoleX2024 interface.

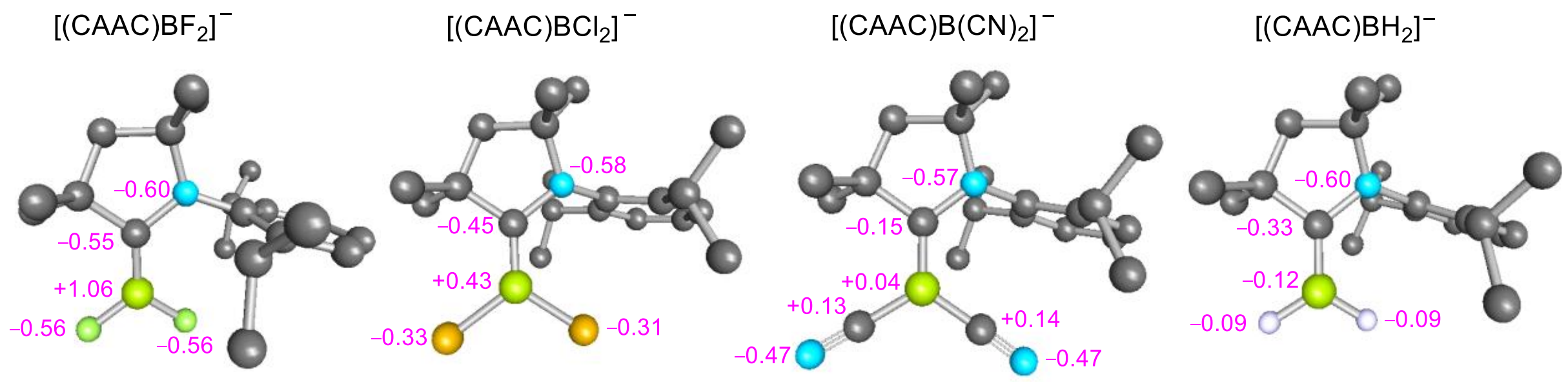

Figure S45. Calculated structures of the naked $[(CAAC)BY_2]^-$ anions (Y = F, Cl, CN, H) at the ωB97X-D4-def2-SVP level of theory, with NBO charges in pink. Ball-and-stick models. Atom colours: hydrogen = white (omitted for clarity, except for boron-bound hydrides), boron = yellow-green, carbon = grey, nitrogen = sky blue, fluorine = pale green.

Table S1. Thermodynamic data for the disproportionation of **7**^{Na} in benzene at rt. E_{COSMO} = SCF energy, G_{corr} = thermodynamic solvent correction, both calculated at the (U)ωB97X-D4-def2-TZVP-COSMO(C_6H_6) level of theory. CP_{298K} = chemical potential at 298 K, obtained from full frequency calculations at the (U)ωB97X-D4-def2-SVP level. E_{lat} = lattice energy of NaF at 298 K.[17] G_{298K} = Gibbs free energy at 298 K = E_{COSMO} + G_{corr} + CP_{298K} (+ E_{lat}).

	**7**^{Na}	→	0.5 **V**	+ **2**	+ 2 THF	+ 2 NaF
E_{COSMO} (Ha)	–2910.43928		–860.49620	–1060.38564	–464.97784	–524.46066
ΣE_{COSMO} (Ha)	–2910.43903			–2910.32335		
ΔE_{COSMO} without taking into account NaF lattice energy: 0.11593 Ha = +72.4 kcal mol^{-1}						
E_{lat} (Ha)						–0.70310
ΔE_{sol} taking into account NaF lattice energy: –0.58718 Ha = –368.5 kcal mol^{-1}						
G_{corr} (Ha)	–0.00599		–0.00254	–0.00365	–0.00459	–0.00397
CP_{298K} (Ha)	1.10246		0.43880	0.43044	0.17905	–0.04000
G_{298K} (Ha)	–2909.34879		–860.06420	–1059.96249	–464.81257	–524.51258
ΣG_{298K} (Ha)	–2909.34879			–2909.35184		
ΔG_{298K} without taking into account NaF lattice energy: –0.00305 Ha = –1.9 kcal mol^{-1}						
ΔG_{298K} taking into account NaF lattice energy: –0.70616 Ha = –443.1 kcal mol^{-1}						

Cartesian coordinates of optimised compounds

2 (doublet state)

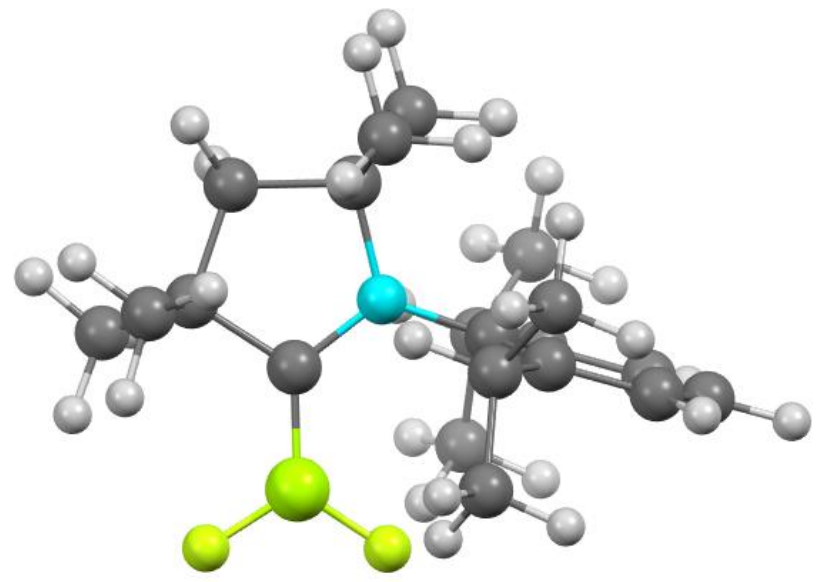

E_{SCF} = –1060.363934964 Ha

ν_{min} = 22.83 cm^{-1}

N	-0.928699	-0.894203	-0.373463
C	1.162708	0.021445	0.222895
C	0.038812	1.014688	0.573103
C	0.385251	-1.218159	-0.181816
C	-1.239117	0.540756	-0.132097
C	2.027287	0.515786	-0.944921
C	2.061024	-0.211315	1.439371
H	-0.125889	0.999928	1.653375
H	0.293903	2.039391	0.295781
C	-2.465824	0.722106	0.757420
C	-1.495031	1.296063	-1.438041
H	2.781156	-0.230818	-1.201213
H	1.431586	0.710498	-1.838539
H	2.543074	1.441016	-0.672965
H	-2.362827	0.885938	-1.956588
H	-0.639785	1.264251	-2.112057
H	-1.705633	2.342940	-1.211590
H	2.503414	0.734893	1.763357
H	1.487084	-0.621028	2.273728
H	2.870437	-0.903888	1.209334
H	-2.325294	0.264779	1.735760
H	-3.359256	0.296908	0.295534
H	-2.641001	1.789029	0.907854
B	1.000938	-2.580705	-0.379877
F	0.319404	-3.695728	-0.702020
F	2.329898	-2.760930	-0.241059
C	-1.919480	-1.823314	-0.834053
C	-2.100627	-2.031294	-2.210508
C	-3.121832	-2.882040	-2.619923
C	-3.922682	-3.535046	-1.703284
C	-3.682643	-3.378286	-0.351470
C	-2.675127	-2.538661	0.108230
C	-1.171100	-1.456579	-3.266632

H	-3.279758	-3.050417	-3.679088
H	-4.715333	-4.191199	-2.043101
H	-4.279096	-3.933972	0.363144
C	-2.373885	-2.510498	1.597177
C	-1.913495	-0.698445	-4.367039
C	-0.317753	-2.569483	-3.882741
H	-0.488338	-0.763683	-2.776669
H	-1.620669	-1.742356	1.774910
C	-1.765603	-3.845013	2.038077
C	-3.603025	-2.183234	2.444738
H	0.402417	-2.147854	-4.587700
H	0.228502	-3.125133	-3.120049
H	-0.941745	-3.282647	-4.427222
H	-2.548682	0.092417	-3.965617
H	-1.199796	-0.242433	-5.056829
H	-2.548015	-1.368711	-4.951041
H	-4.077316	-1.251280	2.133120
H	-3.321611	-2.088471	3.495988
H	-4.352194	-2.975326	2.378673
H	-1.487392	-3.804568	3.093798
H	-2.481760	-4.660718	1.910756
H	-0.877843	-4.089851	1.454027

[(IMe)BF_2]$^•$ (doublet state)

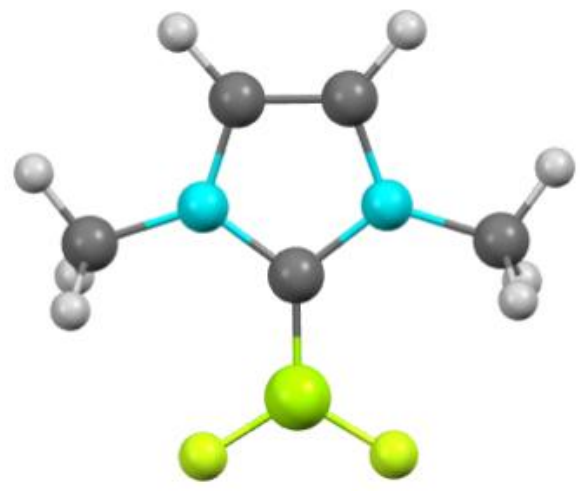

E_{SCF} = –528.95991368275 Ha

ν_{min} = 51.20 cm^{-1}

N	0.009445	0.016203	1.173455
C	1.162630	0.018253	0.388167
N	0.701540	-0.057774	-0.926319
C	-0.678271	-0.104523	-0.927816
C	-1.102001	-0.062855	0.357978
B	2.585891	0.086557	0.852200
H	-1.250270	-0.163067	-1.851406
H	-2.111694	-0.081675	0.762754
C	0.001912	0.045642	2.613477
C	1.550184	-0.056565	-2.090091
H	0.539030	0.928898	2.991085
H	0.486037	-0.850824	3.033298
H	-1.038488	0.084719	2.962921

H	2.259487	-0.897915	-2.061617
H	0.923547	-0.148437	-2.987176
H	2.133308	0.875809	-2.155272
F	2.909968	0.163395	2.162818
F	3.622884	0.075030	-0.015884

[IX]• (doublet state)

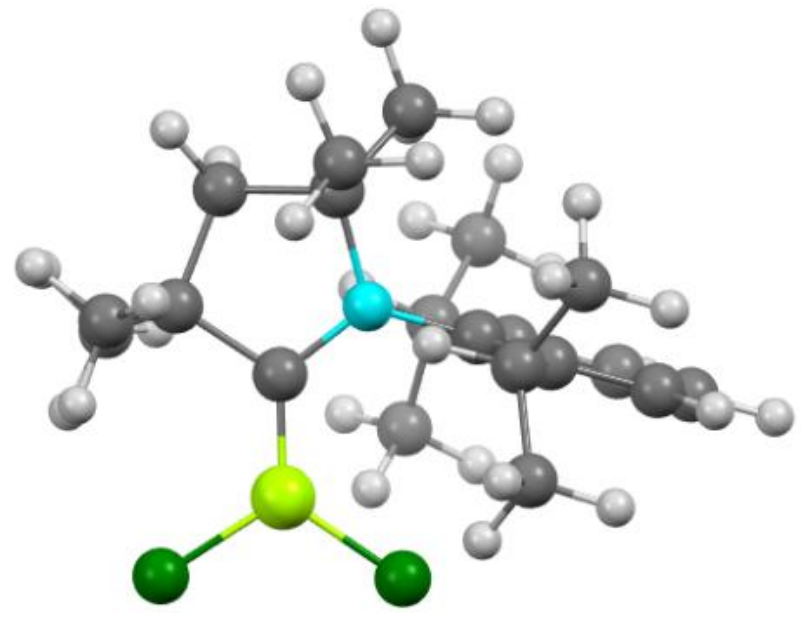

$E_{\mathrm{SCF}} = -1779.81538974321$ Ha

$\nu_{\mathrm{min}} = 20.95\ \mathrm{cm}^{-1}$

Cl	2.708005	5.067498	12.805825
C	3.088195	2.668537	14.457487
N	2.852543	1.747405	13.470291
B	3.002533	4.176028	14.322628
Cl	3.196995	5.249572	15.741959
C	2.800495	0.340273	13.971614
C	3.447597	-0.645178	13.001661
H	2.908988	-0.682522	12.042549
H	3.421470	-1.651676	13.446373
H	4.497213	-0.392710	12.799794
C	1.359273	-0.116187	14.238030
H	0.798506	-0.246043	13.303400
H	0.812554	0.594810	14.873119
H	1.377290	-1.088842	14.753420
C	3.591286	0.468050	15.277211
H	4.653099	0.252858	15.073029
H	3.253962	-0.253484	16.037093
C	3.449841	1.928807	15.755672
C	4.797371	2.391648	16.333079
H	5.582510	2.382927	15.559452
H	5.107064	1.704074	17.137702
H	4.741628	3.404288	16.752145
C	2.363311	2.079103	16.833030
H	2.260976	3.129406	17.139292
H	2.632749	1.487534	17.723307
H	1.380095	1.733335	16.482021
C	2.840970	2.045722	12.062539
C	4.081418	2.183090	11.399678

C	5.423917	2.094618	12.117189
H	5.240571	1.720058	13.135079
C	6.068348	3.479156	12.251907
H	6.314953	3.894308	11.260786
H	7.003208	3.416409	12.831917
H	5.398300	4.194542	12.749294
C	6.390495	1.122976	11.432622
H	5.942238	0.128262	11.284238
H	7.302513	1.001046	12.038212
H	6.704023	1.493876	10.443925
C	4.075739	2.464565	10.032218
H	5.026662	2.582770	9.504761
C	2.885185	2.605600	9.330024
H	2.901757	2.824750	8.258877
C	1.675435	2.482697	9.998689
H	0.740433	2.618153	9.446987
C	1.625677	2.214012	11.369799
C	0.262323	2.175467	12.045962
H	0.425386	1.983109	13.115239
C	-0.465369	3.520463	11.938393
H	-0.686217	3.773454	10.888560
H	0.134329	4.337781	12.362307
H	-1.425433	3.477681	12.477309
C	-0.614743	1.054629	11.476514
H	-0.904723	1.271738	10.435649
H	-1.542195	0.949090	12.061861
H	-0.098884	0.081824	11.471876

7^{Na}

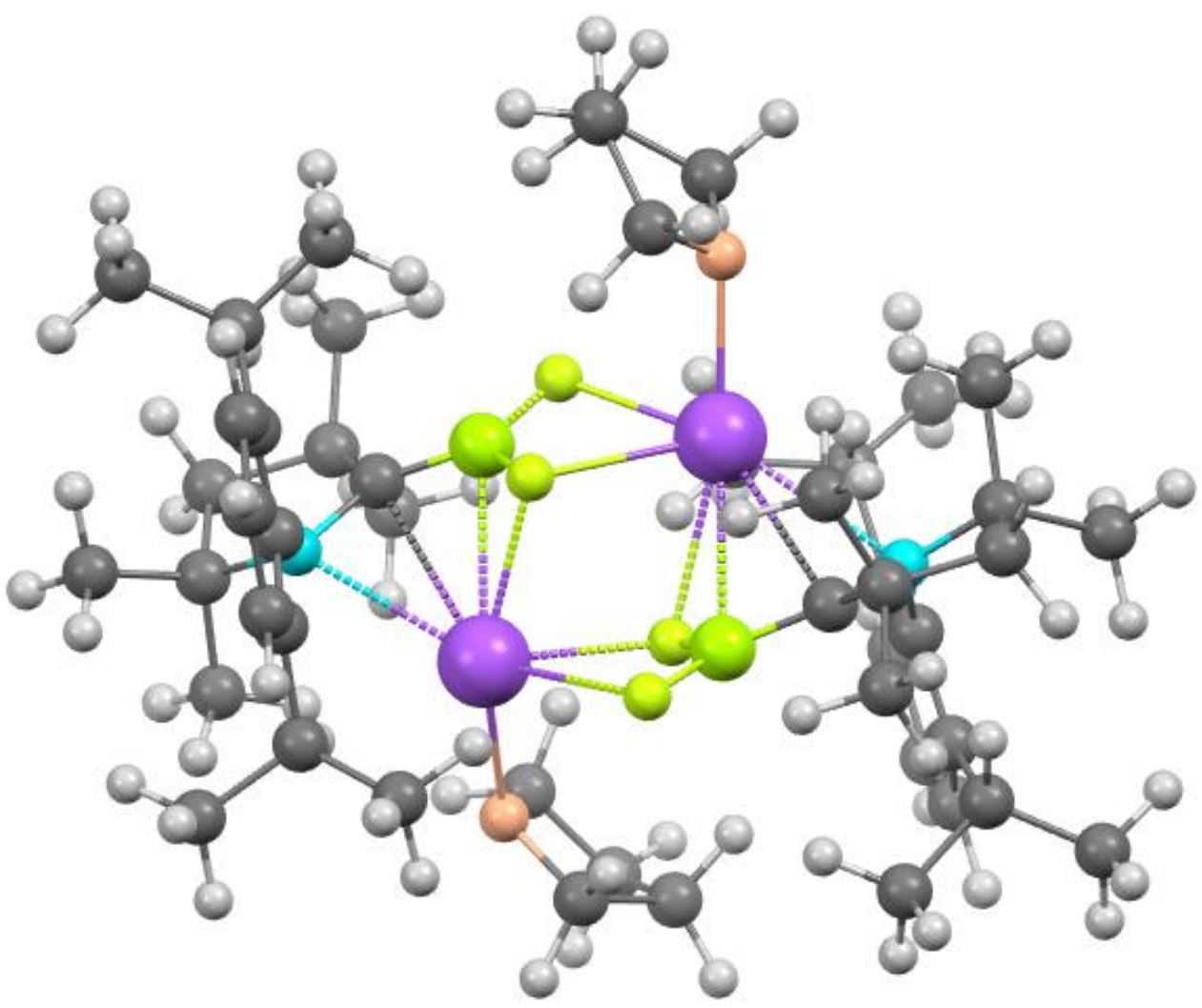

E_{SCF} = –2910.39736964829 Ha

ν_{min} = 14.46 cm^{-1}

F	8.760074	7.913731	4.384113
F	7.945690	7.439653	2.317775
N	7.692482	5.382755	5.583435
C	7.419324	5.801249	4.202719
C	6.760475	4.289076	5.966479
C	6.253356	3.741817	4.614919
H	6.870587	2.888701	4.320464
H	5.226294	3.374705	4.691417
C	6.386306	4.869056	3.570146
C	5.596917	4.837382	6.806844
H	4.864569	4.050097	7.002989
H	5.065314	5.652178	6.313177
H	5.956814	5.199599	7.773675
C	7.428583	3.190118	6.786529
H	6.690510	2.421149	7.023727
H	7.826234	3.577402	7.727495
H	8.245665	2.717013	6.244477
C	6.807069	4.316351	2.202696
H	6.047151	3.634989	1.806894
H	7.751577	3.773726	2.256626
H	6.933063	5.131279	1.486468
C	5.037702	5.581609	3.361160
H	4.298013	4.905454	2.919482
H	5.163383	6.433274	2.687779
H	4.619283	5.954109	4.299879
C	9.056664	5.376662	6.033700
C	10.081835	4.654267	5.380720
C	11.388095	4.756899	5.842657
H	12.170653	4.202998	5.335083
C	11.716335	5.535772	6.936853
H	12.741978	5.594793	7.282678
C	10.714831	6.223744	7.588961
H	10.961477	6.831398	8.453227
C	9.393257	6.159009	7.155487
C	9.836990	3.739551	4.193566
H	8.764480	3.678695	4.044421
C	10.356910	2.320303	4.439132
H	10.039664	1.660767	3.627709
H	9.988338	1.903941	5.378282
H	11.448408	2.292149	4.474437
C	10.447816	4.295113	2.906625
H	10.250531	3.620920	2.069086
H	11.532413	4.398882	3.001288
H	10.024165	5.269917	2.660579
C	8.369387	6.974077	7.924803
H	7.403785	6.794907	7.452226
C	8.231998	6.502721	9.372292
H	7.451236	7.067291	9.888646
H	9.164101	6.642746	9.924612

H	7.974637	5.442143	9.420650
C	8.685307	8.470415	7.863900
H	7.889209	9.061672	8.324743
H	8.806908	8.811775	6.833028
H	9.613109	8.695061	8.395357
B	8.021651	6.965567	3.623961
Na	8.590093	9.805410	2.978961
F	6.318624	9.574267	3.841580
F	6.549255	10.053963	6.049690
N	7.680406	12.284732	3.061273
C	7.449990	11.768018	4.419123
C	8.517854	13.516314	3.143687
C	8.330261	13.984414	4.597961
H	7.481925	14.674112	4.636300
H	9.202866	14.531737	4.964099
C	8.018963	12.744947	5.450954
C	9.993033	13.198800	2.854732
H	10.631629	14.030031	3.163427
H	10.161807	13.034899	1.788861
H	10.322397	12.304746	3.385740
C	8.070719	14.615247	2.185522
H	8.711472	15.489022	2.323298
H	7.041059	14.917713	2.375919
H	8.142565	14.307552	1.142006
C	9.286968	12.205173	6.130858
H	9.689465	12.932154	6.844368
H	10.070573	11.975823	5.408817
H	9.057091	11.286389	6.677013
C	7.033955	13.100788	6.573564
H	7.463324	13.858249	7.237097
H	6.802845	12.220444	7.176598
H	6.094797	13.492923	6.178506
C	6.659867	12.170359	2.057958
C	7.000361	11.621715	0.801251
C	6.015329	11.445395	-0.166866
H	6.286642	11.020367	-1.127099
C	4.701677	11.786127	0.072468
H	3.944194	11.628704	-0.686137
C	4.370811	12.348197	1.290891
H	3.343846	12.646421	1.473460
C	5.321943	12.569511	2.277998
C	8.414440	11.203099	0.422909
H	9.051022	11.336321	1.298130
C	8.491329	9.738769	-0.025209
H	9.534339	9.424480	-0.120295
H	8.021228	9.603882	-1.001397
H	7.985678	9.051301	0.656882
C	9.001727	12.095750	-0.672453
H	10.025556	11.791425	-0.903743

H	9.019932	13.145704	-0.378074
H	8.413797	12.019575	-1.590166
C	4.861414	13.282515	3.536269
H	5.732744	13.440053	4.162554
C	3.871004	12.435396	4.333557
H	3.605955	12.930939	5.272155
H	4.300457	11.461179	4.571778
H	2.948994	12.273691	3.767512
C	4.251282	14.651710	3.227344
H	4.022768	15.179400	4.156838
H	3.320487	14.560905	2.662714
H	4.929674	15.275417	2.642401
B	6.786960	10.546865	4.757202
Na	6.411998	7.766008	5.549491
O	4.246946	8.073597	6.294904
C	3.304635	8.644689	5.368528
H	2.709886	7.836315	4.939158
H	3.864362	9.133494	4.564822
C	2.477443	9.641887	6.170223
H	1.588877	9.159123	6.582942
H	2.156896	10.487115	5.561823
C	3.436068	10.038693	7.290484
H	2.929445	10.432643	8.171076
H	4.156080	10.780271	6.937499
C	4.143831	8.724910	7.566644
H	5.151231	8.854638	7.969780
H	3.567774	8.089060	8.247810
O	10.893385	9.500438	2.952229
C	11.493690	9.687837	4.246154
H	10.718804	9.573424	5.011786
H	11.893528	10.702800	4.295451
C	12.559098	8.610551	4.378858
H	13.512562	8.961018	3.976576
H	12.708116	8.309060	5.415681
C	11.984136	7.492709	3.516023
H	12.732943	6.778795	3.172783
H	11.218512	6.949897	4.072344
C	11.356988	8.270355	2.372864
H	12.089175	8.504535	1.592450
H	10.505263	7.760485	1.915865

[(CAAC)BF$_2$]$^{-}$

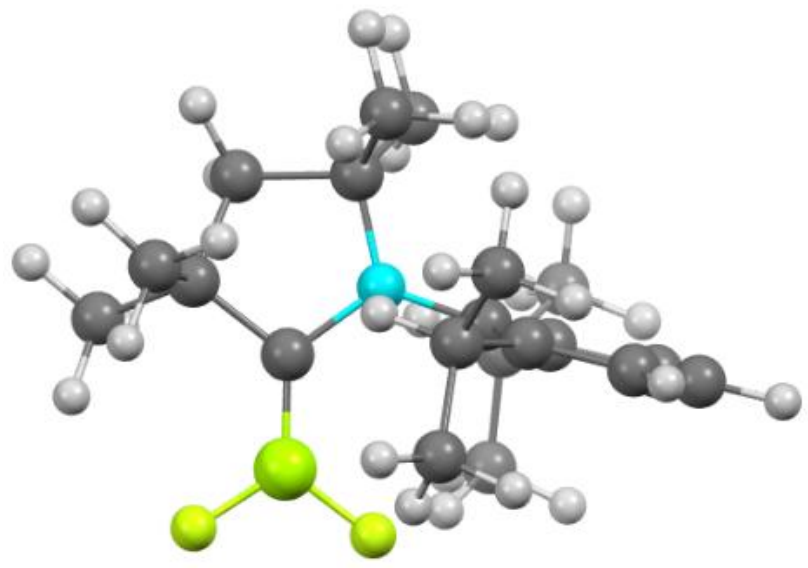

E_{SCF} = –1059.238485619 Ha

$\nu_{\min}$ = 28.52 cm^{-1}

N	-0.987398	-0.919141	-0.245171
C	1.179202	0.064062	0.065586
C	0.054798	0.994527	0.573237
C	0.442595	-1.246494	-0.169885
C	-1.276699	0.493210	-0.020675
C	1.838877	0.621552	-1.213381
C	2.278188	-0.058783	1.129897
H	-0.000699	0.905901	1.671565
H	0.233526	2.059823	0.339964
C	-2.429368	0.720921	0.971982
C	-1.649238	1.256755	-1.310510
H	2.626691	-0.072623	-1.546649
H	1.118971	0.716064	-2.040480
H	2.297393	1.616390	-1.046659
H	-2.554208	0.819908	-1.764719
H	-0.843851	1.220880	-2.056545
H	-1.860554	2.317087	-1.089085
H	2.779417	0.911191	1.312360
H	1.850521	-0.420639	2.078363
H	3.032637	-0.791981	0.808971
H	-2.208801	0.264529	1.948183
H	-3.368546	0.285878	0.591136
H	-2.596786	1.800163	1.129152
B	1.037837	-2.562674	-0.284978
F	0.344105	-3.754597	-0.509831
F	2.409309	-2.802366	-0.229865
C	-1.951544	-1.798060	-0.782652
C	-2.072215	-2.022319	-2.176925
C	-3.123820	-2.805921	-2.660777
C	-4.037707	-3.401840	-1.797789
C	-3.861219	-3.269055	-0.425513
C	-2.813733	-2.502673	0.095042
C	-1.020206	-1.512667	-3.148443
H	-3.222930	-2.961441	-3.740852
H	-4.863688	-4.001243	-2.194523
H	-4.534338	-3.797621	0.258687

C	-2.516773	-2.556400	1.585781
C	-1.601130	-0.720086	-4.320528
C	-0.171373	-2.689801	-3.642709
H	-0.347955	-0.863041	-2.573993
H	-1.744311	-1.797725	1.773469
C	-1.891619	-3.914631	1.926012
C	-3.730592	-2.267419	2.469452
H	0.692281	-2.330693	-4.227690
H	0.200914	-3.273282	-2.788170
H	-0.764047	-3.361898	-4.288989
H	-2.206170	0.133219	-3.975777
H	-0.790780	-0.326103	-4.957061
H	-2.242602	-1.348875	-4.961560
H	-4.184484	-1.291664	2.234017
H	-3.440852	-2.259375	3.533839
H	-4.512635	-3.037329	2.351972
H	-1.572772	-3.943578	2.982257
H	-2.614047	-4.735059	1.766637
H	-1.015829	-4.097421	1.285766

[(CAAC)BCl₂]⁻

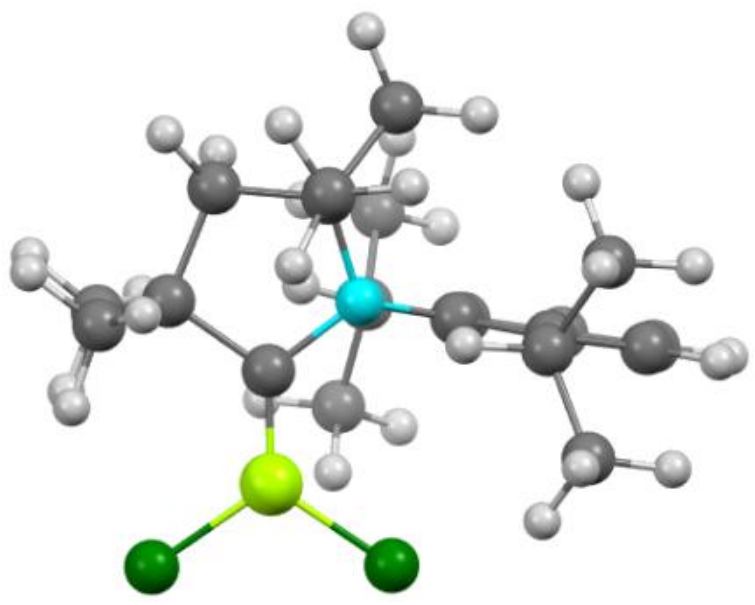

$E_{SCF} = -1779.856472246$ Ha

$\nu_{min} = 42.58$ cm^{-1}

Cl	2.289678	5.126845	13.100847
C	3.093655	2.697091	14.515685
N	2.689456	1.785089	13.455487
B	2.951260	4.133588	14.502168
Cl	3.524047	5.231389	15.876638
C	2.559673	0.415772	13.966113
C	2.904455	-0.643465	12.918443
H	2.199498	-0.624617	12.072528
H	2.865099	-1.645701	13.376313
H	3.915467	-0.486545	12.511966
C	1.150110	0.123796	14.522188
H	0.426103	-0.042723	13.710950
H	0.793878	0.971752	15.124679
H	1.154502	-0.779255	15.156421

C	3.598027	0.409106	15.107071
H	4.579213	0.142811	14.676038
H	3.370904	-0.344182	15.881844
C	3.657936	1.845196	15.672916
C	5.108778	2.191321	16.054825
H	5.783767	2.108945	15.188128
H	5.482975	1.508295	16.840733
H	5.171855	3.222492	16.431298
C	2.810267	1.971686	16.952326
H	2.816255	3.015245	17.300221
H	3.211682	1.329766	17.759337
H	1.762945	1.685847	16.777395
C	2.812854	2.080604	12.076486
C	4.078850	2.303425	11.476004
C	5.359421	2.286353	12.290668
H	5.082226	1.967915	13.301103
C	5.949986	3.694159	12.405006
H	6.317516	4.049856	11.426047
H	6.798182	3.708169	13.110558
H	5.189057	4.402015	12.766104
C	6.389422	1.290756	11.750851
H	5.969036	0.274151	11.678104
H	7.268503	1.247088	12.416041
H	6.750008	1.573378	10.747188
C	4.162582	2.557526	10.105755
H	5.146397	2.728775	9.654842
C	3.028693	2.583040	9.301200
H	3.114081	2.769638	8.225984
C	1.785403	2.388629	9.887494
H	0.883498	2.441693	9.267324
C	1.655698	2.165030	11.261794
C	0.256409	2.088202	11.852566
H	0.387114	2.008707	12.940329
C	-0.537658	3.370856	11.587075
H	-0.716073	3.523276	10.508559
H	0.006781	4.243008	11.977155
H	-1.522178	3.323146	12.083114
C	-0.515265	0.863536	11.351974
H	-0.705061	0.930932	10.266669
H	-1.493615	0.780087	11.855539
H	0.038512	-0.072135	11.529505

[(CAAC)B(CN)₂]⁻

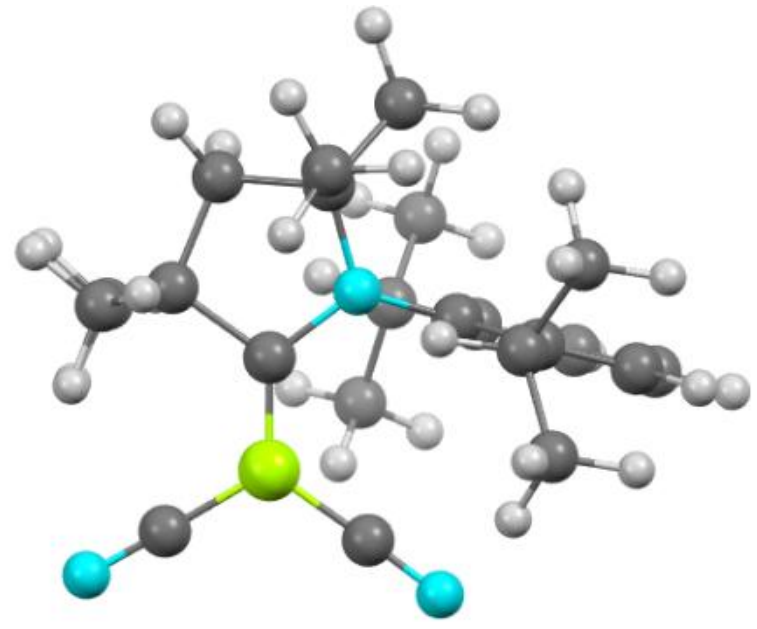

$E_{SCF} = -1045.208712238$ Ha

$\nu_{min} = 28.44$ cm^{-1}

C	2.438737	4.933320	13.185011
C	3.074487	2.680222	14.485532
N	2.759855	1.744788	13.461330
B	2.960214	4.130331	14.420147
C	3.378950	5.018344	15.639447
C	2.656278	0.368299	13.968681
C	3.119832	-0.665741	12.943717
H	2.474182	-0.665294	12.051105
H	3.088567	-1.672909	13.390137
H	4.149673	-0.464868	12.613389
C	1.227833	0.008055	14.422948
H	0.558490	-0.143004	13.565205
H	0.802756	0.807328	15.046893
H	1.233955	-0.924486	15.011987
C	3.599981	0.422822	15.183072
H	4.619924	0.170809	14.844853
H	3.326753	-0.309347	15.961643
C	3.576265	1.877168	15.703981
C	4.992492	2.286265	16.142727
H	5.699710	2.231961	15.299525
H	5.356726	1.610964	16.938671
H	5.008655	3.314225	16.531247
C	2.645399	2.033668	16.918162
H	2.630559	3.082201	17.250537
H	2.995547	1.408524	17.759641
H	1.610650	1.744199	16.683565
C	2.819057	2.072975	12.077651
C	4.077920	2.274670	11.456533
C	5.385672	2.198015	12.229479
H	5.141988	1.856226	13.243559
C	6.030338	3.581519	12.356204
H	6.341138	3.965524	11.369248
H	6.925840	3.536494	12.998880
H	5.325205	4.299034	12.800712
C	6.368358	1.193500	11.619782
H	5.920990	0.191257	11.520951

H	7.268005	1.103988	12.251274
H	6.701240	1.507119	10.616296
C	4.129289	2.579281	10.095359
H	5.102367	2.742919	9.620273
C	2.971554	2.679222	9.332812
H	3.030659	2.914132	8.265644
C	1.739754	2.503157	9.945509
H	0.824184	2.616476	9.355998
C	1.640564	2.222376	11.311937
C	0.254456	2.135542	11.930430
H	0.400122	2.043446	13.016142
C	-0.564537	3.407394	11.684133
H	-0.831543	3.512011	10.618396
H	-0.002149	4.305122	11.979426
H	-1.506646	3.370261	12.256630
C	-0.508930	0.907481	11.421779
H	-0.736217	1.007856	10.346708
H	-1.467545	0.788769	11.954830
H	0.069264	-0.021922	11.545586
N	2.050233	5.560069	12.285400
N	3.685477	5.658241	16.561633

$[(CAAC)BH_2]^-$

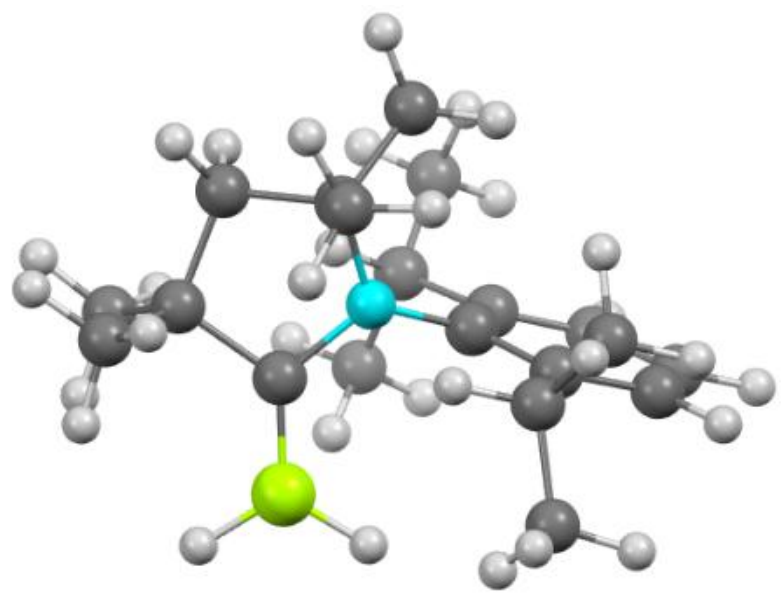

E_{SCF} = –860.8131539768 Ha

ν_{min} = 31.38 cm^{-1}

H	2.246493	4.618218	13.344229
C	2.961263	2.669833	14.464306
N	2.810247	1.671321	13.420303
B	2.674752	4.091673	14.371138
H	2.906108	4.831291	15.331341
C	2.718078	0.320955	13.958234
C	3.248322	-0.734761	12.987198
H	2.639324	-0.772133	12.068909
H	3.220190	-1.731720	13.457563
H	4.287507	-0.521091	12.692947
C	1.282048	-0.070378	14.369446
H	0.651323	-0.251927	13.485772

H	0.821424	0.737982	14.956895
H	1.280152	-0.990594	14.979171
C	3.612924	0.443517	15.212666
H	4.652944	0.214548	14.919986
H	3.334830	-0.280188	15.999599
C	3.515424	1.914884	15.688061
C	4.902927	2.431942	16.102119
H	5.609249	2.379905	15.257611
H	5.325781	1.849284	16.944452
H	4.832106	3.487777	16.406152
C	2.591328	2.047597	16.908889
H	2.467999	3.112106	17.160790
H	3.002722	1.517526	17.790063
H	1.588011	1.645112	16.702150
C	2.856396	2.003585	12.051253
C	4.107174	2.182378	11.402920
C	5.410566	2.097440	12.179342
H	5.156807	1.683681	13.164931
C	5.989516	3.495776	12.412446
H	6.253767	3.980080	11.455479
H	6.903048	3.442964	13.030051
H	5.250783	4.120155	12.938389
C	6.437298	1.172037	11.521856
H	6.021465	0.167325	11.340154
H	7.325317	1.061961	12.166710
H	6.785385	1.567082	10.552275
C	4.144194	2.476433	10.038836
H	5.112392	2.607408	9.543047
C	2.973813	2.607533	9.296505
H	3.020029	2.829586	8.225345
C	1.748397	2.476781	9.935250
H	0.824032	2.615049	9.362847
C	1.670677	2.199746	11.305550
C	0.306512	2.177249	11.974495
H	0.488984	1.997013	13.042477
C	-0.385069	3.539202	11.866557
H	-0.596683	3.807174	10.816279
H	0.252536	4.318462	12.309459
H	-1.345481	3.528588	12.409711
C	-0.586647	1.063978	11.419753
H	-0.843038	1.247038	10.361680
H	-1.532714	0.999437	11.983797
H	-0.091549	0.080794	11.471214

V

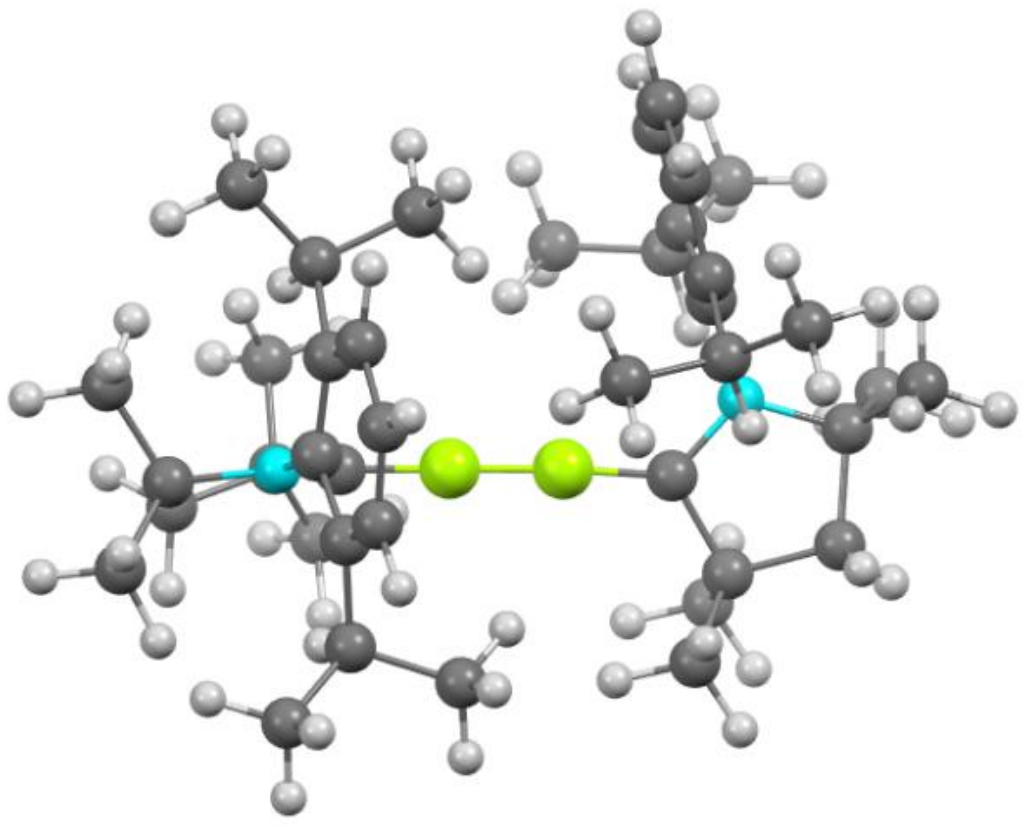

E_{SCF} = –1720.96348976716 Ha

ν_{min} = 15.31 cm^{-1}

N	5.900014	1.697947	4.411118
B	5.233833	5.254683	2.833000
N	4.238972	7.562387	2.853459
C	3.200211	7.225134	3.778148
C	6.183985	2.382971	5.636174
C	5.448026	2.362327	3.311699
C	5.182066	6.653899	2.485348
C	1.981542	6.704952	3.315898
C	5.021366	1.338330	2.265437
C	5.149160	2.602973	6.564177
C	6.212579	7.341227	1.593949
C	7.478405	2.869120	5.883389
C	3.437480	7.381982	5.156256
C	1.746846	6.318058	1.866587
H	2.620116	6.623674	1.291359
C	6.012192	0.227515	4.248644
C	3.690236	2.304817	6.263167
H	3.644967	1.779677	5.311261
C	4.412886	8.908876	2.257977
C	1.166246	6.681931	5.589955
H	0.367001	6.486404	6.295514
C	6.753601	3.603788	8.068116
H	6.979805	4.063001	9.023523
C	5.464522	3.197982	7.780079
H	4.678492	3.364976	8.508047
C	1.637305	4.795882	1.741964
H	1.546916	4.505635	0.692077
H	0.757804	4.420167	2.272158
H	2.523132	4.313893	2.159311
C	2.397655	7.120230	6.039799
H	2.561668	7.244370	7.104363
C	4.797698	7.750208	5.725668
H	5.454617	8.006007	4.893337

C	0.972598	6.455947	4.241707
H	0.022388	6.062632	3.898052
C	7.742423	3.460545	7.114766
H	8.740393	3.830235	7.322332
C	8.577530	2.862816	4.835528
H	8.197039	2.347737	3.952828
C	5.422848	6.547511	6.438291
H	5.523638	5.694603	5.764896
H	4.813509	6.236660	7.291665
H	6.417199	6.802630	6.814141
C	2.906061	3.608133	6.090842
H	3.336608	4.229076	5.303135
H	2.899261	4.189088	7.017472
H	1.867807	3.394877	5.823419
C	5.437544	1.736387	0.852181
H	5.159328	0.953415	0.141007
H	6.515486	1.895391	0.792489
H	4.945796	2.664420	0.552265
C	5.759244	0.073829	2.740467
H	5.201129	-0.837604	2.516061
H	6.719697	0.007614	2.223051
C	3.031558	1.427987	7.328088
H	2.014103	1.169811	7.025451
H	2.963529	1.950337	8.285163
H	3.581222	0.500374	7.494959
C	3.497810	1.155044	2.298468
H	2.997301	2.068142	1.972405
H	3.131832	0.917821	3.297952
H	3.204562	0.342232	1.628061
C	0.511560	6.990218	1.268028
H	0.545461	8.076250	1.369207
H	-0.402629	6.642339	1.754711
H	0.428182	6.750720	0.205520
C	4.729453	8.948725	6.673244
H	5.735680	9.248890	6.974526
H	4.178114	8.701967	7.583435
H	4.238949	9.808036	6.212747
C	9.844063	2.151430	5.311881
H	10.581142	2.112578	4.506347
H	9.642683	1.130357	5.638460
H	10.303016	2.683036	6.148777
C	4.992747	-0.534304	5.099058
H	3.963719	-0.293297	4.836673
H	5.140204	-0.315503	6.158207
H	5.133035	-1.607843	4.958189
C	8.903540	4.297835	4.412319
H	9.629343	4.297427	3.595017
H	9.334676	4.863413	5.243000
H	8.004430	4.815382	4.074303

C	7.400149	-0.283140	4.627561
H	7.448186	-1.360717	4.457173
H	7.612073	-0.100810	5.683426
H	8.177474	0.188808	4.027497
B	5.352159	3.788860	3.124600
C	5.870709	8.831094	1.781699
H	6.030120	9.408065	0.868510
H	6.518222	9.252769	2.554959
C	4.215408	10.025149	3.278751
H	4.936590	9.959509	4.091642
H	3.208761	10.003213	3.701821
H	4.351037	10.989381	2.785054
C	3.436708	9.153422	1.103464
H	3.636382	10.128491	0.654052
H	2.407954	9.158102	1.466926
H	3.521122	8.397713	0.323323
C	7.642523	7.037729	2.037217
H	7.863545	5.973654	1.929747
H	7.795735	7.308650	3.084001
H	8.354603	7.599136	1.425610
C	6.041746	6.887611	0.138490
H	5.022073	7.033277	-0.219992
H	6.272883	5.825289	0.047758
H	6.717238	7.450535	-0.511982

THF

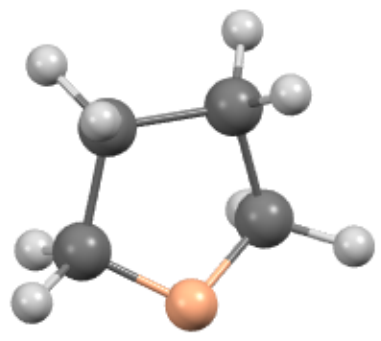

E_{SCF} = –232.48009125080 Ha

ν_{min} = 32.46 cm^{-1}

O	-0.824640	-0.926030	-0.575690
C	1.023307	-0.032596	0.554144
C	-0.126158	0.995702	0.587322
C	0.585581	-1.014508	-0.536344
C	-1.122538	0.450159	-0.444447
H	1.988706	0.420765	0.328563
H	1.111500	-0.543379	1.512986
H	-0.578228	1.040924	1.577844
H	0.205426	2.001532	0.328817
H	0.854779	-2.050889	-0.334535
H	1.005631	-0.726342	-1.510843
H	-2.165823	0.542772	-0.144008

H	-0.991456	0.952972	-1.412991

NaF

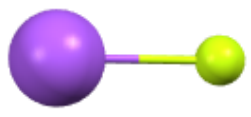

E_{SCF} = – 262.19107157761 Ha

v_{min} = 533.03 cm^{-1}

Na	-0.159009	0.000000	0.000000
F	-2.090991	0.000000	0.000000

References

1. S. Stoll and A. Schweiger, *J. Magn. Reson.*, 2006, **178**, 42–55.
2. S. K. Sarkar, M. M. Siddiqui, S. Kundu, M. Ghosh, J. Kretsch, P. Stollberg, R. Herbst-Irmer, D. Stalke, A. C. Stückl, B. Schwederski, W. Kaim, S. Ghorai, E. D. Jemmis and H. W. Roesky, *Dalton Trans*., 2019, **48**, 8551–8555
3. D. M. Ottmers and H. F. Rase, *Carbon*, 1966, **4**, 125–127.
4. J. Böhnke, H. Braunschweig, W. C. Ewing, C. Hörl, T. Kramer, I. Kummenacher, J. Mies and A. Vargas, *Angew. Chem. Int. Ed.*, 2014, **53**, 9082–9085.
5. G. Sheldrick, *Acta Cryst.*, 2015, **A71**, 3–8.
6. G. Sheldrick, *Acta Cryst.*, 2008, **A64**, 112–122.
7. A. L. Spek, *Acta Cryst.*, 2015, **C71**, 9–18.
8. TURBOMOLE V7.8.1, A development of University of Karlsruhe, Forschungszentrum Karlsruhe GmbH, 1989−2007, TURBOMOLE GmbH, 2007, http://www.turbo-mole.com.TmoleX2024.
9. TmoleX2024. Dassault Systèmes: Versailles.
10. J.-D. Chai and M. Head-Gordon, *J. Chem. Phys.*, 2008, **128**, No. 084106.
11. E. Caldeweyher, S. Ehlert, A. Hansen, H. Neugebauer, S. Spicher, C. Bannwarth and S. Grimme, *J. Chem. Phys*., 2019, **150**, No. 154122.
12. F. Weigend, F. *Phys. Chem., Chem. Phys*., 2006, **8**, 1057–1065.
13. F. Weigend and R. Ahlrichs, *Phys. Chem., Chem. Phys*., 2005, **7**, 3297–3305.
14. a) F Eckert and A. Klamt, *AIChE J.,* 2002, **48**, 369–385; b) A. Klamt and G. Schüürmann, *J. Chem. Soc., Perkin Trans. 2*, 1993, **5**, 799–805.
15. K. B. Wiberg, *Tetrahedron*, 1968, **24**, 1083–1096.
16. A. E. Reed, R. B. Weinstock and F. Weinhold, *J. Chem. Phys.,* 1985, **83**, 735–746.
17. H. D. B. Jenkins, in *CRC Handbook of Chemistry and Physics*, edited by D. R. Lide, CRC Press, Boca Raton, FL, 1998.
